# Sustainable Synthesis of 1,2-Disubstituted Benzimidazoles as Promising α-Glucosidase Inhibitors: In Vitro and In Silico Evaluation

**DOI:** 10.3390/ph18101469

**Published:** 2025-09-30

**Authors:** Graziella Tocco, Antonio Laus, Cristina Manis, Pierluigi Caboni, Antonella Fais, Benedetta Era

**Affiliations:** Department of Life and Environmental Sciences, University of Cagliari, Cittadella Universitaria di Monserrato, 09042 Monserrato, Italy; laus@crs4.it (A.L.); cristina.manis@unica.it (C.M.); caboni@unica.it (P.C.); fais@unica.it (A.F.); era@unica.it (B.E.)

**Keywords:** 1,2-disubstituted benzimidazoles, α-glucosidase inhibitors, antioxidant activity, cytotoxicity, in silico experiments

## Abstract

**Background:** Inhibiting α-glucosidase and α-amylase is a well-established strategy for managing postprandial hyperglycemia in type 2 diabetes mellitus. However, the adverse effects of current α-glucosidase inhibitors (α-GIs) underscore the need for safer alternatives. **Methods:** This study introduces an efficient, metal-free, and environmentally friendly protocol for the selective, high-yield synthesis of 1,2-disubstituted benzimidazoles. The reaction between *o*-phenylenediamine and various aromatic aldehydes proceeds smoothly in water at room temperature, using cost-effective and eco-friendly catalysts such as acetylsalicylic acid (ASA) or salicylic acid (SA). The methodology exhibits broad versatility, enabled by the use of different *o*-phenylenediamines and a wide range of aromatic and heteroaromatic aldehydes. **Results:** Selected compounds were assessed for their inhibitory activity against α-glucosidase and α-amylase. While all exhibited low α-amylase inhibition, several showed significant α-glucosidase inhibition, with compounds **8s** (IC_50_ = 0.39 ± 0.04 μM), **8k** (IC_50_ = 7.4 ± 1.6 μM) and **8r** (IC_50_ = 13.8 ± 2.7 μM) emerging as the most promising candidates. Notably, none of these compounds affected Caco-2 cell viability at concentrations up to 30 μM. Additionally, compounds **8r** and **8s** exhibited antioxidant properties, which may be relevant in counteracting the excessive production of free radicals associated with diabetes. Preliminary molecular docking and 500 ns molecular dynamics (MD) simulations were carried out on compounds **3k**, **8i**, **8k**, and **8p**–**8s** to support and interpret the experimental biological findings qualitatively.

## 1. Introduction

**Figure 1:**
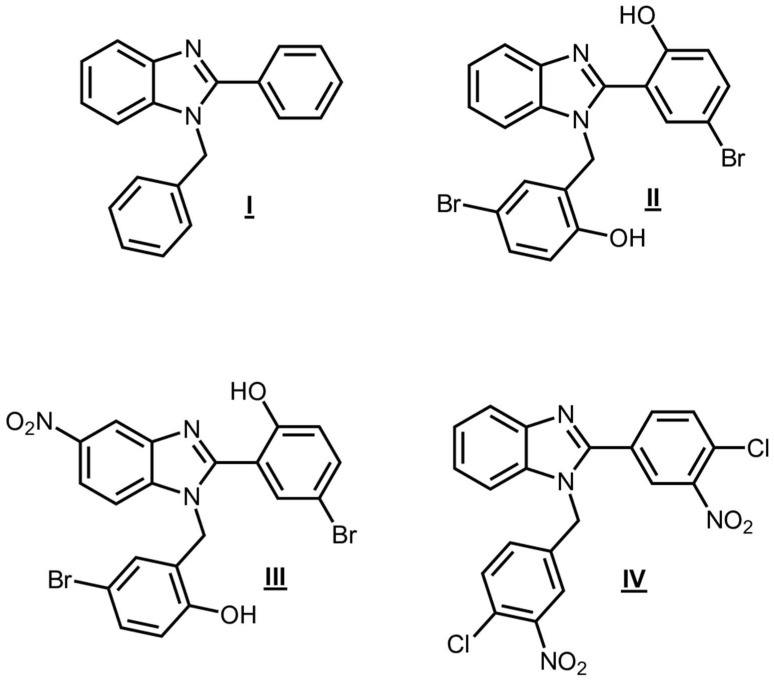
Examples of biologically active 1,2-dibenzimidazoles equally disubstituted at benzylic carbon atom/C-2.

The benzimidazole core plays a major role among the nitrogen-containing heterocycles, as witnessed by its ubiquitous presence in a wide variety of biologically active compounds such as antiviral [1], anti-bacterial [2], antidiabetic [3], anti-neoplastic [4], antimycotics [5], and anti-inflammatory drugs [6]. In recent years, increasing attention has been directed toward 1,2-disubstituted benzimidazoles due to their broad applicability and structural versatility. Many clinically approved 1,2 disubstituted benzimidazoles feature distinct substituents at the benzylic carbon atom and C-2. However, several other notable compounds with similar disubstitution patterns have also been developed. In this context, compounds **I** and **II**, particularly active against *V. cholerae*, *B. cereus*, and *S. dysenteriae* [7], compound **III**, a potent hPreP agonist [8], and compound **IV** which improves the activity of insulin-degrading enzyme (IDE) specifically toward β-amyloid degradation [9], represent significant examples (Figure 1). Consequently, several synthetic routes have been developed. The most common and widely diffused methodology involves the cyclocondensation of *o*-phenylenediamine and various aromatic aldehydes, carried out under very different conditions, including the use of strong Brønsted acids [10,11], rare-earth and post-transition metal-based catalysts [12,13,14], metal-oxide nanomaterials [15,16], gold-complexes [17], and many others.

Sorrowfully, although providing 2-aryl-1-arylmethyl-1H-benzimidazoles in good to excellent yields, many protocols suffer from some issues related to the use of harsh acids or metal-containing catalysts, hazardous organic solvents, heating and prolonged reaction times, poor regioselectivity and limited reaction scope.

In this context, the development of an eco-friendly and sustainable synthetic method remains a constant priority. A key challenge in establishing a reliable green procedure lies in designing a synthesis that can be performed in water, as highlighted by several recently proposed protocols. Nevertheless, some of these methods only utilize water as a co-solvent [18,19], or require elevated temperatures and prolonged reaction times [20], involve multi-step workup processes [21], or predominantly yield 2-substituted benzimidazoles [22].

Thus, to meet the challenge of a successful green methodology, and inspired by imine formation in water [23], we developed a fast, one-pot, room-temperature method for selectively producing 1,2-disubstituted benzimidazoles in water using eco-friendly ASA and SA catalysts. Selected compounds were subsequently evaluated for their inhibitory activity against α-glucosidase (EC 3.2.1.3) and α-amylase (EC 3.2.1.1), also testing their antioxidant properties and assessing their cytotoxicity. To qualitatively support the biological results, molecular docking and 500 ns MD simulations were also performed.

Though SA and ASA are renowned primarily for their notable pharmacological features, their recent emergence as effective organocatalysts has garnered considerable attention. In this context, while the application of ASA as a catalyst is uncommon, SA has proven to be an effective catalyst for the synthesis of 3-substituted-4-arylmethylideneisoxazol-5(4H)-ones [24], 2,3-dihydroquinazolin-4(1H)-ones [25], 3,4-dihydropyrimidin-2-(1H)-ones/thiones [26], 2,4,6-triaryl pyridine, 2-amino-3-cyanopyridine [27], 2,5-dimethylpyrroles via a solvent-free Paal–Knorr [28], and dihydropyridines and acridinediones via Hantzsch reaction [29].

From the array of synthesized compounds, our interest was notably captured by IDE-active compound **IV** and its derivatives. In line with our ongoing research into biologically active heterocycles [30,31], specifically focusing on anti-diabetic agents [32], we proceeded to evaluate the inhibitory activity of selected compounds against α-glucosidase (EC 3.2.1.3) and α-amylase (EC 3.2.1.1), key enzymes implicated in carbohydrate metabolism. Inhibiting these enzymes, as with acarbose, is a clinically established strategy to manage postprandial blood glucose levels in diabetes treatment [33]. To further investigate the biological findings, molecular docking and single 500 ns MD simulations were performed for the eight tested compounds, offering mechanistic insights into potential ligand/α-glucosidase interactions. Moreover, the most active compounds **8s**, **8k,** and **8r**, showing low micromolar inhibitory activity, were tested to assess their antioxidant properties and their impact on the viability of Caco-2 cells (human colon adenocarcinoma cell line). The results demonstrated that these inhibitors exhibited no cytotoxic effects on cell viability, even at concentrations up to 30 μM. Notably, compounds **8r** and **8s** also exhibited antioxidant activity, which may help counteract the excess free radicals commonly associated with hyperglycemia and diabetes.

## 2. Results and Discussion

### 2.1. Chemistry

**Scheme 1:**
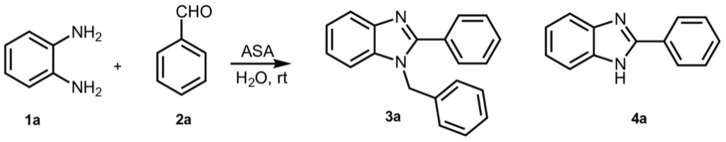
Reaction of **1a** and **2a** in the presence of ASA.

Many reports indicate that the reaction between *o*-phenylenediamines and aldehydes frequently involves the formation of an imine intermediate. Thus, envisaging a similar pathway, our primary aim was to verify the ability of ASA to catalyze the condensation process. The reaction of *o*-phenylenediamine **1a** and benzaldehyde **2a** was designed as model to set up the optimal reaction conditions (Scheme 1), and the variation in catalyst concentration was the first investigated parameter (Table S1, Supplementary Material).

As expected, the reaction did not occur in the absence of a catalyst; however, when using ASA in slight excess (1.26 eq) or in a stoichiometric ratio, the reaction was completed within 3 and 9 min, respectively, resulting solely in the formation of 1-benzyl-2-phenyl-1H-benzo[*d*]imidazole **3a**. Interestingly, reducing the amount of ASA to 0.1 equivalents seemed not to affect yields and selectivity, as the reaction gave rise only to compound **3a** (92% yield in 15 min) (Table S1, Supplementary Material). To demonstrate the versatility of our method, we tested various carbonyl substrates using different aromatic and heteroaromatic aldehydes, as detailed in Table 1. ASA demonstrated to be a valid and versatile reaction mediator. Indeed, all experiments conducted using 1.26 equivalents of ASA resulted only in the formation of 3 derivatives in moderate to excellent yields, with reaction times depending on steric and electronic factors. Interestingly, electron donating groups positively affected aldehyde reactivity, as all the electron rich aldehydes gave the best results in terms of time and yield, apart from 1H-indole-3-carbaldehyde **2b** and piperonal **2c**. In that case, the notable difference in reactivity is likely attributed to steric factors that may hinder the formation of the diimine intermediate, consequently influencing both reaction time and yield. This is particularly evident comparing entries 2 and 9. In fact, the mere substitution of a benzodioxol ring in **2c** with a 3-hydroxy-4-methoxy phenyl in **2j** dramatically sped up the reaction, and compound **3j** was obtained in 4 min. Consequently, aldehyde **2j** was selected to perform the reaction with 0.1 equivalents of ASA. As already observed with benzaldehyde **2a**, the use of ASA in catalytic amounts slowed down the reaction, which was complete in 40 min.

Encouraged by these results, we hypothesized that if ASA was effective in synthesizing 1,2-disubstituted benzimidazoles, SA might offer even greater potential. In fact, SA not only possesses higher acidity than ASA but also exhibits a superior ability to donate hydrogen bonds, as demonstrated in the acid-catalyzed Paal–Knorr synthesis of pyrroles [28]. This enhanced hydrogen-bonding capacity, particularly between the hydroxyl group and the carboxylate ion, likely contributes to the stabilization of the conjugate base, thereby facilitating more efficient proton release. Again, to determine the ideal reaction conditions, the reaction between *o*-phenylenediamine **1a** and benzaldehyde **2a** was studied under different concentration of SA. The outcomes overwhelmingly confirmed our hypothesis on the improved catalytic activity of SA. In fact, the reaction went to completion in 1 min in the presence of SA versus 15 min in the presence of ASA, at the same catalyst concentration (0.1 eq.). Notably, even with only 0.02 equivalents of SA, compound **3a** was obtained in quantitative yield within 16 min (Table S2, Supplementary Material). As a result, we kept exploring SA’s catalytic ability in reactions involving various aromatic aldehydes, employing 0.05 equivalents of catalyst. As shown in Table 2, all reactions proceeded efficiently and selectively, affording 2-aryl-1-arylmethyl-1H-benzimidazoles in high yields under mild conditions. Consistent with prior observations, electron-rich aldehydes exhibited a faster reaction rate, although steric hindrance remained a significant influencing factor. Furthermore, it is interesting that in the presence of pyridine-3-carbaldehyde **2m** or strongly deactivated aldehydes such as **2d** and **2k**, the formation, albeit to a lesser extent, of 2-substituted benzimidazole derivatives of the 4-series was also observed.

Then, we sought to assess the reactivity of substituted 1,2-phenylenediamines with the intention of broadening the scope of the protocol. We firstly focused on the reactivity of 4-nitrophenylenediamine **1b**. Unexpectedly, the reactions with benzaldehyde **2a** and 2-methoxybenzaldehyde **2i** produced 1-benzyl-5-nitro-2-phenyl-1*H*-benzo[*d*]imidazole **5a** in low yield and 1-(2-methoxybenzyl)-2-(2-methoxyphenyl)-5-nitro-1*H*-benzo[*d*]imidazole **5i** in trace amounts, primarily leading to the formation of 2-aryl-1H-benzimidazoles **6a** and **6i**, respectively (Table S3, Supplementary Material). Moreover, the reactions occurred at a significantly slower rate compared to those involving *o*-phenylenediamine **2a**. The explanation for this trend could lie in the decreased nucleophilicity of the amine, probably leading to a predominant monoimine intermediate, thereby justifying the predominant formation of **6**-series compounds. In fact, in the most plausible mechanism for the formation of 2-aryl-1-arylmethyl-1H-benzimidazoles and 2-aryl-1H-benzimidazoles, it is proposed that each aldehydic carbonyl group—activated, respectively, by SA or ASA—is rapidly attacked by a 1,2-phenylenediamine derivative. This nucleophilic addition results in the formation of diimine intermediates, which then undergo intramolecular cyclization to yield 1,2-disubstituted benzimidazoles. Similarly, 2-substituted benzimidazoles are formed through an analogous pathway [30,34] (Figure S1, Supplementary Material). Thus, all the factors leading to a reduced reactivity of the reactants would be decisive for the progression and selectivity of the reaction. This phenomenon becomes increasingly evident when a strongly deactivated aldehyde such as 4-chloro-3-nitrobenzaldehyde **2k** is used. In this scenario, the formation of monosubstituted benzimidazole is not achievable, and the reaction proceeded in a completely selective manner, yielding 2-[(4-chloro-3-nitrobenzyliden)amino]-5-nitroaniline **7**, regardless of the catalyst concentration employed (Table S4, Supplementary Material).

A distinct situation was observed when 3-methyl-*o*-phenylenediamine **2c** was employed. In this instance, the use of an activated amine promoted the reaction, leading to the formation of 2-aryl-1-arylmethyl-4-methyl-1H-benzimidazoles exclusively [30] (Table 3), as also validated by 2D-Noesy NMR experiments (see Supplementary Material).

Furthermore, the proposed protocol seeks to diminish reliance on chromatographic techniques by prioritizing alternative purification strategies, such as precipitation and crystallization. In fact, the use of alternative purification methods instead of chromatographic columns presents a variety of benefits, particularly concerning economic efficiency and environmental sustainability.

### 2.2. Biology

As highlighted in the introduction, the benzimidazole scaffold is a key structural component in numerous compounds exhibiting antidiabetic properties.

Employing a virtual screening approach, 1-(4-chloro-3-nitrobenzyl)-2-(4-chloro-3-nitrophenyl)-1H-benzo[d]imidazole **3k** was identified as a regulatory agent of IDE, an enzyme involved in the degradation of various proteins such as insulin and β-amyloid. Notably, in vitro studies have also shown that compound **3k** is able to actively stimulate IDE activity not only towards insulin but especially towards β-amyloid protein [9].

The intriguing findings prompted us to investigate the potential effects of compound **3k** and its derivatives on α−amylase and α-glucosidase. In fact, inhibiting these enzymes is a major strategy for type 2 diabetes patients to maintain proper blood glucose levels. Our initial efforts were directed toward evaluating the inhibitory activity against α-glucosidase. Compounds **3k**, **8k**, and the previously synthesized 5-chloro-1-(4-chloro-3-nitrobenzyl)-2-(4-chloro-3-nitrophenyl)-1H-benzo[d]imidazole **10** [30], were selected for early testing to investigate the influence of substituents on the benzene ring of the benzimidazole scaffold (Table 4).

The initial experiments identified compound **8k** as the most significant, exhibiting a percentage of 71.2% inhibition at a concentration of 50 μM (Table 4), and an IC_50_ value of 7.1 μM (Table 5). Consequently, keeping the methyl group on the benzimidazole benzene ring fixed, the focus shifted to investigating the substitution of the other aromatic rings. The derivatives **8i** and **8p–s** were then synthesized to assess whether variations in the substitution of the aldehydic aromatic rings might boost the inhibitory activity against α-glucosidase.

All compounds exhibited inhibitory activity; however, compounds **8k** and **8s** were the most effective in inhibiting α-glucosidase, achieving inhibition in the low micromolar range. In particular, compound **8s** displayed a percentage of inhibition of 97.5% inhibition at a concentration of 50 μM (Table 4), and an IC_50_ value of 0.35 μM (Table 5). Structurally, it seemed that the presence of multiple OH groups on both rings may be a key factor contributing to the activity, as previously observed [32].

Noteworthy, nearly all compounds evaluated against α-amylase, at the same concentration used for glucosidase (50 μM), exhibited minimal activity, indicating a significant selectivity for α-glucosidase. The finding is promising as it has been reported that excessive inhibition of pancreatic α-amylase might trigger abnormal bacterial fermentation of undigested carbohydrates in the colon [35].

#### 2.2.1. Cell Viability

**Figure 2:**
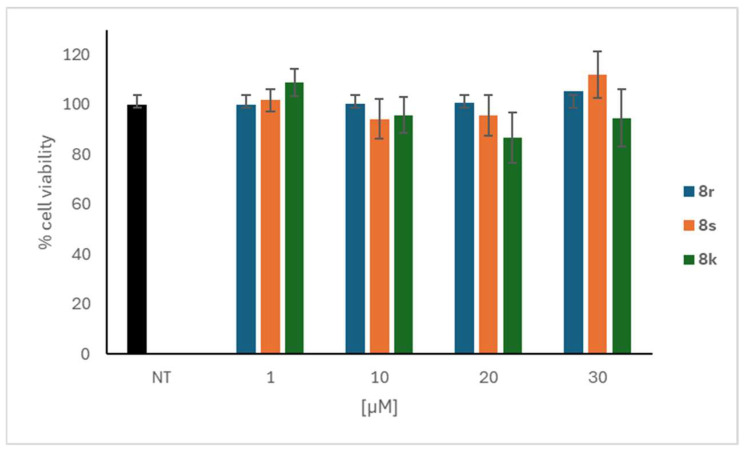
Effect of **8r, 8s** and **8k** on Caco-2 cell viability. After 24 h of incubation with the compounds at different concentrations, it ranges from 0 (NT) to 30 µM. Data represent the mean (±standard deviation, SD) of six independent experiments.

The assessment of the cytotoxicity of compounds **8k**, **8r** and **8s**, was then carried out to evaluate the safety profile of these molecules (Figure 2).

Cells were treated with different concentrations of the compounds (1–30 μM) for 24 h and examined using the MTT assay. Remarkably, these inhibitors demonstrated a complete absence of cytotoxicity in CaCo-2 cells not only at the concentrations required to effectively inhibit α-glucosidase activity, but even at the highest concentrations tested.

#### 2.2.2. Antioxidant Capacity

Clinical studies established that, in diabetic states, free radicals are excessively generated through various pathways, including glucose oxidation, non-enzymatic glycation of proteins, and the subsequent oxidative degradation of these glycated proteins [36]. Additionally, hyperglycemia has been shown to impair endogenous antioxidant defense mechanisms via multiple routes during the progression of diabetes [37]. Consequently, the development of compounds capable of inhibiting α-glucosidase while simultaneously exhibiting antioxidant properties is of therapeutic interest.

The antioxidant capacity of the most promising compounds **8k**, **8r**, and **8s**, was thoroughly evaluated using both 2,2′-azino-bis(3-ethylbenzothiazoline-6-sulfonic acid (ABTS) and 2,2-Diphenyl-1-picrylhydrazyl (DPPH) assays. EC_50_ values are reported in Table 6. Notably, compounds **8r** and **8s** exhibited a remarkable ability to quench both ABTS and DPPH radicals, outperforming the standard antioxidant Trolox in both assays. In contrast, compound **8k** showed no detectable antioxidant activity, underscoring the critical influence of the polyphenolic cores on radical scavenging efficacy.

### 2.3. In Silico Study

#### 2.3.1. Preliminary Modeling Hypothesis

In the absence of experimentally resolved structures for the ligand–enzyme complexes, we carried out exploratory molecular docking followed by 500 ns molecular dynamics (MD) simulations for each ligand. These simulations were designed to generate qualitative hypotheses regarding plausible binding modes. Given the current lack of experimental validation, the resulting models should be interpreted as indicative rather than definitive.

#### 2.3.2. Molecular Docking Analysis

Molecular docking was employed to propose potential binding modes of the compounds to α-glucosidase and to rank the predicted poses. The docking analysis revealed that, except for ligand **8i**, all compounds occupy the same catalytic pocket. While docking provides an initial approximation of binding affinity, the observed partial correlation between docking scores and biological activity highlights the limitations of single-snapshot scoring methods. This emphasizes the need of further experimental structural validation to confirm the predicted binding interactions and to enhance the reliability of computational modeling approaches (Table 7).

**Figure 3:**
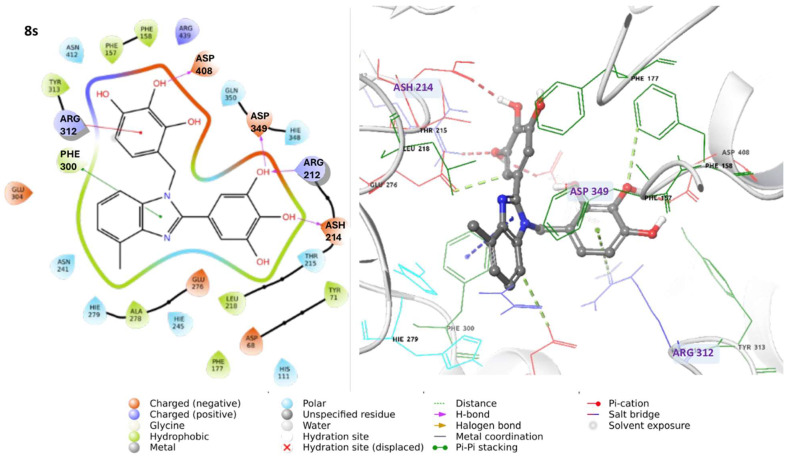
Two-dimensional and 3D representations of the docking pose of compound **8s** within α-glucosidase active site.

Regarding the most active compounds, compound **8s** forms hydrogen bonds with ASP349, ARG212, and ASH214 (protonated aspartate), along with π-cation interactions involving ARG312 and π-π stacking with PHE300 (Figure 3). These interactions engage key residues within the enzyme’s active site, suggesting a strong binding affinity.

**Figure 4:**
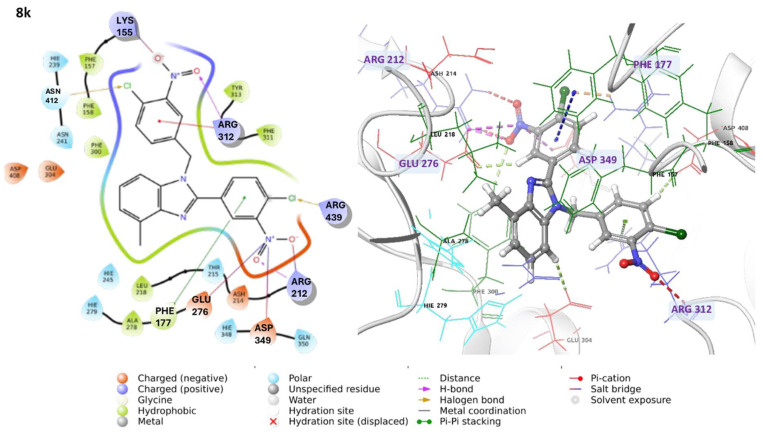
Two-dimensional and 3D representations of the docking pose of compound **8k** within α-glucosidase active site.

Similarly, compound **8k** forms hydrogen bonds with ASP349, ARG212, and ARG312, and established π-cation interactions with ARG312. It also displays a broad spectrum of molecular interactions within the binding pocket, including salt bridges with LYS155, ARG212, GLU276, and ASP349, π-π stacking with PHE177, and halogen bonds with ASN412 and ARG439 (Figure 4).

**Figure 5:**
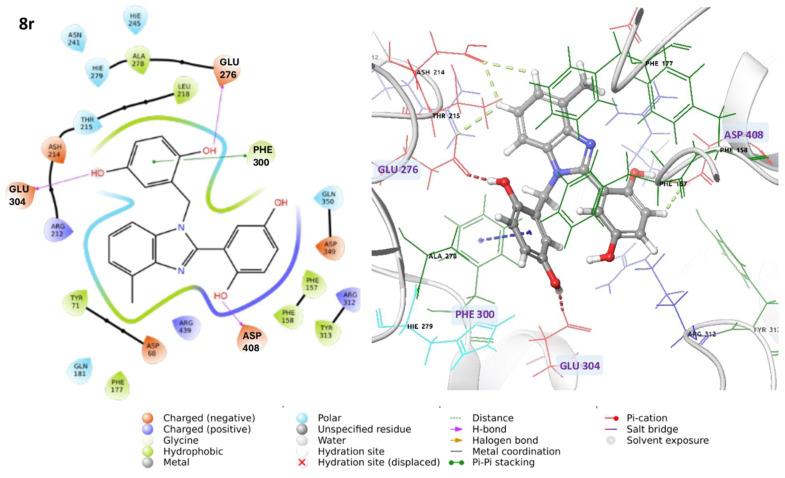
Two-dimensional and 3D representations of the docking pose of compound **8r** within α-glucosidase active site.

The combination of multiple electrostatic, hydrophobic, and halogen interactions may contribute to its interesting biological activity. Compound **8r** engages in hydrogen bonds with ASP408, GLU276, and GLU304, and π-π interactions with PHE300 (Figure 5). Notably, the interactions with acidic and aromatic residues contribute to the stability of the complex.

Regarding the other tested compounds, **8q** participates in π-cation interactions with ARG312, π-π stacking with PHE177, and forms three halogen bonds with ARG212, ARG312, and ASN412 (Figure S2, Supplementary Material). Interestingly, the absence of direct hydrogen bonds may account for its lower activity compared to the other compounds. In contrast, compound **8i** binds to a distinct pocket, forming hydrogen bonds with ASN241 and π-π interactions with HIE279 (histidine tautomer Nε) (Figure S3, Supplementary Material). This alternative binding site may explain its reduced efficacy as an inhibitor.

Compound **10** forms hydrogen bonds with ARG212 and HIE348, salt bridges with ARG212, GLU276, and GLU304, π-cation interactions with ARG312, and π-π stacking with PHE300 (Figure S4, Supplementary Material). Despite the presence of multiple interactions, its lower affinity may stem from the nature of these contacts or from the ligand’s conformation. Compound **3k** shows limited activity, likely due to the absence of interactions with key catalytic residues. It forms hydrogen bonds with HIE348, salt bridges with GLU276 and ASP349, π-cation interactions with ARG312 and ARG349, and a halogen bond with ASH214 (Figure S5, Supplementary Material).

In the case of compound **8p**, hydrogen bonds are observed with ASP408 and ASH214, salt bridges with ASP68 and GLU304, π-cation interactions with ARG312 and PHE177, and π-π stacking with PHE300 (Figure S6, Supplementary Material). However, the ligand appears to form intramolecular interactions that may hinder its ability to effectively engage the enzyme.

In summary, compounds **8s** and **8k**, which exhibited the highest inhibitory activity in biological assays, establish key interactions with critical residues of the α-glucosidase active site. Specifically, compound **8s** forms hydrogen bonds with ASP349, ARG212, and ASH214, while compound **8k** interacts with ASP349, ARG212, and GLU276. These interactions suggest strong binding affinity. Notably, residues such as ASP349 and GLU276 are directly involved in the enzyme’s catalytic mechanism, underscoring the functional relevance of these contacts. Additionally, the ability of compound **8r** to form hydrogen bonds with ASP408, GLU276, and GLU304, along with π-π interactions with PHE300, similar to those observed for **8s**, may contribute to its favorable inhibitory profile. In contrast, compounds demonstrating diminished activity, such as **8q**, **8i**, **10**, **3k**, and **8p**, exhibit less favorable binding modes. Specifically, compound **8i** interacts with a distinct pocket, forming hydrogen bonds with ASN241 and π-π interactions with HIE279, which may limit its ability to effectively disrupt the enzyme’s catalytic site.

#### 2.3.3. Molecular Dynamics Simulations

Molecular docking offered an initial insight into ligand orientation, providing a static snapshot of potential binding interactions within the enzyme’s active site. Nonetheless, this approach proved insufficient to fully explain the variations observed in inhibitory activity. This limitation can be attributed to the inherent constraints of docking methods, which often fail to adequately account for protein conformational flexibility and solvent effects. To overcome these limitations and obtain a more dynamic, albeit still qualitative, perspective, we complemented the docking study with a single 500 ns molecular dynamics (MD) simulation for each ligand. Specifically, MD simulations enabled us to analyze the stability of the ligand–protein complexes and to identify key molecular interactions contributing to binding affinity. To this end, a series of computational experiments were conducted as follows.

##### Analysis of Protein and Ligand Root Mean Square Deviation (RMSD)

**Figure 6:**
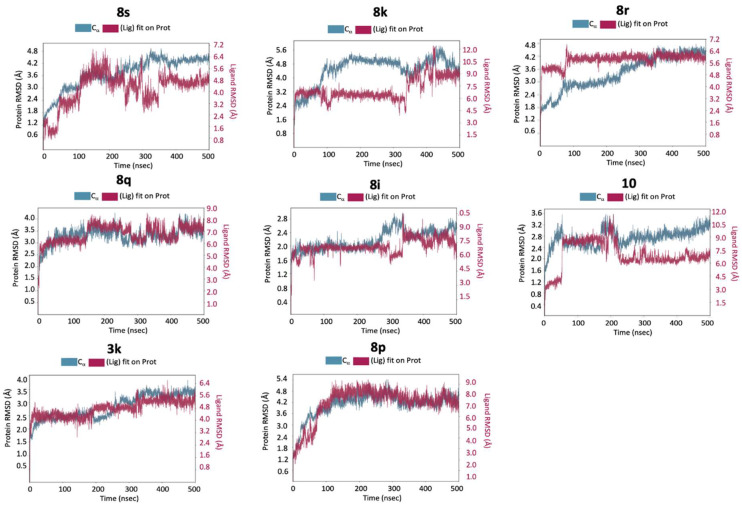
In blue: RMSD of the α-carbons of α-glucosidase in the presence of compounds **8s**, **8k**, **8r**, **8q**, **8i**, **10**, **3k** and **8p** during 500 ns of MD simulation. In red: RMSD of the ligands relative to the enzyme’s active site.

The RMSD analysis of the protein’s a-carbons showed that, for all the eight complexes, after an initial equilibration phase, fluctuations stabilize within a range of 2–3 Å (Figure 6).

**Figure 7:**
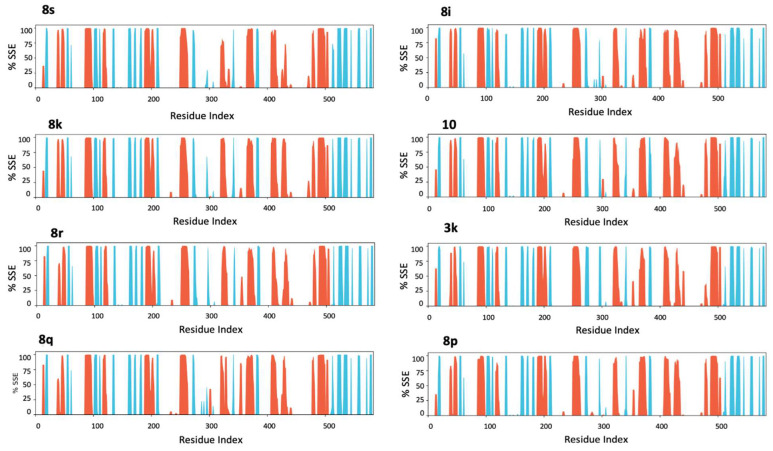
Analysis of secondary structural elements of compounds **8s**, **8k**, **8r**, **8q**, **8i**, **10**, **3k** and **8p**: α-helices in orange and β-sheets in blue.

This indicates that the overall protein structure remains stable during the simulations, without undergoing significant conformational changes. This behavior is typical of globular proteins and suggests that interaction with the different ligands does not alter the enzyme’s secondary structure, as confirmed by the analysis of secondary structural elements, which do not show significant variations (Figure 7).

##### Analysis of Protein Root Mean Square Fluctuation (RMSF)

The RMSF analysis provides information on local fluctuations of amino acid residues, allowing identification of protein regions undergoing significant dynamic variations in the presence of different ligands (Figure S7, Supplementary Material).

**Figure 8:**
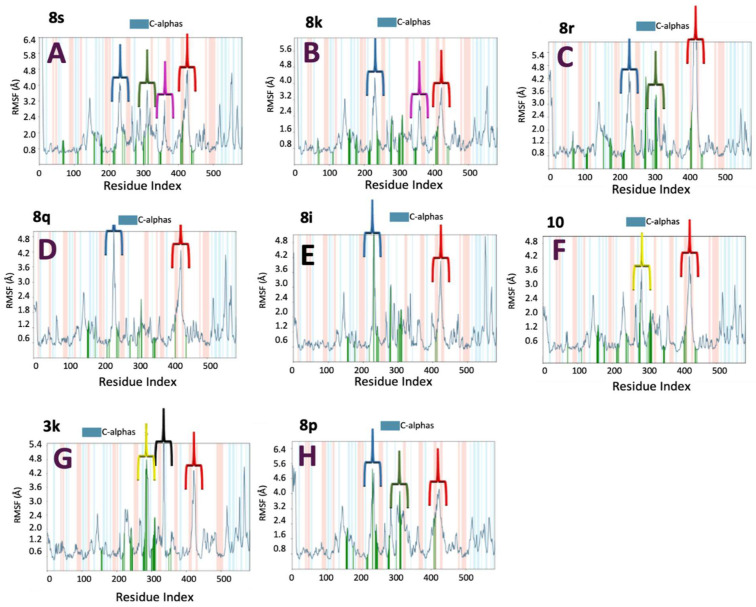
RMSF of α-glucosidase residues in the presence of different ligands of compounds **8s**, **8k**, **8r**, **8q**, **8i**, **10**, **3k** and **8p**. Regions with significant fluctuations are highlighted in different colors: blue (200–250), yellow (250–300), gray (325–350), green (300–350), purple (350–400), and red (400–450).

For compound **8s**, which exhibited the highest IC_50_ value, elevated fluctuations were observed during the MD simulation. Specifically, regions spanning residues 200–250 and 300–350 showed fluctuations of up to ~4.5 Å, while the 400–440 region exhibited even greater fluctuations, reaching ~4.8 Å. Additionally, moderate fluctuations (~3.5 Å) were detected in the 350–400 segment (Figure 8A). These flexible regions may play a role in modulating access to the active site or in stabilizing conformations essential for catalytic activity.

Compound **8k** displayed a fluctuation profile similar to that of **8s** in the 200–250 and 400–440 regions and showed fluctuations up to ~3.5 Å in the 350–400 segment. However, it exhibited reduced fluctuations in the 300–350 region (Figure 8B). This difference may reflect a diminished ability to influence critical protein dynamics associated with enzymatic inhibition. The increased flexibility in the 350–400 region, coupled with reduced motion in the 300–350 segment, could be associated with enhanced enzyme stability, consistent with the compound’s favorable IC_50_ value.

Similarly to **8s**, compound **8r** exhibited elevated fluctuations (up to ~4.5 Å) in the regions of residues 200–250 and 300–350 and fluctuations up to 6 Å in the region 400–440 (Figure 8C). However, in contrast to both **8s** and **8k**, no significant fluctuations were observed in the 350–400 region. This lack of flexibility in a region potentially involved in modulating access to the active site or stabilizing catalytically relevant conformations may be associated with the compound’s lower inhibitory activity.

Compounds **8q** and **8i** exhibit fluctuations in the region 200–250 (~4.8 Å) and in the region 400–450 but do not show significant fluctuations in the region 300–350 (Figure 8D,E). This pattern may explain their lower inhibitory activity compared to compounds **8s**, **8k**, and **8r**.

Interestingly, the less active compounds (**10**, **3k**, and **8p**) display different fluctuations. For example, **10** and **3k** present fluctuations in the region 250–300, while **8p** shows fluctuations like the more active compounds but with less magnitude (Figure 8F–H). These differences suggest that it is not just the magnitude of fluctuations that influences inhibitory activity but also the specific regions involved.

##### Analysis of Ligand–Protein Interactions

**Figure 9:**
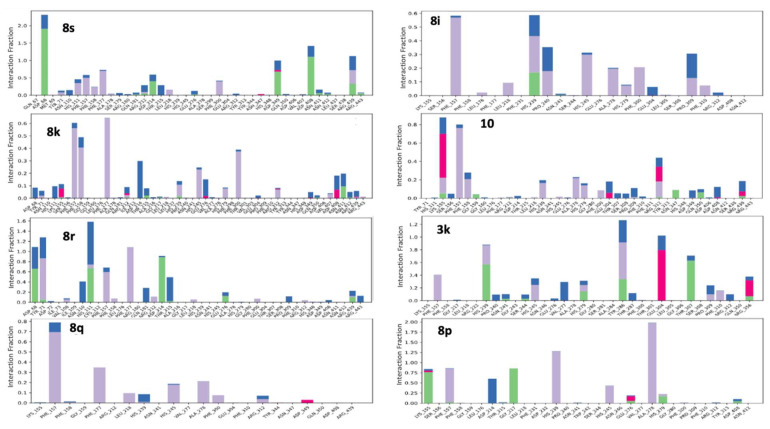
Histograms of ligand–protein contact fractions for each compound. Key interactions are highlighted, showing the percentage of time that interactions with protein amino acids are maintained during the MD simulation. In green: H-bonds, in magenta: ionic bonds, in grey: hydrophobic interactions, in blue: water bridges.

Subsequently, we investigated the contact fractions between the ligand and the protein, thereby identifying significant stabilizing interactions for each complex (Figure 9). In fact, understanding these interactions is vital for elucidating the differences in the observed inhibitory activity.

Our analysis of the most active compounds revealed that compound **8s** consistently forms stable hydrogen bonds between the hydroxyl groups on its aromatic rings and key residues, including ASP68 (95% of the time), ASP349, and ASP408 (50% of the time). Additionally, it engages in π-π and π-cation interactions with aromatic residues such as HIS111, PHE157, PHE177, and ARG439, which collectively contribute to the enhanced stability of the ligand–enzyme complex (Figure S8A, Supplementary Material).

The interactions of compound **8k** include π-cation interactions involving its nitro groups and aromatic residues such as PHE157, PHE158, and PHE177, occurring approximately 70% of the simulation time. Additionally, π-π interactions with PHE300 were observed with ~40% of the time (Figure S8B, Supplementary Material). Although stable hydrogen bonds with catalytic residues were not detected, potentially contributing to its slightly lower inhibition compared to compound **8s**, compound **8k** maintained persistent hydrophobic interactions throughout the simulation. This sustained interaction network suggests a robust stability of the molecular scaffold, which may underlie its interesting IC_50_ value.

Compound **8r** establishes hydrogen bonds with HIS111 via water-mediated bridges and interacts with ASP214 through the nitrogen atom of its heterocyclic ring, occurring 83% of the simulation time. Additionally, it engages in aromatic interactions with TYR71, PHE157, and PHE177, each occurring for more than 40% of the time (Figure S8C, Supplementary Material). These interactions contribute to the overall stability of the complex and may partially explain its moderate inhibitory activity.

Afterward, compounds that demonstrated diminished inhibitory activity were examined. Compounds **8q** and **8i** mainly participate in aromatic π-π interactions with PHE157 and PHE177, but they lack significant hydrogen bonding with important residues, which ultimately lowers their overall affinity (Figure S9A,B, Supplementary Material). Compounds **10** and **3k** exhibit unstable ionic and aromatic interactions and do not effectively interact with the enzyme’s catalytic residues (Figure S9C,D, Supplementary Material). Compound **8p** shows hydrogen bonding with LYS155 and transient aromatic interactions with PHE157 and HIS239. A distinctive aspect of this compound is its intramolecular interactions, which hinder its ability to interact with the protein. This limitation may explain its low inhibitory activity, despite the presence of potential amino acid variations like those found in the most effective compounds (Figure S9E, Supplementary Material).

##### Correlation Between Molecular Interactions and Inhibitory Activity

A comprehensive analysis of ligand–-protein interactions in conjunction with protein fluctuations revealed that the most efficacious compounds can engage in multiple stable interactions with the catalytic and structural residues of α-glucosidase. Specifically, the formation of hydrogen bonds with residues, including ASP68, ASP214, ASP349, and ASP408, is critical for the successful inhibition of the enzyme. In fact, these residues are known to be involved in the catalytic mechanism of α-glucosidase. Additionally, interactions involving aromatic residues such as PHE157, PHE177, and HIS111 play a significant role in enhancing the stability of the complex and may affect the dynamics of critical regions within the enzyme. The occurrence of π-π and π-cation interactions not only strengthens binding affinity but may also trigger conformational alterations that promote enzymatic inhibition. Conversely, the compounds that demonstrate reduced activity are characterized by a lack of these crucial interactions, or, in the case of compound **8p**, they establish intramolecular interactions that constrain their ability to interact productively with the protein or restrict access to the catalytic residues.

## 3. Materials and Methods

### 3.1. Chemistry

#### 3.1.1. Materials and Methods

All materials were sourced from commercial suppliers and utilized without additional purification unless specified otherwise. The progress of the reactions was followed through thin-layer chromatography employing Merck silica gel 60 pre-coated plates with fluorescent indicator F254 (0.25 mm) (Merck, Sigma Aldrich, Milan, Italy). The reaction products were achieved through column flash chromatography using silica gel with a mesh size of 0.040–0.063 (Merck, Sigma Aldrich, Milan, Italy) or by filtration/precipitation and recrystallization from ethyl acetate/hexane 2/1, 1/1 or DCM. The purity was assessed using an UHPLC-QTOF/MS coupled with an Agilent 1290 Infinity II LC system (Agilent, Technologies, Palo Alto, CA, USA) when required. ^1^H and ^13^C NMR spectra were obtained on a Bruker Avance III™ HD 600 MHz spectrometer (Bruker, Billerica, MA, USA) operating at 600 and 151 MHz frequencies, respectively. High-resolution mass spectra were recorded on a high-resolution LTQ Orbitrap Elite™ mass spectrometer (Thermo Fisher Scientific, Waltham, MA, USA). The solutions were infused into the ESI source at a flow rate of 5.00 μL/min. Spectra were recorded with a resolution of 240,000 (FWHM). Instrument conditions were as follows: spray voltage 4000 and 5000 V for positive and negative ionization mode, respectively, capillary temperature 275 °C, sheath gas 12 (arbitrary units), auxiliary gas 3 (arbitrary units), sweep gas 0 (arbitrary units), and probe heater temperature 50 °C.

#### 3.1.2. General Synthetic Procedure

In a round-bottom flask, a suspension of *o*-phenylenediamine (1.5 mmol), aldehyde (3.3 mmol) and the appropriate amount of ASA or SA in distilled water (5 mL) was stirred at room temperature for the indicated period. After the reaction, monitored by thin-layer chromatography (TLC) (Merck Sigma Aldrich, Milan, Italy), AcOEt (4 mL) and Na_2_CO_3_ were added to the reaction and stirred for 15 min. After separating the salt by filtration, the crude mixture was extracted with AcOEt (4 × 30 mL) and the combined organic phase was dried on Na_2_SO_4_ and concentrated under vacuum. Subsequently, a 1:1 mixture of ethyl acetate and hexane was added to the crude product and stirred for approximately one hour. The resulting suspension was filtered, and the solid was crystallized from the ethyl acetate/hexane mixture or DCM to yield 2-aryl-1-arylmethyl-1H-benzimidazole as pure product. In certain instances, the application of chromatography proved essential, especially for reactions resulting in both 2- and 1,2-disubstituted derivatives, as well as for compounds like **8r** and **8s**.

#### 3.1.3. Characterization Data of All the Tested Compounds

**1-Benzyl-2-phenyl-1*H*-benzo[*d*]imidazole 3a.** White solid. M.p.130–131 °C. NMR spectra are consistent with those previously reported [38]. ^1^H NMR (600 MHz, CDCl_3_): δ 7.85 (d, J = 8.0 Hz, 1H), 7.59 (dd, J = 8.2, 2.0 Hz, 2H), 7.30 (d, J = 7.2 Hz, 1H), 7.29–7.17 (m, 6H), 7.13 (d, J = 7.5 Hz, 2H), 7.00 (d, J = 7.6 Hz, 2H), 5.41 (s, 2H) ppm. ^13^C NMR (151 MHz, CDCl_3_): δ 154.50, 143.36, 140.16, 137.58, 136.26, 133.62, 129.84, 129.57, 129.32, 127.37, 126.05, 122.96, 122.67, 119.99, 110.64, 48.35ppm. HRMS(ESI): m/z cal. for C_20_H_16_N_2_: 284.1313, found [M + H]^+^ 285.1323.

**1-((1*H*-Indol-3-yl)methyl)-2-(1*H*-indol-3-yl)-1*H*-benzo[*d*]imidazole 3b.** Pale yellow solid. M.p. 233–235 °C. NMR spectra are consistent with those previously reported [39]. ^1^H NMR (600 MHz, DMSO): δ 11.66 (s, 1H), 10.98 (d, *J* = 2.6 Hz, 1H), 8.33 (dt, *J* = 7.9, 1.1 Hz, 1H), 7.86 (s, 1H), 7.70–7.65 (m, 1H), 7.57–7.53 (m, 1H), 7.49 (dt, *J* = 8.0, 1.0 Hz, 1H), 7.32 (dd, *J* = 8.1, 1.0 Hz, 1H), 7.25 (t, *J* = 8.0 Hz, 1H), 7.24–7.20 (m, 1H), 7.17 (dtd, *J* = 16.4, 7.5, 1.3 Hz, 3H), 7.06–7.01 (m, 2H), 6.84 (ddd, *J* = 8.0, 7.0, 1.0 Hz, 1H), 5.84 (s, 2H) ppm. ^13^C NMR (600 MHz, DMSO): δ 149.64, 143.35, 136.34, 136.06, 135.62, 126.51, 126.36, 125.55, 123.52, 122.32, 121.42, 121.41, 121.39, 121.33, 120.26, 118.82, 118.37, 118.21, 111.80, 111.70, 110.45, 110.40, 105.14, 40.71 ppm. HRMS(ESI): m/z cal. for C_24_H_18_N_4_: 362.1531, found [M + H]^+^ 363.1533.

**2-(Benzo[*d*][1,3]dioxol-5-yl)-1-(benzo[*d*][1,3]dioxol-5-ylmethyl)-1*H*-benzo[*d*]imidazole 3c.** Beige solid. M.p. 175–177 °C. NMR spectra are consistent with those previously reported [40]. ^1^H NMR (600 MHz, CDCl_3_): δ 7.85 (dt, *J* = 8.0, 1.0 Hz, 1H), 7.62 (d, *J* = 1.3 Hz, 1H), 7.33 (ddd, *J* = 8.1, 5.9, 2.5 Hz, 1H), 7.30–7.24 (m, 2H), 7.19–7.14 (m, 2H), 6.85 (dd, *J* = 7.5, 0.9 Hz, 1H), 6.61–6.56 (m, 2H), 6.01 (s, 2H), 5.96 (s, 2H), 5.37 (s, 2H) ppm.^13^C NMR (151 MHz, CDCl_3_): δ 153.91, 149.27, 148.52, 143.03, 136.07, 123.72, 123.14, 122.86, 119.88, 119.40, 110.60, 109.73, 108.80, 108.71, 106.66, 101.68, 101.59, 101.40, 48.29.ppm. HRMS(ESI): m/z cal. for C_22_H_16_N_2_O_4_: 372.1183, found [M + H]^+^ 373.1187.

**1-(2-Nitrobenzyl)-2-(2-nitrophenyl)-1*H*-benzo[*d*]imidazole 3d.** Pale yellow solid. M.p. 172–174 °C. NMR spectra are consistent with those previously reported [41]. ^1^H NMR (600 MHz, CD_3_CN): δ 8.10 (ddt, *J* = 7.6, 4.1, 2.3 Hz, 2H), 7.77–7.74 (m, 1H), 7.74–7.70 (m, 2H), 7.59–7.55 (m, 1H), 7.54–7.47 (m, 2H), 7.35–7.29 (m, 3H), 6.88 (ddt, J = 7.7, 2.3, 1.0 Hz, 1H), 5.74 (s, 1H) ppm. ^13^C NMR (151 MHz, CD_3_CN): δ 150.94, 148.44, 144.12, 136.31, 135.15, 134.54, 132.90, 132.63, 132.49, 129.91, 129.90, 129.37, 126.33, 125.99, 124.54, 123.81, 120.76, 118,30, 111.93, 46.52 ppm. HRMS(ESI): m/z cal. for C_20_H_14_N_4_O_4_ [M + H]^+^: 374.1088, found 375.1086.

**1-(2-Bromobenzyl)-2-(2-bromophenyl)-1*H*-benzo[*d*]imidazole 3e.** Pale yellow solid. M.p. 148–150 °C. NMR spectra are consistent with those previously reported [19]. ^1^H NMR (600 MHz, CDCl_3_): δ 7.89 (d, *J* = 8.0 Hz, 1H), 7.70 (dq, *J* = 5.7, 2.9, 2.0 Hz, 1H), 7.52 (dt, *J* = 6.5, 1.9 Hz, 1H), 7.42–7.37 (m, 1H), 7.37–7.31 (m, 3H), 7.30–7.24 (m, 1H), 7.20 (d, *J* = 8.1 Hz, 1H), 7.12–7.06 (m, 2H), 6.60 (dt, *J* = 6.7, 1.9 Hz, 1H), 5.32 (s, 2H).ppm. ^13^C NMR (151 MHz, CDCl_3_): δ 152.71, 143.09, 135.02, 134.82, 133.20, 133.01, 132.25, 131.88, 131.68, 129.37, 127.97, 127.89, 127.58, 124.08, 123.56, 122.90, 122.31, 120.58, 110.67, 48.34ppm. HRMS(ESI): m/z cal. for C_20_H_14_Br_2_N_2_: 442.9597, found [M + H]^+^ 4413.9597.

**4-(1-(4-Hydroxybenzyl)-1*H*-benzo[*d*]imidazol-2-yl)phenol 3f.** Beige solid. M.p. 223–225 °C. NMR spectra are consistent with those previously reported [39]. ^1^H NMR (600 MHz, DMSO): δ 7.57–7.52 (m, 2H), 7.43–7.38 (m, 1H), 7.23–7.11 (m, 2H), 6.92 (dd, *J* = 8.6, 6.8 Hz, 2H), 6.85–6.80 (m, 2H), 6.70–6.65 (m, 2H), 5.41 (s, 2H) ppm. ^13^C NMR (151 MHz, DMSO): δ 159.25, 156.84, 153.67, 142.74, 135.86, 131.29, 131.12, 130.51, 127.44, 124.72, 122.47, 122.03, 121.79, 120.48, 118.78, 115.68, 115.61, 115.50, 110.91, 59.73 ppm. HRMS(ESI): m/z cal. for C_20_H_16_N_2_O_2_: 316.1285, found [M + H]^+^ 317.2584.

**1-(4-Methylbenzyl)-2-(*p*-tolyl)-1*H*-benzo[*d*]imidazole 3g.** Pale brown solid. M.p. 129–131 °C. NMR spectra are consistent with those previously reported [42]. ^1^H NMR (600 MHz, CDCl_3_): δ 7.88–7.83 (m, 1H), 7.61–7.57 (m, 2H), 7.29 (ddd, *J* = 8.2, 6.5, 1.8 Hz, 1H), 7.25 (s, 2H), 7.24–7.18 (m, 2H), 7.13 (d, *J* = 7.9 Hz, 2H), 7.00 (d, *J* = 7.9 Hz, 2H), 5.41 (s, 2H), 2.41 (s, 3H), 2.34 (s, 3H) ppm. ^13^C NMR (151 MHz, CDCl_3_): δ 154.53, 143.22, 138.94, 136.64, 136.28, 130.81, 130.40, 130.06, 129.06, 128.66, 128.64, 126.81, 126.19, 123.25, 123.10, 122.75, 120.03, 110.68, 48.58, 21.58, 21.48 ppm. HRMS(ESI): m/z cal. for C_22_H_20_N_2_: 312.1699, found [M + H]^+^ 313.1700.

**2-(Thiophen-3-yl)-1-(thiophen-3-ylmethyl)-1*H*-benzo[*d*]imidazole 3h.** Yellow solid. M.p. 190–192 °C. Pale yellow solid. M.p. 148-150 °C. NMR spectra are consistent with those previously reported [43]. ^1^H NMR (600 MHz, CDCl_3_): δ 7.83 (dt, *J* = 7.9, 1.0 Hz, 1H), 7.64 (dd, *J* = 2.9, 1.3 Hz, 1H), 7.52 (dd, *J* = 5.0, 1.3 Hz, 1H), 7.42 (dd, *J* = 5.1, 2.9 Hz, 1H), 7.34 (dd, *J* = 4.9, 3.1 Hz, 1H), 7.32–7.27 (m, 2H), 7.25 (ddd, *J* = 8.0, 7.0, 1.2 Hz, 1H), 6.95–6.90 (m, 2H), 5.47 (d, *J* = 1.2 Hz, 2H) ppm. ^13^C NMR (151 MHz, CDCl_3_): δ 149.48, 143.08, 137.61, 135.89, 131.00, 128.29, 127.55, 126.65, 126.47, 125.73, 123.19, 122.84, 121.70, 119.95, 110.07, 44.74 ppm. HRMS(ESI): m/z cal. for C_16_H_12_N_2_S_2_: 296.0515, found [M + H]^+^ 297.0607.

**1-(2-Methoxybenzyl)-2-(2-methoxyphenyl)-1*H*-benzo[*d*]imidazole 3i.** Beige solid. M.p. 131–133 °C. NMR spectra are consistent with those previously reported [44]. ^1^H NMR (600 MHz, CDCl_3_): δ 7.84 (d, *J* = 8.0 Hz, 1H), 7.55–7.50 (m, 1H), 7.45 (t, *J* = 8.1 Hz, 1H), 7.23–7.16 (m, 4H), 7.07–7.02 (m, 1H), 6.95 (d, *J* = 8.4 Hz, 1H), 6.83 (d, *J* = 8.2 Hz, 1H), 6.76 (t, *J* = 7.6 Hz, 1H), 6.69 (d, *J* = 7.6 Hz, 1H), 5.23 (s, 2H), 3.78 (s, 3H), 3.59 (s, 3H). ppm. ^13^C NMR (151 MHz, CDCl_3_): δ 157.77, 156.67, 132.55, 131.53, 128.52, 127.92, 124.74, 122.59, 122.08, 120.94, 120.55, 119.97, 110.98, 110.89, 110.08, 55.36, 55.29, 43.66 ppm. HRMS(ESI): m/z calc. for C_22_H_20_N_2_O_2_: 344.1598, found [M + H]^+^ 345.1598.

**4-(1-(4-Hydroxy-3-methoxybenzyl)-1*H*-benzo[*d*]imidazol-2-yl)-2-methoxyphenol 3j.** Pale yellow solid. M.p. 186–188 °C. NMR spectra are consistent with those previously reported [39]. ^1^H NMR (600 MHz, DMSO): δ 9.60 (s, 1H), 9.00 (s, 1H), 7.68–7.62 (m, 1H), 7.50–7.44 (m, 1H), 7.24 (d, *J* = 2.0 Hz, 1H), 7.23–7.20 (m, 2H), 7.17 (dd, *J* = 8.1, 2.0 Hz, 1H), 6.91 (d, *J* = 8.2 Hz, 1H), 6.67 (d, *J* = 2.0 Hz, 1H), 6.64 (d, *J* = 8.1 Hz, 1H), 6.36 (dd, *J* = 8.1, 2.0 Hz, 1H), 5.43 (s, 2H), 3.71 (s, 3H), 3.62 (s, 3H) ppm. ^13^C NMR (151 MHz, DMSO): δ 153.66, 148.32, 147.81, 147.72, 145.94, 142.60, 136.06, 127.95, 122.38, 122.16, 122.10, 121.15, 118.91, 118.64, 115.80, 115.69, 115.65, 113.12, 111.00, 110.82,, 55.61, 55.58, 47.43.ppm. HRMS(ESI): m/z calc. for C_22_H_20_N_2_O_4_: 376.1496, found [M + H]^+^ 377.1523.

**1-(4-Chloro-3-nitrobenzyl)-2-(4-chloro-3-nitrophenyl)-1*H*-benzo[*d*]imidazole 3k.** Pale yellow solid. M.p. 192–194 °C. NMR spectra are consistent with those previously reported [30]. ^1^H NMR (600 MHz, DMSO): δ 8.39 (s, 1H), 8.03–7.98 (m, 1H), 7.93 (dd, *J* = 8.4, 2.1 Hz, 1H), 7.84 (s, 1H), 7.79 (dq, *J* = 5.7, 3.0, 2.4 Hz, 1H), 7.68 (dd, *J* = 8.5, 2.0 Hz, 1H), 7.58 (dt, *J* = 6.0, 2.4 Hz, 1H), 7.33 (dq, *J* = 5.6, 3.1, 2.4 Hz, 2H), 7.25 (dd, *J* = 8.4, 2.5 Hz, 1H), 5.74 (s, 2H)) ppm. ^13^C NMR (151 MHz, DMSO): δ 150.03, 147.55, 147.52, 142.48, 137.82, 135.87, 134.04, 132.28, 132.10, 131.59, 130.04, 126.55, 126.19, 124.05, 123.81, 123.68, 122.91, 119.73, 111.21, 46.40 ppm. HRMS(ESI): m/z calc. for C_20_H_12_ Cl_2_N_4_O_4_: 442.0308, found [M + H]^+^ 443.0308.

**2-(Furan-3-yl)-1-(furan-3-ylmethyl)-1*H*-benzo[*d*]imidazole 3l.** Beige solid. M.p. 190–192 °C. NMR spectra are consistent with those previously reported [30].^1^H NMR (600 MHz, CDCl_3_): δ 7.78 (dd, *J* = 6.5, 3.6 Hz, 1H), 7.65 (s, 1H), 7.50 (dd, *J* = 6.2, 3.4 Hz, 1H), 7.35–7.24 (m, 4H), 7.22 (t, *J* = 2.6 Hz, 1H), 6.61 (q, *J* = 1.9 Hz, 1H), 6.28 (q, *J* = 2.3 Hz, 1H), 6.24 (t, *J* = 2.7 Hz, 1H), 5.65 (d, *J* = 2.0 Hz, 2H) ppm. ^13^C NMR (151 MHz, CDCl_3_): δ 149.74, 145.54, 144.12, 144.07, 143.10, 142.81, 135.64, 123.40, 123.08, 119.96, 113.08, 112.21, 110.68, 110.13, 108.50, 41.84 ppm. HRMS(ESI): m/z calc. for C_16_H_12_N_2_O_2_: 264.0972, found [M + H]^+^ 265.0980.

**2-(Pyridin-3-yl)-1-(pyridin-3-ylmethyl)-1*H*-benzo[*d*]imidazole 3m.** Beige solid. M.p. 129–130 °C. NMR spectra are consistent with those previously reported [41]. ^1^H NMR (600 MHz, CDCl_3_): δ 8.86 (d, *J* = 2.2 Hz, 1H), 8.68 (dd, *J* = 4.9, 1.7 Hz, 1H), 8.51 (dd, *J* = 4.8, 1.6 Hz, 1H), 8.41 (d, *J* = 2.4 Hz, 1H), 7.96 (dt, *J* = 7.9, 2.0 Hz, 1H), 7.85 (d, *J* = 8.1 Hz, 1H), 7.38 (ddd, *J* = 7.9, 4.9, 0.9 Hz, 1H), 7.31 (ddd, *J* = 8.2, 7.1, 1.2 Hz, 1H), 7.28–7.23 (m, 2H), 7.23–7.17 (m, 2H), 5.45 (s, 2H).ppm. ^13^C NMR (151 MHz, CDCl_3_): δ 150.91, 150.77, 149.47, 149.41, 147.64, 143.11, 135.66, 133.64, 131.52, 126.18, 123.91, 123.88, 123.65, 123.28, 120.34, 110.24, 46.05 ppm. HRMS(ESI): m/z calc. for C_18_H_14_N_4_: 286.1291, found [M + H]^+^ 287.1357.

**1-((1*H*-Imidazol-4-yl)methyl)-2-(1*H*-imidazol-4-yl)-1*H*-benzo[*d*]imidazole 3n.** Pale yellow solid. M.p. 248–250 °C. ^1^H NMR (600 MHz, DMSO): δ 7.89 (dd, *J* = 11.3, 1.2 Hz, 2H), δ 7.89 (dd, J = 11.3, 1.2 Hz, 2H), 7.67–7.61 (m, 1H), 7.57–7.54 (m, 1H), 7.52 (d, J = 1.2 Hz, 1H), 7.19–7.12 (m, 2H), 6.94 (s, 1H), 5.87 (s, 2H) ppm.^13^C NMR (151 MHz, DMSO): δ 147.17, 142.76, 136.58, 135.53, 135.06, 121.56, 121.38, 121.25, 118.08, 110.91, 41.21 ppm. HRMS(ESI): m/z calc. for C_14_H_12_N_6_: 264.1196, found [M + H]^+^ 265.1224.

**1-(3,4-Dimethoxybenzyl)-2-(3,4-dimethoxyphenyl)-1*H*-benzo[*d*]imidazole 3o.** Brown solid. M.p. 131–133 °C. NMR spectra are consistent with those previously reported [41]. ^1^H NMR (600 MHz, CDCl_3_): δ 7.86 (dt, *J* = 8.0, 1.0 Hz, 1H), 7.31 (ddd, *J* = 8.1, 5.8, 2.5 Hz, 1H), 7.28 (d, *J* = 2.0 Hz, 1H), 7.26–7.22 (m, 3H), 6.92 (d, *J* = 8.3 Hz, 1H), 6.81 (d, *J* = 8.1 Hz, 1H), 6.68–6.62 (m, 2H), 5.40 (s, 2H), 3.92 (s, 3H), 3.86 (s, 3H), 3.77 (d, *J* = 8.5 Hz, 6H) ppm. ^13^C NMR (151 MHz, CDCl_3_): δ 154.14, 150.50, 149.52, 149.10, 148.58, 143.04, 136.30, 129.10, 122.93, 122.67, 122.55, 121.86, 119.71, 118.13, 112.35, 111.57, 111.03, 110.30, 109.09, 55.97, 55.95, 55.91, 55.82, 48.14 ppm. HRMS(ESI): m/z calc. for C_24_H_24_N_2_O_4_: 404.1809, found [M + H]^+^ 405.1925.

**2-(Pyridin-3-yl)-1*H*-benzo[*d*]imidazole 4m.** Pale yellow solid. M.p. 245–247 °C. NMR spectra are consistent with those previously reported [44]. ^1^H NMR (600 MHz, DMSO): δ 13.10 (s, 1H), 9.35 (d, *J* = 2.2 Hz, 1H), 8.68 (dd, *J* = 4.8, 1.6 Hz, 1H), 8.50 (dt, *J* = 8.0, 2.0 Hz, 1H), 7.71 (d, *J* = 7.9 Hz, 1H), 7.63–7.55 (m, 2H), 7.29–7.19 (m, 2H) ppm. ^13^C NMR (151 MHz, DMSO): δ 150.49, 148.85, 147.51, 133.74, 126.15, 124.00, 122.97, 121.96, 119.07, 111.52 ppm. HRMS(ESI): m/z calc. for C_12_H_9_N_3_: 195.0869, found [M + H]^+^ 196.0923.

**1-Benzyl-5-nitro-2-phenyl-1H-benzo[d]imidazole 5a.** Pale yellow solid. M.p. 149–151 °C. NMR spectra are consistent with those previously reported [44]. ^1^H NMR (600 MHz, CDCl_3_): δ 8.24 (dd, *J* = 8.9, 2.2 Hz, 1H), 8.17 (d, *J* = 2.2 Hz, 1H), 7.90 (d, *J* = 8.9 Hz, 1H), 7.75–7.69 (m, 2H), 7.58–7.52 (m, 1H), 7.52–7.47 (m, 2H), 7.39–7.30 (m, 3H), 7.11–7.05 (m, 2H), 5.55 (s, 2H) ppm. ^13^C NMR (151 MHz, CDCl_3_): δ 158.97, 147.74, 143.84, 135.56, 135.29, 131.04, 129.41, 129.18, 129.08, 128.49, 126.00, 120.13, 118.83, 107.57, 48.94 ppm. HRMS(ESI): m/z calc. for C_20_H_15_N_3_O_2_: 329.1164, found [M + H]^+^ 330.1237.

**5-Nitro-2-phenyl-1*H*-benzo[*d*]imidazole 6a.** Beige solid. M.p. 205–207 °C. NMR spectra are consistent with those previously reported [43]. ^1^H NMR (600 MHz, DMSO): δ 13.60 (s, 1H), 8.48 (s, 1H), 8.22 (dt, *J* = 8.0, 1.3 Hz, 2H), 8.14 (dt, *J* = 8.7, 1.7 Hz, 1H), 7.78 (d, *J* = 8.8 Hz, 1H), 7.64–7.55 (m, 3H) ppm. ^13^C NMR (151 MHz, DMSO): δ 142.72, 130.97, 129.16, 129.02, 126.97, 117.99 ppm. HRMS(ESI): m/z calc. for C_13_H_9_N_3_O_2_: 239.0768, found [M + H]^+^ 240.0821.

**2-(2-Methoxyphenyl)-5-nitro-1*H*-benzo[*d*]imidazole 6i.** Light brown. M.p. 205–207 °C. NMR spectra are consistent with those previously reported [45]. ^1^H NMR (600 MHz, DMSO): δ 12.66 (s, 1H), 8.53 (d, *J* = 2.3 Hz, 1H), 8.37 (d, *J* = 7.9 Hz, 1H), 8.15–8.10 (m, 1H), 7.80 (d, *J* = 8.8 Hz, 1H), 7.56 (ddd, *J* = 8.7, 7.2, 1.8 Hz, 1H), 7.30 (d, *J* = 8.4 Hz, 1H), 7.16 (td, *J* = 7.5, 1.0 Hz, 1H), 4.06 (s, 3H) ppm. ^13^C NMR (151 MHz, DMSO): δ 157.32, 157.08, 142.23, 132.69, 132.44, 130.12, 121.06, 118.47, 117.85, 117.46, 114.44, 112.35, 108.69, 55.95 ppm. HRMS(ESI): m/z calc. for C_14_H_11_N_3_O_3_: 269.0800, found [M + H]^+^ 270.0767.

**2-(4-Chloro-3-nitrobenzylidene)amino)-5-nitroaniline 7.** Light orange solid. M.p. 218–220 °C. ^1^H NMR (600 MHz, DMSO): δ 8.97 (s, 1H), 8.82 (d, *J* = 1.9 Hz, 1H), 8.34 (dd, *J* = 8.4, 1.9 Hz, 1H), 8.14 (d, *J* = 2.6 Hz, 1H), 7.98–7.92 (m, 2H), 6.96 (s, 1H), 6.79 (d, *J* = 9.1 Hz, 1H) ppm. ^13^C NMR (151 MHz, DMSO): δ 156.28, 151.48, 148.18, 136.54, 135.82, 133.84, 132.39, 132.35, 131.96, 126.86, 125.00, 124.75, 113.31 ppm. HRMS(ESI): m/z calc. for C_13_H_9_ClN_4_O_4_: 320.0385, found [M + H]^+^ 321.0385.

**1-Benzyl-4-methyl-2-phenyl-1*H*-benzo[*d*]imidazole 8a.** White solid. M.p. 169–171 °C. NMR spectra are consistent with those previously reported [46]. ^1^H NMR (600 MHz, CDCl_3_): δ 7.72–7.65 (m, 2H), 7.49–7.42 (m, 3H), 7.35–7.26 (m, 3H), 7.18–7.04 (m, 5H), 5.41 (s, 2H), 2.78 (s, 3H) ppm. ^13^C NMR (151 MHz, CDCl_3_): δ 153.57, 142.62, 136.65, 135.70, 130.48, 130.17, 129.83, 129.51, 129.07, 128.79, 127.77, 126.12, 123.08, 123.00, 108.09, 48.44, 16.94 ppm. HRMS(ESI): m/z calc. for C_21_H_18_N_2_: 298.1470, found [M + H]^+^ 299.1465.

**1-(2-Methoxybenzyl)-2-(2-methoxyphenyl)-4-methyl-1*H*-benzo[*d*]imidazole 8i.** Light brown solid. M.p. 188–190 °C. ^1^H NMR (600 MHz, CDCl_3_): δ 7.54 (dd, *J* = 7.5, 1.8 Hz, 1H), 7.43 (ddd, *J* = 8.4, 7.4, 1.8 Hz, 1H), 7.18 (ddd, *J* = 8.2, 7.4, 1.7 Hz, 1H), 7.14–7.01 (m, 4H), 6.94 (dd, *J* = 8.4, 1.0 Hz, 1H), 6.82 (dd, *J* = 8.3, 1.1 Hz, 1H), 6.76 (td, *J* = 7.5, 1.1 Hz, 1H), 6.71–6.67 (m, 1H), 5.22 (s, 2H), 3.78 (s, 3H), 3.58 (s, 3H), 2.76 (s, 3H) ppm. ^13^C NMR (151 MHz, CDCl_3_): δ 150.49, 149.51, 149.09, 148.57, 136.29, 129.09, 122.92, 122.66, 121.85, 119.70, 118.12, 111.56, 111.02, 110.29, 109.89, 55.96, 55.94, 48.13, 21.03 ppm. HRMS(ESI): m/z calc. for C_23_H_22_N_2_O_2_: 358.1754, found [M + H]^+^ 359.1919.

**1-(4-Chloro-3-nitrobenzyl)-2-(4-chloro-3-nitrophenyl)-4-methyl-1*H*-benzo[*d*]imidazole 8k.** Pale yellow solid. M.p. 166–168 °C. NMR spectra are consistent with those previously reported [30]. ^1^H NMR (600 MHz, CDCl_3_): δ 8.39 (d, *J* = 1.9 Hz, 1H), 7.94 (d, *J* = 8.3 Hz, 1H), 7.79 (d, *J* = 8.2 Hz, 1H), 7.73 (d, *J* = 8.4 Hz, 1H), 7.57 (d, *J* = 8.3 Hz, 1H), 7.33 (t, *J* = 7.8 Hz, 1H), 7.28 (d, *J* = 8.3 Hz, 2H), 7.20 (dd, *J* = 8.4, 2.2 Hz, 1H), 7.13 (d, *J* = 8.1 Hz, 1H), 5.56 (s, 2H), 2.77 (s, 3H) ppm. ^13^C NMR (151 MHz, CDCl_3_): δ 154.13, 149.09, 148.57, 143.03, 129.09, 122.92, 122.66, 122.54, 121.85, 118.12, 111.56, 111.02, 110.29, 109.08, 48.13, 21.03 ppm. HRMS: m/z calc. for C_21_H_14_Cl_2_N_4_O_4_: 456.0470, found [M + H]^+^ 457.0437.

**4-(1-(4-Hydroxy-3-nitrobenzyl)-4-methyl-1*H*-benzo[*d*]imidazol-2-yl)-2-nitrophenol 8p.** Yellow solid. M.p. 195–197 °C. NMR spectra are consistent with those previously reported [30]. ^1^H NMR (600 MHz, CDCl_3_): δ 8.41 (s, 1H), 8.01 (dd, *J* = 8.7, 2.2 Hz, 1H), 7.94–7.91 (m, 1H), 7.34 (d, *J* = 4.6 Hz, 1H), 7.28 (d, *J* = 7.6 Hz, 1H), 7.22 (dd, *J* = 6.3, 1.0 Hz, 1H), 7.14 (d, *J* = 8.0 Hz, 2H), 7.08 (dt, *J* = 9.4, 0.9 Hz, 1H), 5.45 (s, 2H), 2.79 (s, 3H) ppm. ^13^C NMR (151 MHz, CDCl_3_): δ 157.77, 132.55, 131.53, 128.52, 127.92, 122.59, 122.08, 120.94, 120.55, 119.97, 110.98, 110.89, 110.08, 43.66, 29.85 ppm. HRMS(ESI): m/z calc. for C_21_H_16_N_4_O_6_: 420.1070, found [M + H]^+^ 421.1059.

**1-(3,4-Dibromobenzyl)-2-(3,4-dibromophenyl)-4-methyl-1*H*-benzo[*d*]imidazole 8q.** Pale yellow solid. M.p. 152–154 °C. ^1^H NMR (600 MHz, CDCl_3_): δ 7.95 (d, J = 2.1 Hz, 1H), 7.70 (d, J = 8.3 Hz, 1H), 7.56 (d, J = 8.3 Hz, 1H), 7.38 (d, J = 2.1 Hz, 1H), 7.35 (dd, J = 8.2, 2.1 Hz, 1H), 7.21 (t, J = 7.6 Hz, 1H), 7.15 (d, J = 7.2 Hz, 1H), 7.04 (d, J = 8.0 Hz, 1H), 6.82 (dd, J = 8.3, 2.1 Hz, 1H), 5.31 (s, 2H), 2.74 (s, 3H) ppm. ^13^C NMR (151 MHz, CDCl_3_): δ 151.42, 143.06, 136.95, 135.89, 134.49, 134.30, 134.06, 131.20, 130.36, 128.56, 127.17, 126.00, 125.90, 125.70, 124.64, 124.03, 123.44, 120.51, 110.16, 47.39, 22.28 ppm. HRMS(ESI): m/z calc. for C_21_H_14_Br_4_N_2_ [M + H]^+^: 609.7963, found 610.7980.

**2-(1-(2,5-Dihydroxybenzyl)-4-methyl-1*H*-benzo[*d*]imidazol-2-yl)benzene-1,4-diol 8r.** Beige solid. M.p. 195–198 °C. ^1^HNMR (600 MHz, DMSO): δ 10.56 (s, 1H), 9.11 (s, 1H), 8.99 (s, 1H), 8.52 (s, 1H), 7.14–7.08 (m, 2H), 7.08–7.04 (m, 1H), 6.88–6.83 (m, 2H), 6.79 (dd, *J* = 8.6, 2.9 Hz, 1H), 6.65 (d, *J* = 8.6 Hz, 1H), 6.43 (dd, *J* = 8.6, 2.9 Hz, 1H), 5.79 (d, *J* = 2.9 Hz, 1H), 5.32 (s, 2H), 2.58 (s, 3H) ppm. ^13^C NMR (151 MHz, DMSO): δ 148.28, 141.86, 140.30, 137.02, 133.03, 131.10, 127.12, 122.85, 112.52, 109.66, 46.26, 24.62 ppm. HRMS: m/z calc. for C_21_H_18_N_2_O_4_: 362.1339, found [M − H]^+^ 361.1195.

**5-(4-Methyl-1-(3,4,5-trihydroxybenzyl)-1*H*-benzo[*d*]imidazol-2-yl)benzene-1,2,3-triol 8s.** Pale yellow solid. M.p. 200–202 °C. ^1^H NMR (600 MHz, DMSO): δ 7.04–6.98 (m, 3H), 6.72 (s, 2H), 5.98 (s, 2H), 5.27 (s, 2H), 2.56 (s, 3H) ppm. ^13^C NMR (151 MHz, DMSO): δ 153.52, 146.73, 146.61, 146.45, 140.69, 135.94, 135.69, 135.45, 128.63, 127.94, 127.54, 121.19, 109.24, 109.00, 108.75, 105.32, 60.23, 21.23 ppm. HRMS(ESI): m/z calc. for C_21_H_18_N_2_O_6_ : 394.1165, found [M + H]^+^ 395.1206.

**5-Chloro-1-(4-chloro-3-nitrobenzyl)-2-(4-chloro-3-nitrophenyl)-1*H*-benzo[*d*]imidazole 10 [30]**. Yellow solid. M.p. 183–185 °C. ^1^H NMR (600 MHz, DMSO): δ 8.83 (d, *J* = 2.1 Hz, 1H), 8.45 (dd, *J* = 8.5, 2.1 Hz, 1H), 8.05–7.96 (m, 1H), 7.85–7.79 (m, 3H), 7.73 (d, *J* = 2.1 Hz, 1H), 7.68 (dd, *J* = 8.5, 6.5 Hz, 1H), 7.30 (dd, *J* = 8.6, 2.0 Hz, 1H), 5.75 (s, 2H) ppm. ^13^C NMR (151 MHz, DMSO): δ 148.72, 147.52, 140.34, 140.31, 133.19, 131.87, 130.41, 129.95, 126.60, 124.02, 119.62, 111.64, 108.25, 105.46 ppm. HRMS(ESI): m/z calc. for C_20_H_12_Cl_3_N_4_O_4_ [M + H]^+^: 476.9924, found: 476.9922.

### 3.2. Biology

#### 3.2.1. Materials

The enzymes and reagents for biochemical assays were obtained from Merck, Sigma Aldrich, Milan, Italy.

#### 3.2.2. Enzyme Activity Assays

##### Glucosidase Activity

The α-glucosidase assay was performed as previously described [47]. The enzyme from Saccharomyces cerevisiae (0.125 U/mL) solution was dissolved in 0.1 M sodium phosphate buffer (pH 6.8). Twenty microliters of test samples at various concentrations (0.005–50 μM) were mixed with the enzyme solution in microplate wells and incubated for 15 min at 37 °C. Subsequently, 20 μL of 5 μM *p*-nitrophenyl α-D-glucopyranoside (pNPG) solution in 0.1 M phosphate buffer was added. After incubation at 37 °C for 15 min, the reaction was terminated by adding 50 μL of 0.2 M sodium carbonate solution. α-Glucosidase activity was determined spectrophotometrically at 405 nm on a 96-well microplate reader Fluostar (BMG LABTECH GmbH, Offenburg, Germany) by measuring the amount of *p*-nitrophenol released. DMSO control was used whenever required and the final concentration of DMSO was maintained below 8% *v*/*v*, which was found not to affect the enzyme activity. The IC_50_ values were determined by the interpolation of dose–response curves (Figure S2, Supplementary Material). Acarbose was used as a reference α-glucosidase inhibitor.

##### Amylase Activity

The α-amylase assay was conducted as described in previous studies [32]. A reaction mix containing 60 μL of 50 μM sodium phosphate buffer at pH 7.0, 20 μL of 1 M NaCl and 40 μL of α-amylase from porcine pancreas (1 mg/mL) was used. The solution was incubated in the absence or presence of compounds at 37 °C for 10 min. After incubation, 80 μL of a 2.5 μM 2-chloro-4-nitrophenyl α-D-maltotrioside (CNPG3) solution was added as the substrate and 2-chloro-nitrophenol released by the enzymatic hydrolysis was monitored at 405 nm, using a microplate reader (Fluostar BMG LABTECH GmbH, Offenburg, Germany). The results of all the assays described above were expressed as a percentage of the blank control.

The percentage of inhibition of enzyme activity, for both enzymes, was calculated as (%) by the following equation: A−B/A × 100, where A represents the difference in the absorbance of the control sample between an incubation time of 0 and 1.0 min, and B represents the test sample’s absorbance difference in the same time range.

##### Cell Viability Assay

The cellular cytotoxicity of compounds was investigated using a 3-(4,5-dimethylthiazol-2-yl)-2,5-diphenyl-tetrazolium bromide (MTT) assay as previously reported, with minor modifications [48]. The CaCo-2 were exposed for 24 h to compounds at concentrations ranging from 0 to 10 μM. Then, MTT reagent (0.5 mg/mL in DMEM) was added to each well. The plate was incubated for 3 h at 37 °C. The MTT solution was removed from the culture plate, and 100 μL of DMSO solvent was added to solubilize the water-insoluble formazan crystals formed in the cells. The absorbance was determined at 570 nm using a microplate reader (VANTAstar_BMG LABTECH GmbH, Ortenberg, Germany).

##### Antioxidant Assays

1. DPPH

Radical scavenging activity of the better derivatives was estimated according to the previously reported method [49] with slight modification using the stable DPPH radical.

A solution of 0.1 mM DPPH• radical was added to various concentrations of compounds. The reaction mixture was incubated in the dark for 15 min and, thereafter, the absorbance was recorded at 517 nm (VANTAstar_BMG LABTECH GmbH, Germany). The decrease in absorbance of DPPH•, after addition of test compounds compared to the control, was used to calculate the antioxidant activity.

2. ABTS

This method is based on the capacity of an antioxidant to scavenge the free radical ABTS^+^ [50].

Samples of each compound (5 μL) were added to 245 μL of ABTS solution. The reduction in the blue-green radical ABTS^+^ by hydrogen-donating antioxidants was evaluated by measuring the absorbance at 734 nm (VANTAstar_BMG LABTECH GmbH, Ortenberg, Germany), after one minute of incubation. All the measurements were carried out at least three times.

For each assay, the decrease in absorbance was used to calculate the antioxidant activity, which was expressed as the ability of the antioxidants to scavenge 50% of radical (EC_50_).

### 3.3. In Silico Studies

#### Materials, Methods and Construction of the Simulated Systems

For the α-glucosidase from *Saccharomyces cerevisiae*, since an experimental structure was not available, a homology model was constructed using the SWISS-MODEL Workspace/GMQE [51]. This model was based on a template (PDB ID: 3A47) from the same species, with a sequence identity of 72.51% compared to the target protein. The quality of the homology model was assessed through local quality estimates and visualized using a Ramachandran plot (Figure S10, Supplementary Material).

Subsequently, the structure was prepared, optimized, and minimized using the PrepWizard tool of the Schrödinger Suite (Schrödinger release 2024-4, Schrödinger, LLC, New York, NY, USA), setting the pH to 7.4. The 3D structures of the compounds under investigation were prepared using the LigPrep tool of the Schrödinger Suite (Schrödinger release 2024-4, Schrödinger, LLC, NY, USA), also setting the pH to 7.4 and generating protonation states using Epik.

Molecular docking simulations were performed using Glide of the Schrödinger Suite (Schrödinger release 2024-4, Schrödinger, LLC, NY, USA) at its highest precision level (Extra Precision, XP). For the generation of conformers, the “Enhanced” sampling was used, and for the selection of initial poses, an “Expanded” sampling was adopted. For docking validation, we used the crystal structures 8YIF and 8YIE, complexed with Acarviosin and Acarbose, respectively. The decision to avoid the exclusive use of acarbose stemmed from its considerable size as a ligand, which would not adequately represent our molecules. In contrast, Acarviosin facilitates simpler comparisons. The ligands demonstrated a remarkable overlap with the crystallographic ligands and achieved a favorable docking score. The protein derived from homology modeling exists in the apo form, which significantly differs in both energetic and conformational aspects from ligand-bound complexes. To enhance its suitability for docking, we performed an overlap with Acarviosin, followed by optimization and minimization processes to relax the structure in the presence of a potential ligand. In this instance, a redocking procedure utilizing Acarviosin was conducted, yielding an outstanding score and significant overlap when compared to the docking executed with the protein in its apo form. Additionally, a control docking with acarbose was carried out, which showed good overlap and an excellent score (Figure S11, Supplementary Material).

Molecular dynamics simulations were performed using Desmond (Schrödinger release 2024-4, Schrödinger, LLC, NY, USA). The systems for the dynamics were prepared using an orthorhombic box with TIP3P water molecules, and the total charge of the system was neutralized by adding Na^+^ and Cl^−^ ions to reach physiological ionic concentration. The OPLS_2005 force field was used. An initial minimization phase was carried out to energetically relax the system. Subsequently, molecular dynamics simulations lasting 500 ns were performed for each ligand.

## 4. Conclusions

In conclusion, we developed a new operationally simple and eco-friendly method for the selective synthesis of 2-aryl-1-arylmethyl-1*H*-benzo[d]imidazole derivatives as α-glucosidase inhibitors. All reactions proceeded rapidly at room temperature using low-cost and environmentally benign catalysts such as SA and ASA. Notably, no organic solvents were employed, as the reactions were carried out in water and completed within minutes. The successful use of a wide range of aromatic and heteroaromatic aldehydes underscores the versatility of the method. In many cases, the target products were obtained via straightforward precipitation or crystallization, eliminating the need for time-consuming and solvent-intensive purification steps.

Selected compounds demonstrated promising α-glucosidase inhibitory activity without exhibiting cytotoxic effects, paving the way for the development of a new class of molecules for potential use in anti-diabetic therapy. α-Glucosidase plays a central role in carbohydrate metabolism by catalyzing the hydrolysis of complex carbohydrates into glucose. Its inhibition is a well-established strategy for managing type 2 diabetes, as it slows glucose absorption and mitigates postprandial glycaemic spikes.

The most potent compounds, **8s**, **8k**, and **8r**, were further evaluated for cytotoxicity and antioxidant activity. Results indicate that in addition to demonstrating a good ability to selectively inhibit α-glucosidase, **8r** and **8s** also display antioxidant properties. In this regard, given the well-established role of oxidative stress and carbohydrate metabolism dysregulation in the pathogenesis of diabetes, compounds capable of addressing both aspects might be of significant clinical interest.

Furthermore, the ADMET predictions for the leading compound **8s** and acarbose suggest that both compounds exhibit a favorable pharmacokinetic profile (Table S5, Supplementary Material) and are unlikely to demonstrate significant drug toxicity, thereby supporting the conclusions derived from the experimental cytotoxicity test results.

The best compounds exhibit potent inhibitory activity against α-glucosidase, with IC_50_ values lower than those of acarbose, the standard antidiabetic drug. The compounds do not inhibit α-amylase, indicating a beneficial selectivity that may help mitigate the gastrointestinal side effects commonly associated with excessive α-amylase inhibition. In silico studies suggest that designing 1,2-disubstituted benzimidazoles capable of forming multiple interactions with catalytic residues of α-glucosidase may yield highly active inhibitors. In particular, MD simulations highlight the strategic importance of incorporating functional groups that can form hydrogen bonds with residues such as ASP68 and ASP214, alongside promoting aromatic interactions with key residues, as a promising approach for enhancing inhibitory potency.

## Figures and Tables

**Table 1:** Results of reactions of **1a** with aldehydes **2b**–**j** in the presence of ASA.

** 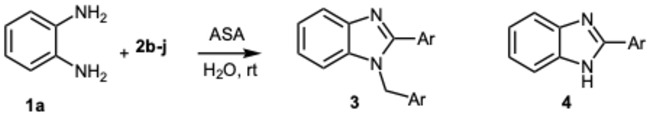 **				
**Entry**	**Aldehyde**	**ASA (Eq)**	**Time (min)**	**Yield ^a^ (%) 3/4**
**1**	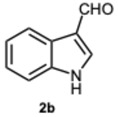	1.26	120	45/0
**2**	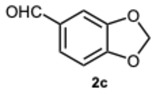	1.26	70	65/0
**3**	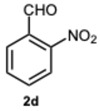	1.26	30	87/0
**4**	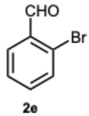	1.26	12	95/0
**5**	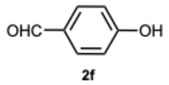	1.26	10	97/0
**6**	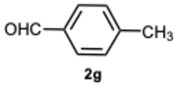	1.26	10	96/0
**7**	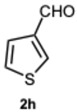	1.26	9	93/0
**8**	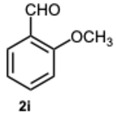	1.26	6	97/0
**9**	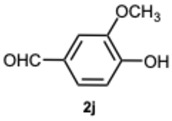	1.26	4	89/0
**10**	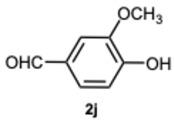	0.1	40	87/0

^a^ Isolated yield.

**Table 2:** Outcomes of the reactions between **1a** and aldehydes **2b-d**, **h-o** in the presence of SA (0.05 eq).

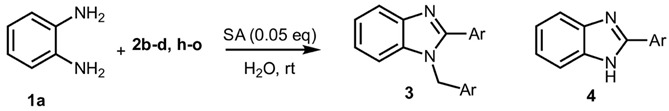			
**Entry**	**Aldehyde**	**Time (min)**	**Yield ^a^ (%) 3/4**
**1**	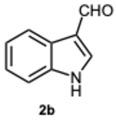	120	55/0
**2**	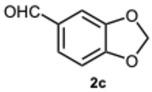	60	92/0
**3**	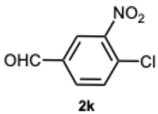	45	81/10
**4**	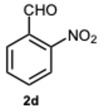	35	87/8
**5**	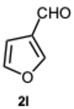	20	96/0
**6**	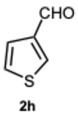	17	95/0
**7**	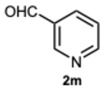	15	85/10
**8**	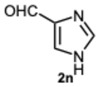	12	91/0
**9**	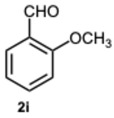	12	96/0
**10**	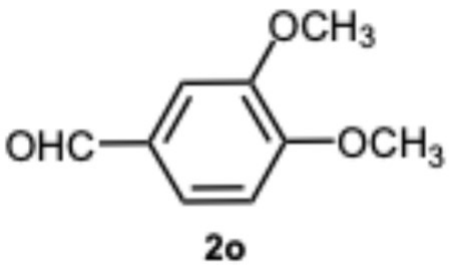	10	97/0

^a^ Isolated yield.

**Table 3:** Reactions of **1c** and aldehydes **2a**, **i, k, p-s** using SA (0.05 eq) as catalyst.

** 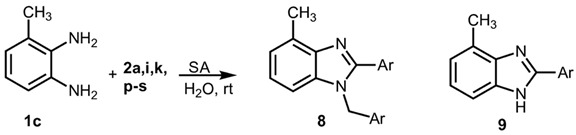 **			
**Entry**	**Aldehyde**	**Time (min)**	**Yield ^a^ (%) 8/9**
**1**	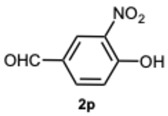	60	85/0
**2**	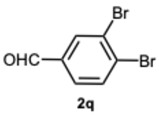	60	90/0
**3**	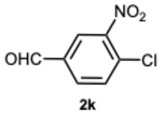	45	75/0
**4**	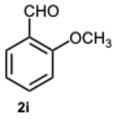	13	95/0
**5**	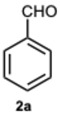	12	93/0
**6**	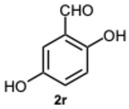	10	84/0
**7**	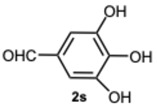	10	78/0

^a^ Isolated yield.

**Table 4:** α-Amylase and α-Glucosidase inhibitory activity of compounds **3k**, **8i**, **k**, **p**-**s**, and **10**.

Compound	α-Amylase % inib. at 50 μM	α-Glucosidase % inib. at 50 μM
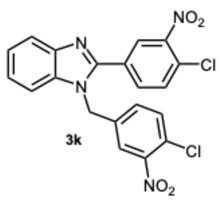	10.2 ± 6.41	9.2 ± 1.6
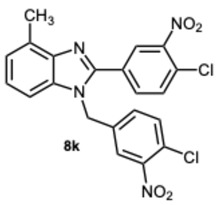	14 ± 0.15	71.2 ± 11.4
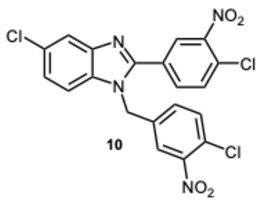	15.8 ± 0.5	17.3 ± 0.4
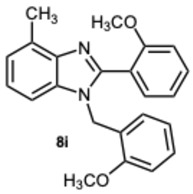	3.2 ± 2.3	35.3 ± 6.8
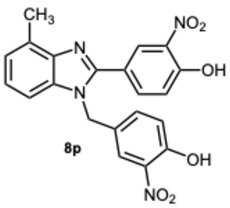	4.3 ± 2.1	3.7 ± 1.4
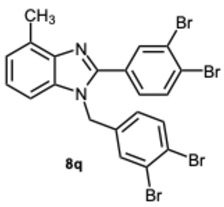	35.7 ± 3	38.2 ± 7.4
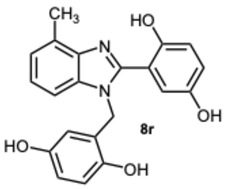	1.3 ± 0.3	94.2 ± 2.8
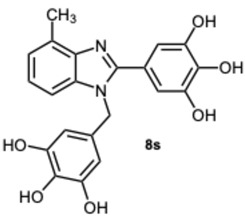	NI	97.5 ± 0.5

NI = None Inhibition. % value represents the mean ± standard deviation for three independent assays.

**Table 5:** α-Glucosidase IC_50_ of compounds **8k**, **8r**, and **8s**. The IC_50_ value was calculated for compounds with % Inhibition ≥ 70.

Compound	IC_50_ (μM)
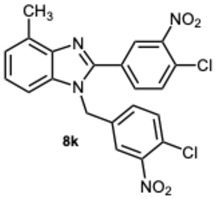	7.1 ± 2.1
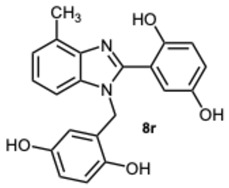	13.8 ± 2.7
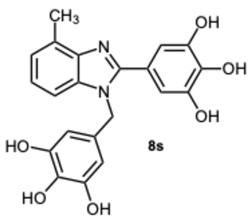	0.39 ± 0.04
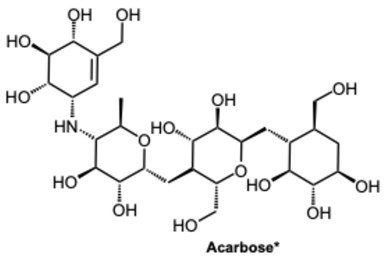	* 139 ± 11.3 [33]

* Standard inhibitor. IC_50_ value represents the mean ± standard deviation for three independent assays.

**Table 6:** Antioxidant activity of compounds **8k**,**8r**, and **8s**.

Compound	DPPH EC_50_ (µM) ^a^	ABTS EC_50_ (µM) ^a^
**8k**	N.A.	N.A.
**8r**	11.4 ± 1.2	13 ± 0.2
**8s**	7.4 ± 0.2	4.6 ± 0.2
**Trolox ^b^**	25.3 ± 0.9	24.4 ± 0.2

N.A. = no antioxidant activity. ^a^ Data represent the mean (±standard deviation, SD) of three independent experiments. ^b^ Positive control.

**Table 7:** Docking-XP Score of selected compounds **3k**, **8i**, **k**, **p**–**s**, and **10**.

Compound	Docking Score (Kcal/mol)
**8s**	−9.926
**8r**	−8.767
**8k**	−7.942
**8p**	−6.665
**8i**	−5.730
3**k**	−5.585
**8q**	−4.953
10	−2.794

## Data Availability

The original contributions presented in the study are included in the article and Supplementary Material, further inquiries can be directed to the corresponding author.

## Supplementary Materials

The following supporting information can be downloaded at: https://www.mdpi.com/article/10.3390/ph18101469/s1. Figure S1. A reasonable mechanism for the formation of 2-aryl-1-arylmethyl-1*H*-benzimidazoles and 2-aryl-1*H*-benzo[*d*]imidazoles. Figure S2. The inhibition plots of **8k**, **8r**, and **8s** compounds. Figure S3. Two-dimensional and 3D representations of the docking pose of compound **8q** within α-glucosidase active site. Figure S4. Two-dimensional and 3D representations of the docking pose of compound **8i** within α-glucosidase active site. Figure S5. Two-dimensional and 3D representations of the docking pose of compound **10** within α-glucosidase active site. Figure S6. Two-dimensional and 3D representations of the docking pose of compound **3k** within α-glucosidase active site. Figure S7. Two-dimensional and 3D representations of the docking pose of compound **8p** within α-glucosidase active site. Figure S8. Three-dimensional representation of regions involved in key fluctuations: blue (residues 200–250), yellow (residues 250–300), gray (residues 325–350), green (300–350), purple (350–400), and red (400–450). Figure S9. Two-dimensional representation of the percentage of interactions that compound **8s** (A), **8k** (B) and **8r** (C) form during the simulation. Red ball (charged positive), Blue ball (charged negative), Cyan ball (neutral), Green ball (hydrophobic), Green line (π-π), Red line (π-cation), Red-Bue line (salt bridge), Grey shadow (solvent exposure), Purple line (hydrogen bond). Figure S10. Two-dimensional representation of the percentage of interactions that compound **8q** (A), **8i** (B), **10** (C), **3k** (D) and **8p** (E) form during the simulation. Red ball (charged positive), Blue ball (charged negative), Cyan ball (neutral), Green ball (hydrophobic), Green line (π-π), Red line (π-cation), Red-Bue line (salt bridge), Grey shadow (solvent exposure), Purple line (hydrogen bond). Figure S11. Ramachandran plot of the homology model of α-glucosidase, comparison with a non-redundant set of PDB structures, and local quality estimate. Figure S12. Left: Overlap of acarviosin with docking score; Right: Overlap of acarbose with docking score. Table S1. Optimization of reaction conditions for the synthesis of 1-benzyl-2-phenyl-1*H*-benzo[d]imidazole **3a** in the presence of ASA. Table S2. Optimization of reaction conditions for the synthesis of 1-benzyl-2-phenyl-1*H*-benzo[*d*]imidazole **3a** in the presence of SA. Table S3. Reactions of **1b** and aldehydes **2a**,**i** using SA as catalyst. Table S4. Reactions of **1b** and **2k** in the presence of SA as catalyst. Table S5. Predicted ADMET of **8s** against Acarbose. ^1^H, ^13^C NMR spectra of all compounds and 2D-Noesy NMR of compound **8k**. HRMS Spectra of most active compounds **8s** and **8k**. References [52,53] are cited in the Supplementary Materials.

## Abbreviations

The following abbreviations are used in this manuscript:

a-GIs	α-Glucosidase Inhibitors
ASA	Acetylsalicylic Acid
SA	Salicylic Acid
MD	Molecular Dynamics
IDE	Insulin-Degrading Enzyme
ABTS	2, 2′-Azino-bis(3-Ethylbenzothiazoline-6-Sulfonic Acid
DPPH	2,2-Diphenyl-1-Picrylhydrazyle
ASP	Aspartic Acid
ARG	Arginine
PHE	Phenylalanine
LYS	Lysine
GLU	Glutamic Acid
ASN	Asparagine
HIE	Histidine with Hydrogen on the Epsilon Nitrogen
HIS	Hystidine
TLC	Thin-Layer Chromatography
AcOEt	Ethyl Acetate
DCM	Dichloromethane
DMSO	N,N- Dimethylsulfoxide
CNPG3	2-Chloro-4-Nitrophenyl a-D-Maltotrioside
MTT	3-(4,5-Dimethylthiazol-2-yl)-2,5-Diphenyl-Tetrazolium Bromide
