# Supplementary material for: Sustainable Synthesis of 1,2-Disubstituted Benzimidazoles as Promising α-Glucosidase Inhibitors: In Vitro and In Silico Evaluation
Source: Pharmaceuticals (Basel). 2025 Sep 30;18(10):1469. doi: 10.3390/ph18101469 (PMC12567066; doi:10.3390/ph18101469)
Supplement: Supplementary file 1 [file pharmaceuticals-18-01469-s001.zip › pharmaceuticals-3839031-supplementary.pdf]

Sustainable Synthesis of 1,2-Disubstituted Benzimidazoles as Promising a-Glucosidase Inhibitors: In Vitro and In Silico Evaluation

Graziella Tocco, [1,*] Antonio Laus, [1,2] Cristina Manis,[1] Pierluigi Caboni,[1] Antonella Fais,[1] Benedetta Era.[1]

[1]Department of Life and Environmental Sciences, University of Cagliari, Cittadella Universitaria di Monserrato, Monserrato, Cagliari, Italy;

[b]Center for Advanced Studies Research and Development in Sardinia (CRS4), 09050 Pula, Cagliari, Italy (Current Address);

Correspondence: toccog@unica.it

TABLE OF CONTENTS

Experimental section

1. ***Chemistry***

-Mechanism of formation of 2-aryl-1-arylmethyl-1H-benzimidazoles and 2-aryl-1H-benzimidazoles(Figure 1S)..p. 3

-Tables 1S,2S, 3S and 4S……pp 4-7

-^{1}H and ^{13}C NMR spectra……. pp 8-62

-HRMS of most active compounds **8s** and **8k**……………………………………………………………………………………..p 63

2. ***Biology***

- Predicted ADMET of **8s** against Acarbose)……………………………………………………………………………………..pp 64-65
- Figure 2S. The inhibition plots of compounds **8k**, **8r**, and **8s** ……………………………………………………………..p66

3. ***In silico studies***

- Two-dimensional and 3D representations of the docking pose of compound **8q** within α-glucosidase active site. (Figure 3S)……..p 67

- Two-dimensional and 3D representations of the docking pose of compound **8i** within α-glucosidase active site. (Figure 4S) …… p 68
- Two-dimensional and 3D representations of the docking pose of compound **10** within α-glucosidase active site. (Figure 5S) ……...p 69
- Two-dimensional and 3D representations of the docking pose of compound **3k** within α-glucosidase active site. (Figure 6S) ………p 70
- Two-dimensional and 3D representations of the docking pose of compound **8p** within α-glucosidase active site. (Figure 7S) ……p 71
-3D representation of regions involved in key fluctuations: blue (residues 200-250), yellow (residues 250-300), grey (residues 325-350), green (300-350), purple (350-400), and red (400-450). (Figure 8S) ……………………………...p 72
- Two-dimensional representation of the percentage of interactions that compound **8s** (A), **8k** (B) and **8r** (C) form during the simulation. Red ball (charged positive), Blue ball (charged negative), Cyan ball (neutral), Green ball (hydrophobic), Green line (π-π), Red line (π -cation), Red-Bue line (salt bridge), Grey shadow (solvent exposure), Purple line (hydrogen bond). (Figure9S)………………… ………………………… ………………………………………………………p 73
- Two-dimensional representation of the percentage of interactions that compound **8q** (A), **8i** (B), **10** (C), **3k** (D) and 8p (E) form during the simulation. Red ball (charged positive), Blue ball (charged negative), Cyan ball (neutral), Green ball (hydrophobic), Green line (π - π), Red line (π cation), Red-Bue line (salt bridge), Grey shadow (solvent exposure), Purple line (hydrogen bond). (Figure 10S)…………………… ……………………………………… …………………p 74
-Ramachandran plot of the homology model of α-glucosidase (Figure 11S) ……………..……………………………………..p.75
-Control docking with acarbose(Figure 12S) ……………………………………………………………………………………………. p.76

1. CHEMISTRY

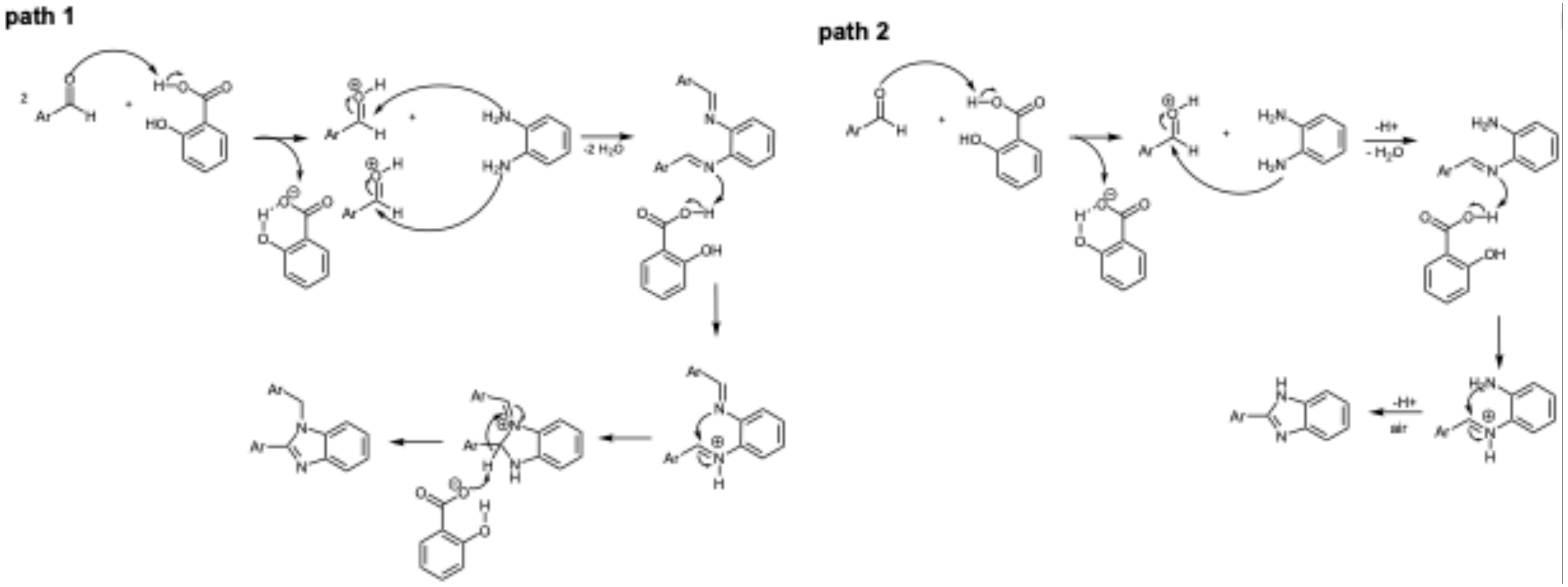

Figure 1S. A reasonable mechanism for the formation of 2-aryl-1-arylmethyl-1H-benzimidazoles (pathway 1) and 2-aryl-1*H*-benzo[*d*]imidazoles (pathway 2)

Table 1S. Optimization of reaction conditions for the synthesis of 1-benzyl-2-phenyl-1*H*-benzo[*d*]imidazole **3a** in the presence of ASA.

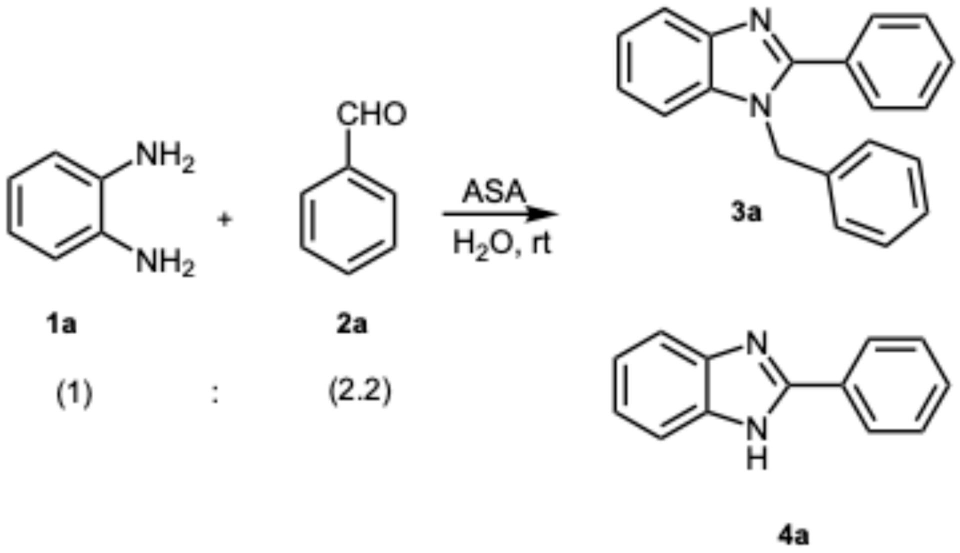

Entry	Catalyst (eq)	Time (min)	Yield(%) [a] 3a/4a
1	0	180	0/10
2	1.26	3	96/ 0
3	1.00	9	95/ 0
4	0.1	15	92/ 0

[a] Isolated yield

Table 2S. Optimization of reaction conditions for the synthesis of 1-benzyl-2-phenyl-1*H*-benzo[*d*]imidazole **3a** in the presence of SA.

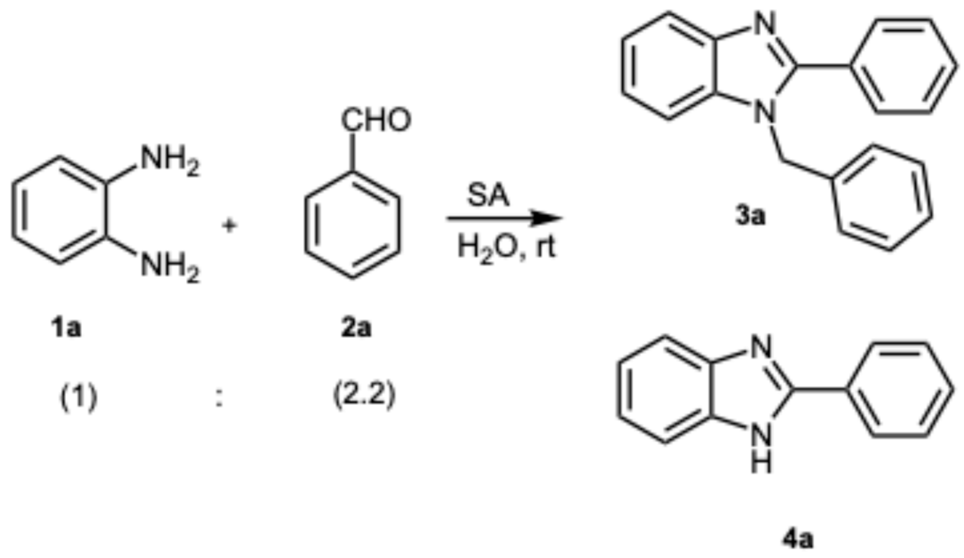

Entry	Catalyst (eq)	Time (min)	Yield (%)[a] 3a/4a
1	1.26	1	96/ 0
2	0.1	1	96/ 0
3	0.05	10	95/ 0
4	0.02	16	92/ 0

[a] Isolated yield

Table 3S. Reactions of **1b** and aldehydes **2a**, **i** using SA as catalyst

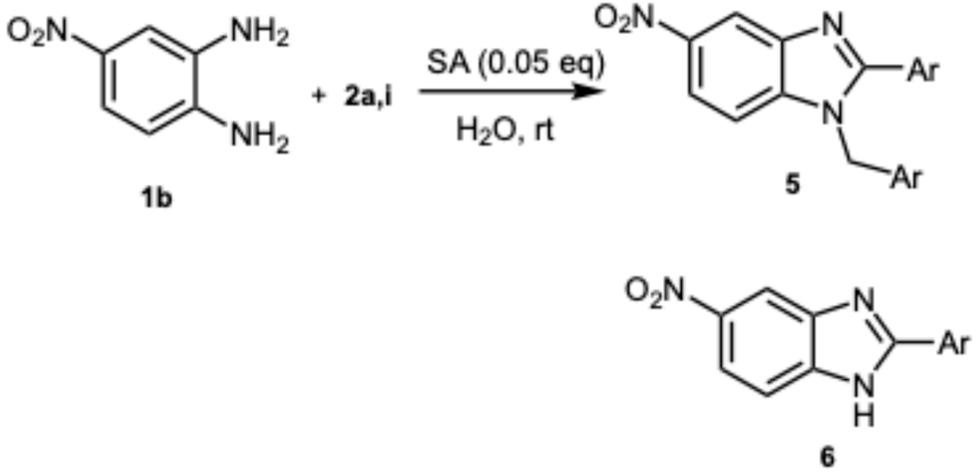

**Entry**	**Aldehyde**	**Time (min)**	**Yield(%)[a] 5/6**
1	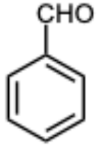 **2a**	30	35/60
2	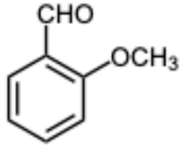 **2i**	13	Traces[b]/89

[a] Isolated yield. [b] GC yield

Table 4S. Reactions of **1b** and **2k** in the presence of SA as catalyst

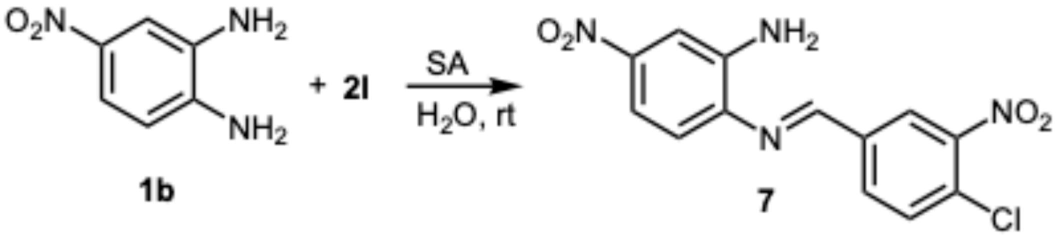

Entry	Aldehyde	SA (eq.)	Time (min)	Yield (%) 7
1	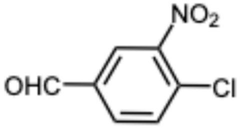 **2k**	0.05	30	95
2	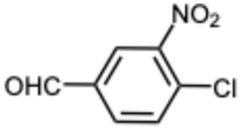 **2k**	0.2	20	92
3	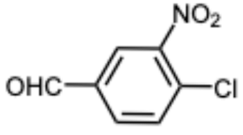 **2k**	0.7	15	92
4	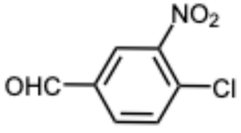 **2k**	1.80	15	97

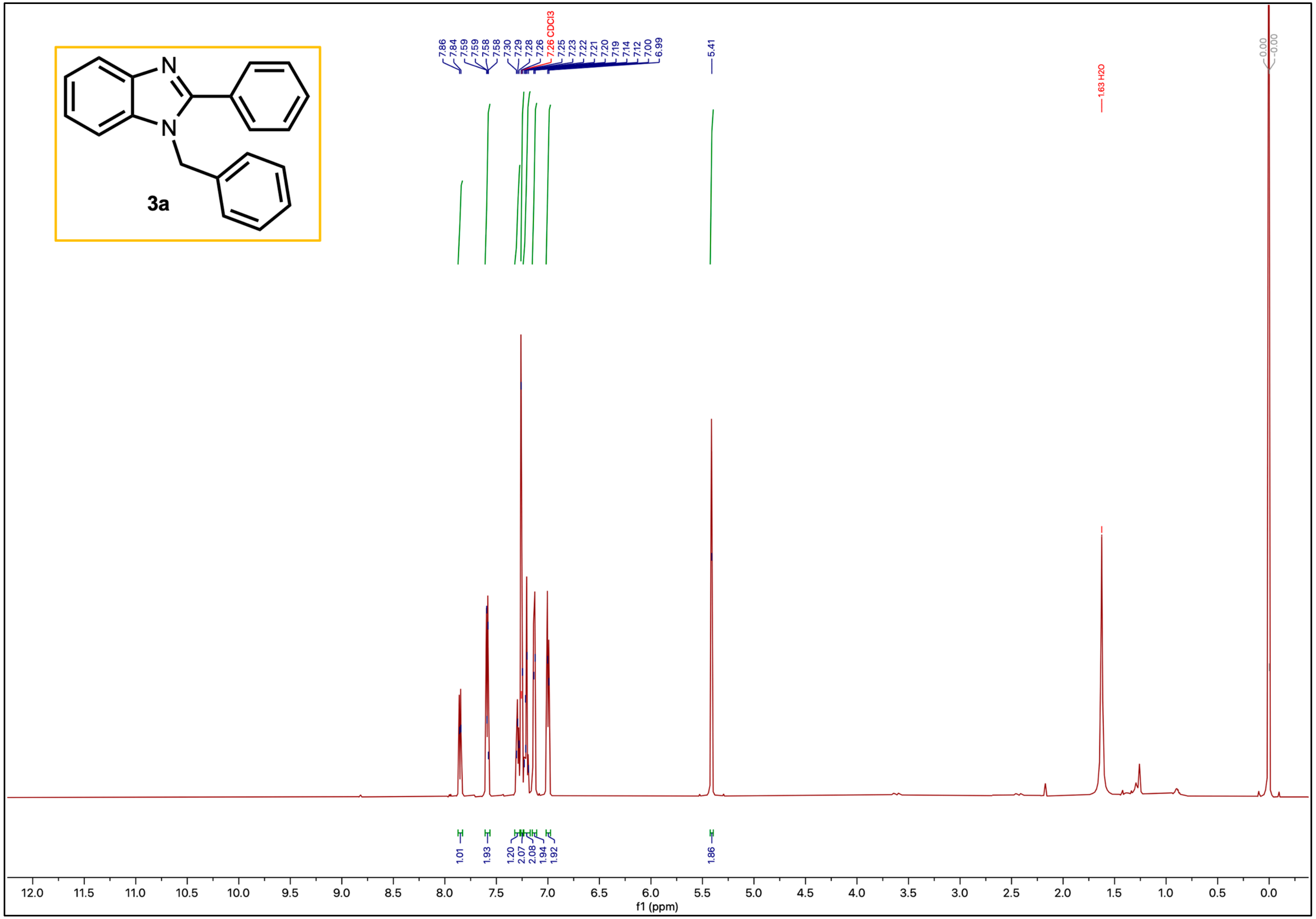
N
N
3a
7.86
7.84
7.59
7.59
7.58
7.58
7.30
7.29
7.28
7.26
7.26 CDCl3
7.25
7.23
7.22
7.21
7.20
7.19
7.14
7.12
7.00
6.99
5.41
1.63 H2O
0.00
0.00
1.01
1.93
1.20
2.07
2.08
1.94
1.92
1.86
12.0 11.5 11.0 10.5 10.0 9.5 9.0 8.5 8.0 7.5 7.0 6.5 6.0 5.5 5.0 4.5 4.0 3.5 3.0 2.5 2.0 1.5 1.0 0.5 0.0
f1 (ppm)

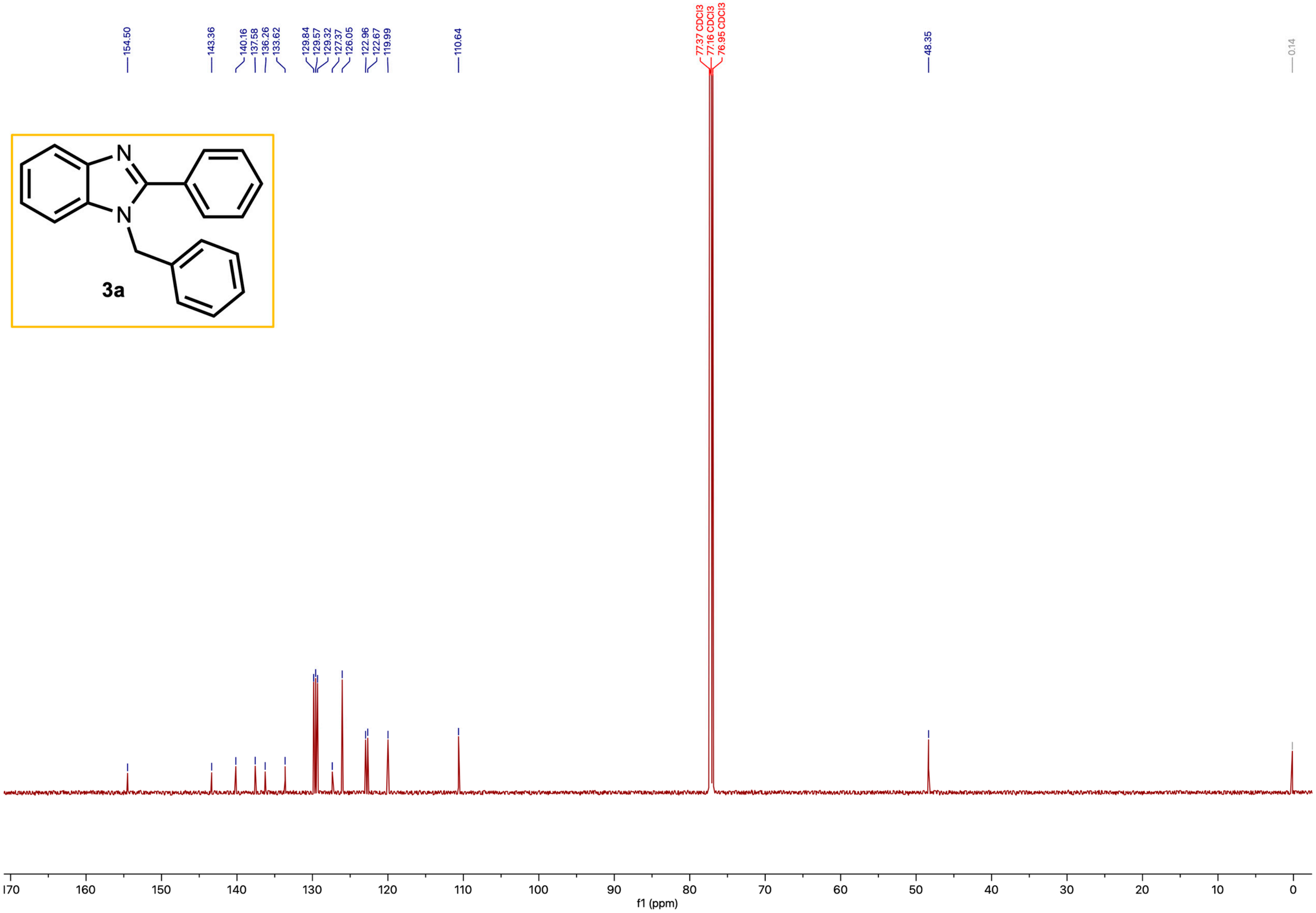
154.50
143.36
140.16
137.58
136.26
133.62
129.84
129.57
129.32
127.37
126.05
122.96
122.67
119.99
110.64
77.37 CDCl3
77.16 CDCl3
76.95 CDCl3
48.35
0.14
N
N
3a
170
160
150
140
130
120
110
100
90
80
70
60
50
40
30
20
10
0
f1 (ppm)

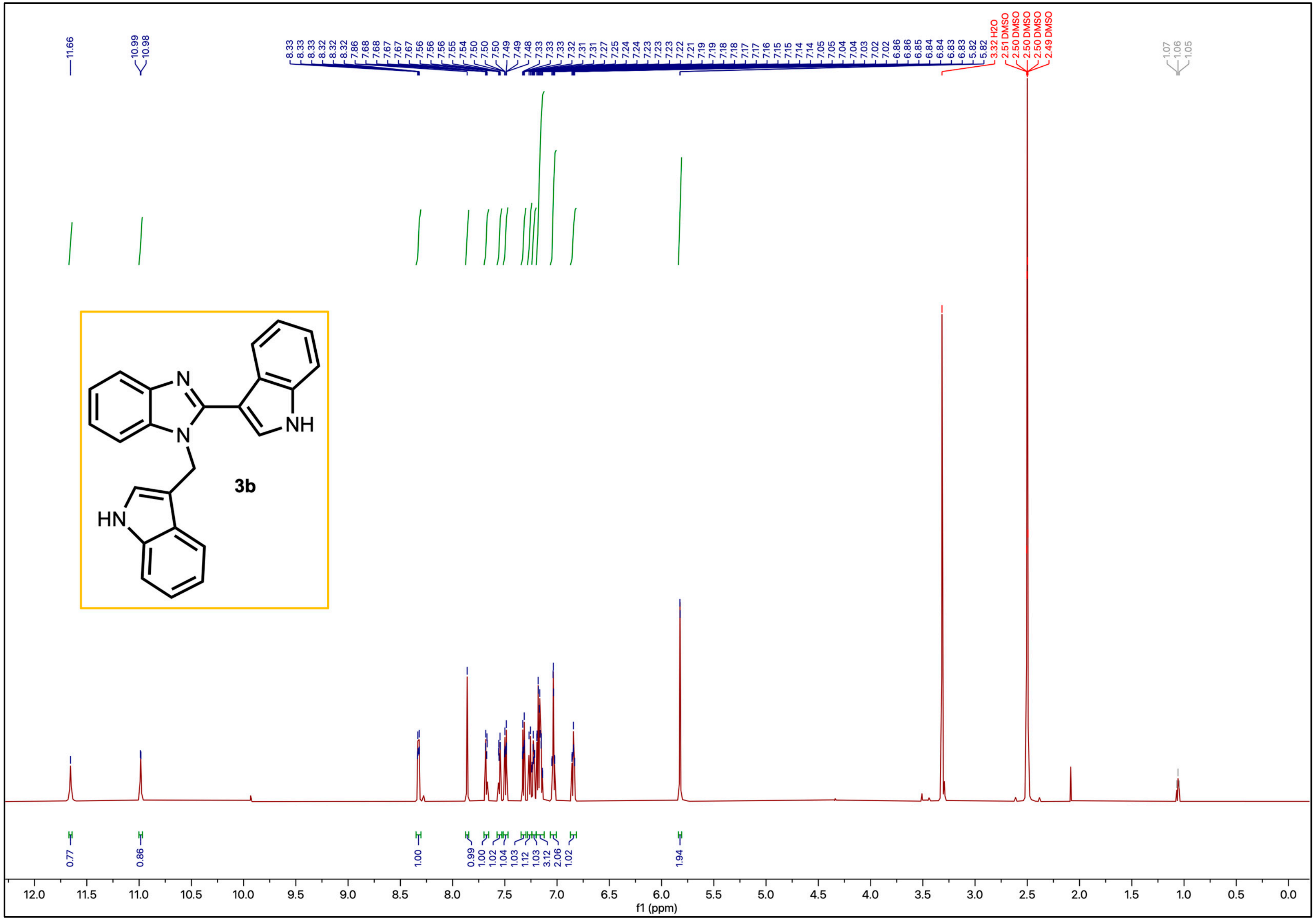
3b
f1 (ppm)

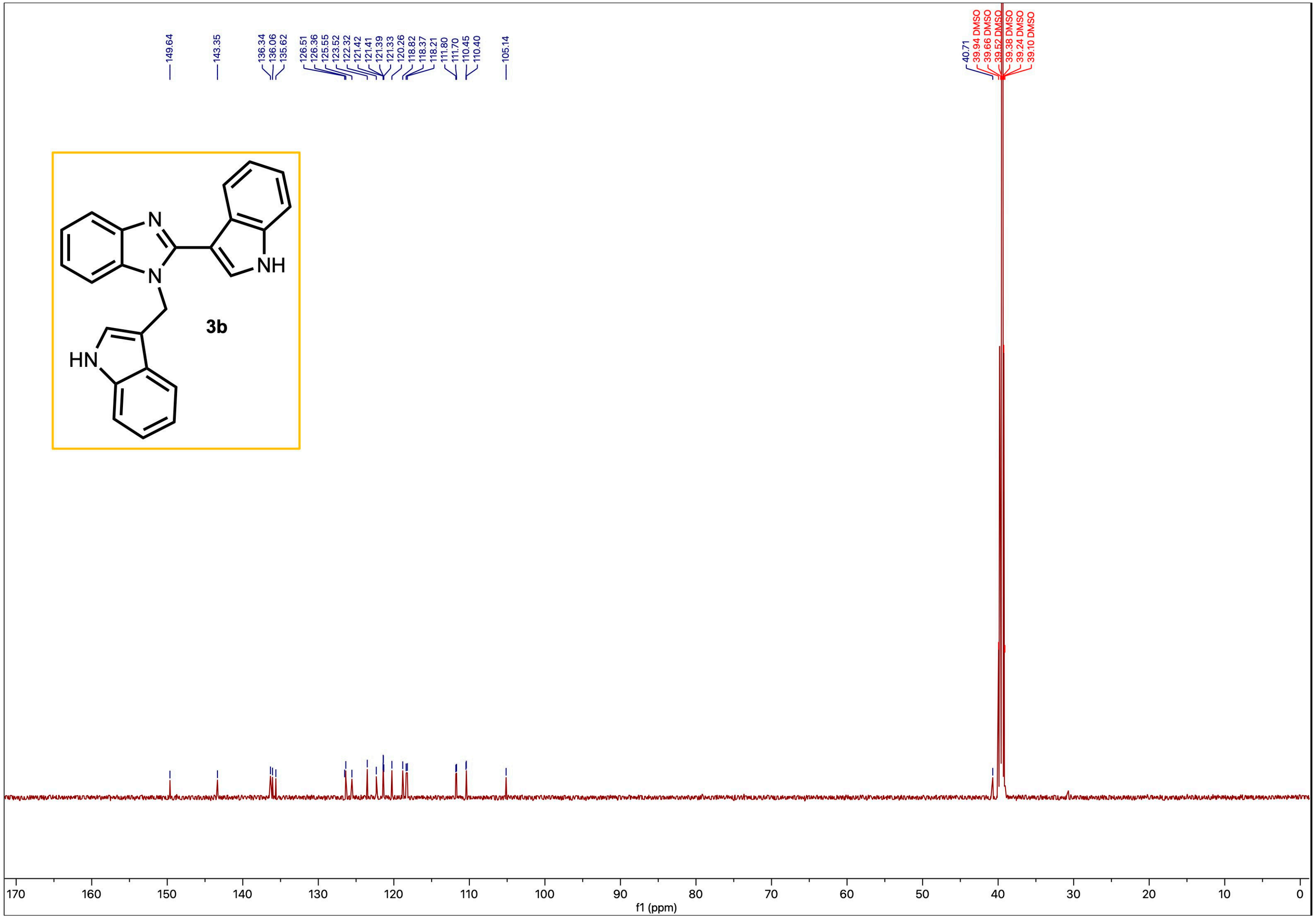
149.64
143.35
136.34
136.06
135.62
126.51
126.36
125.55
123.52
122.32
121.42
121.41
121.39
121.33
120.26
118.82
118.37
118.21
111.80
111.70
110.45
110.40
105.14
40.71
39.94 DMSO
39.66 DMSO
39.52 DMSO
39.38 DMSO
39.24 DMSO
39.10 DMSO
N
NH
N
HN
3b
f1 (ppm)

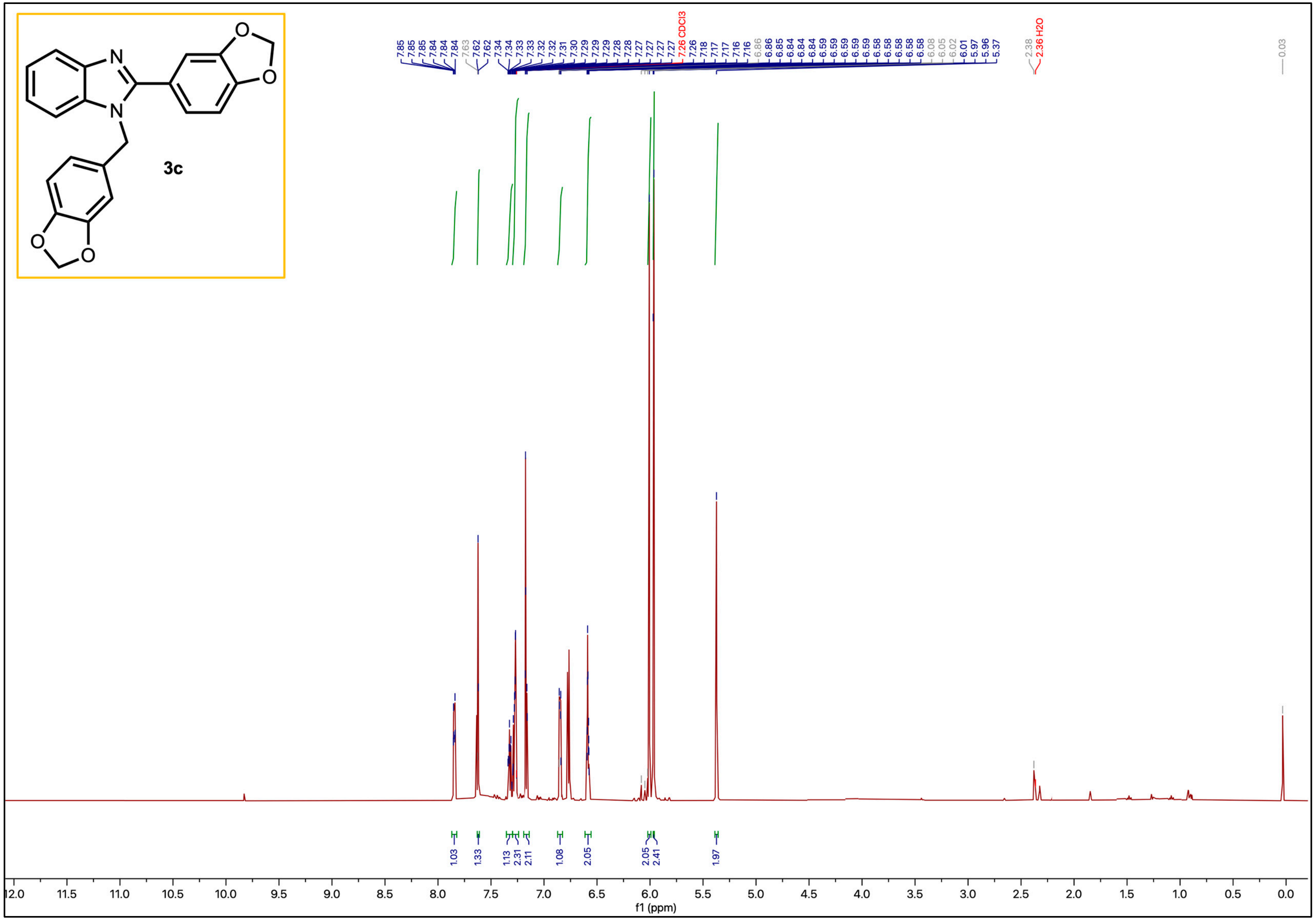
3c
f1 (ppm)

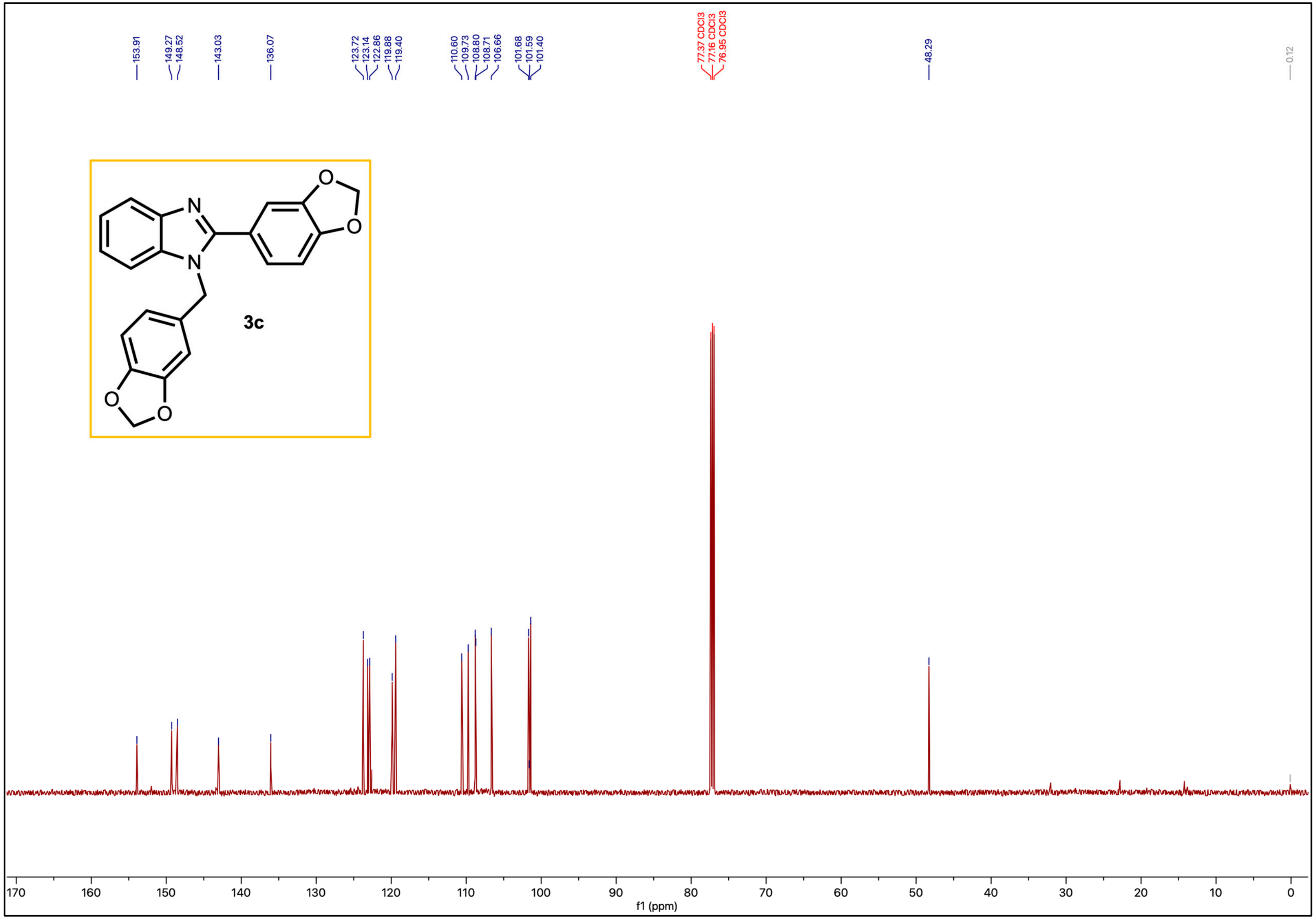
153.91
149.27
148.52
143.03
136.07
123.72
123.14
122.86
119.88
119.40
110.60
109.73
108.80
108.71
106.66
101.68
101.59
101.40
77.37 CDCl3
77.16 CDCl3
76.95 CDCl3
48.29
0.12
N
N
O
O
O
O
3c
170 160 150 140 130 120 110 100 90 80 70 60 50 40 30 20 10 0
f1 (ppm)

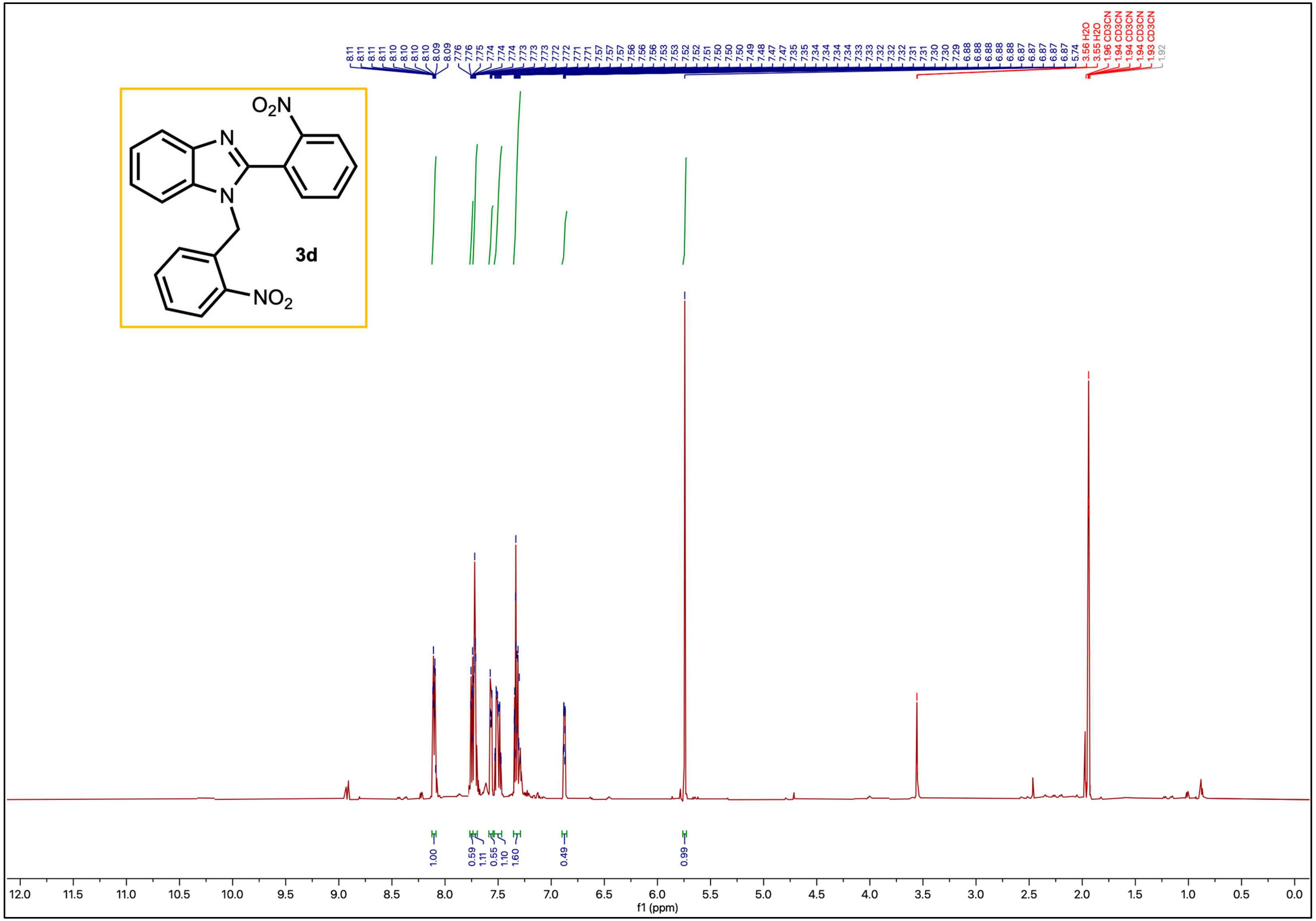

3d
f1 (ppm)

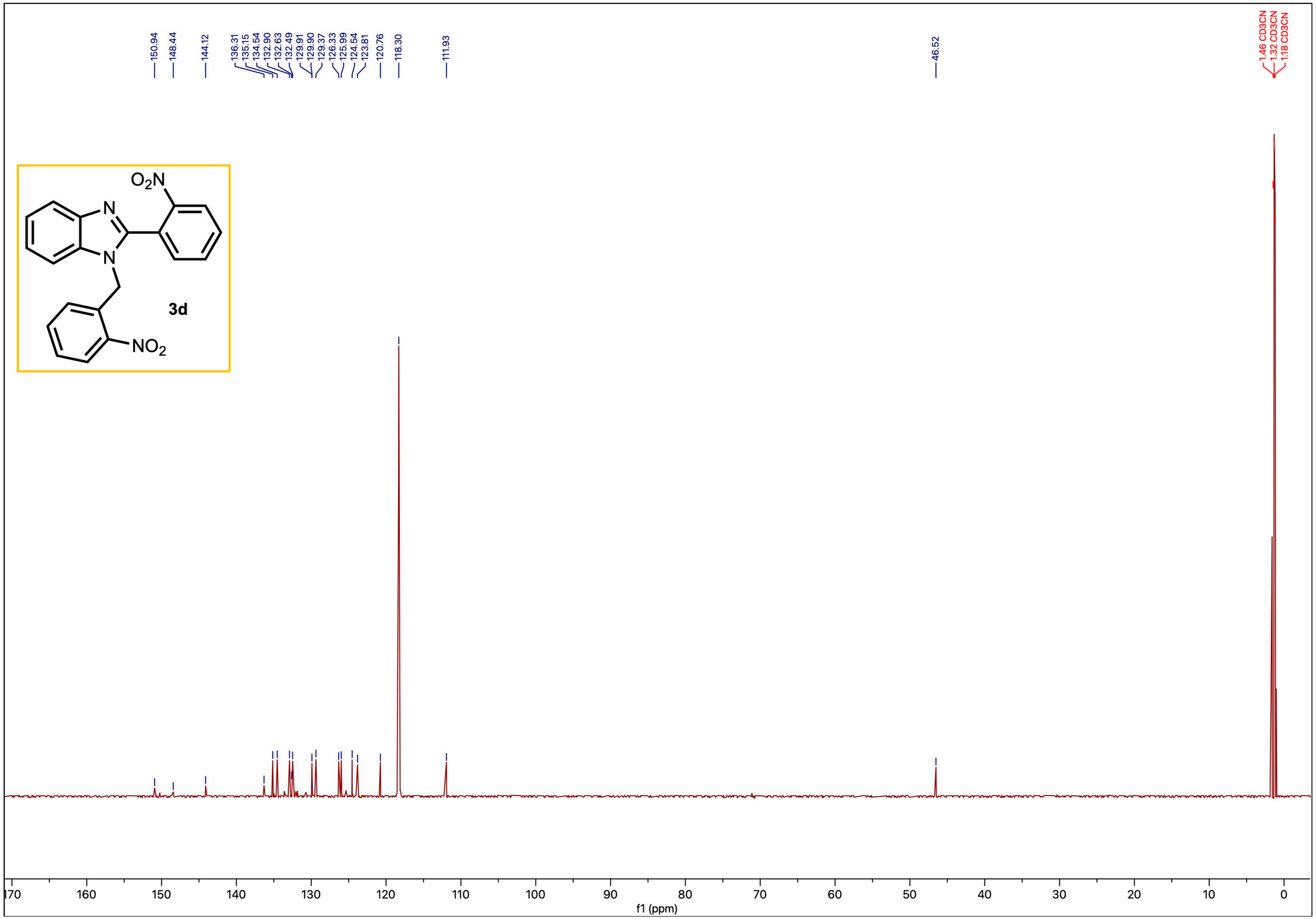

150.94
148.44
144.12
136.31
135.15
134.54
132.90
132.63
132.49
129.91
129.90
129.37
126.33
125.99
124.54
123.81
120.76
118.30
111.93
46.52
1.46 CD3CN
1.32 CD3CN
1.18 CD3CN
O2N
N
N
3d
NO2
f1 (ppm)

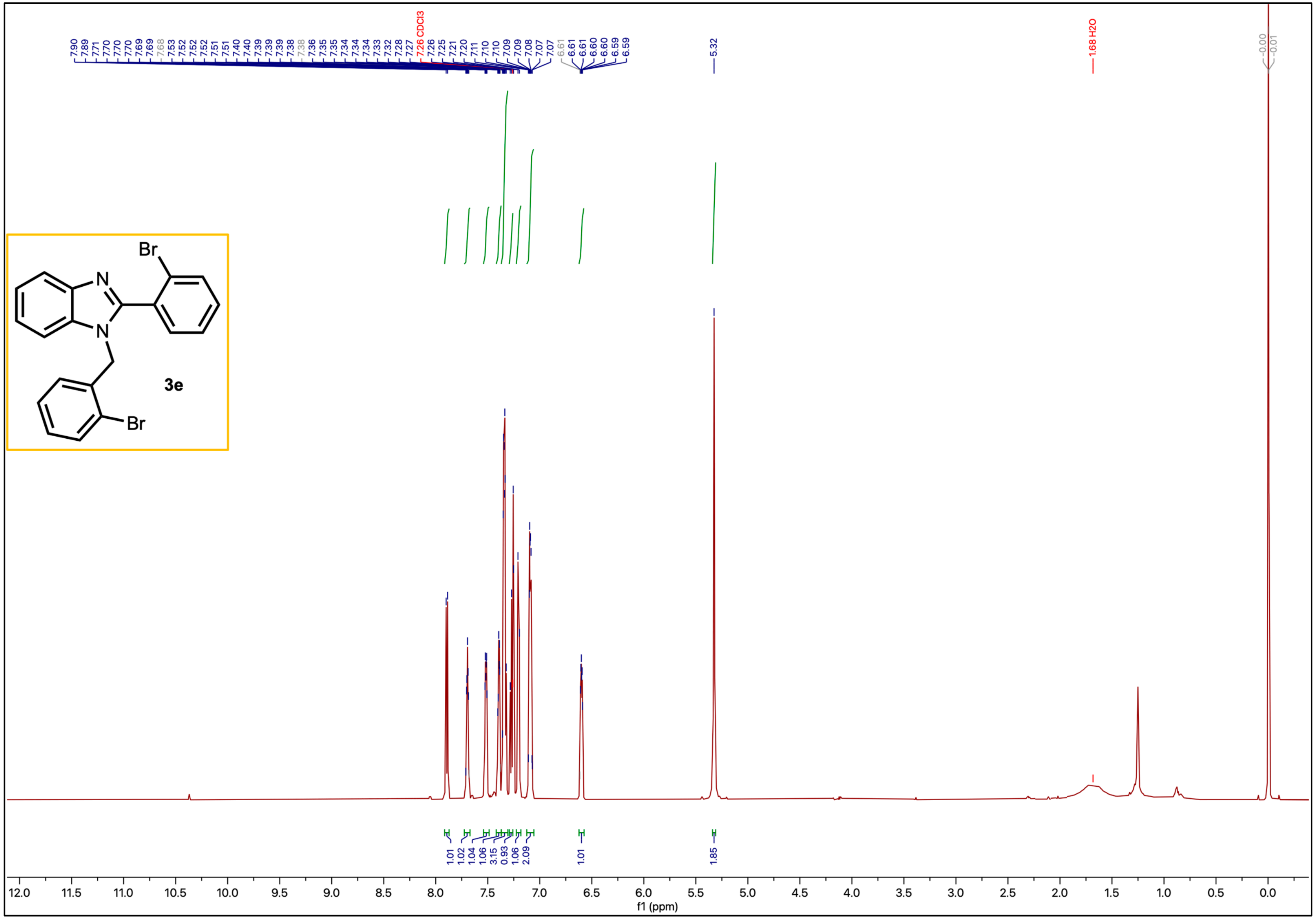

7.90 7.89 7.71 7.70 7.70 7.70 7.69 7.69 7.68 7.53 7.52 7.52 7.52 7.51 7.51 7.40 7.40 7.39 7.39 7.39 7.38 7.38 7.36 7.35 7.35 7.34 7.34 7.34 7.33 7.32 7.28 7.27 7.26 CDCl3 7.26 7.25 7.21 7.20 7.11 7.10 7.10 7.09 7.09 7.08 7.07 7.07 6.61 6.61 6.60 6.60 6.59 6.59
5.32
1.68 H2O
0.00 -0.01
Br
N
N
Br
3e
1.01 1.02 1.04 1.06 3.15 0.93 1.06 2.09 1.01 1.85
f1 (ppm)

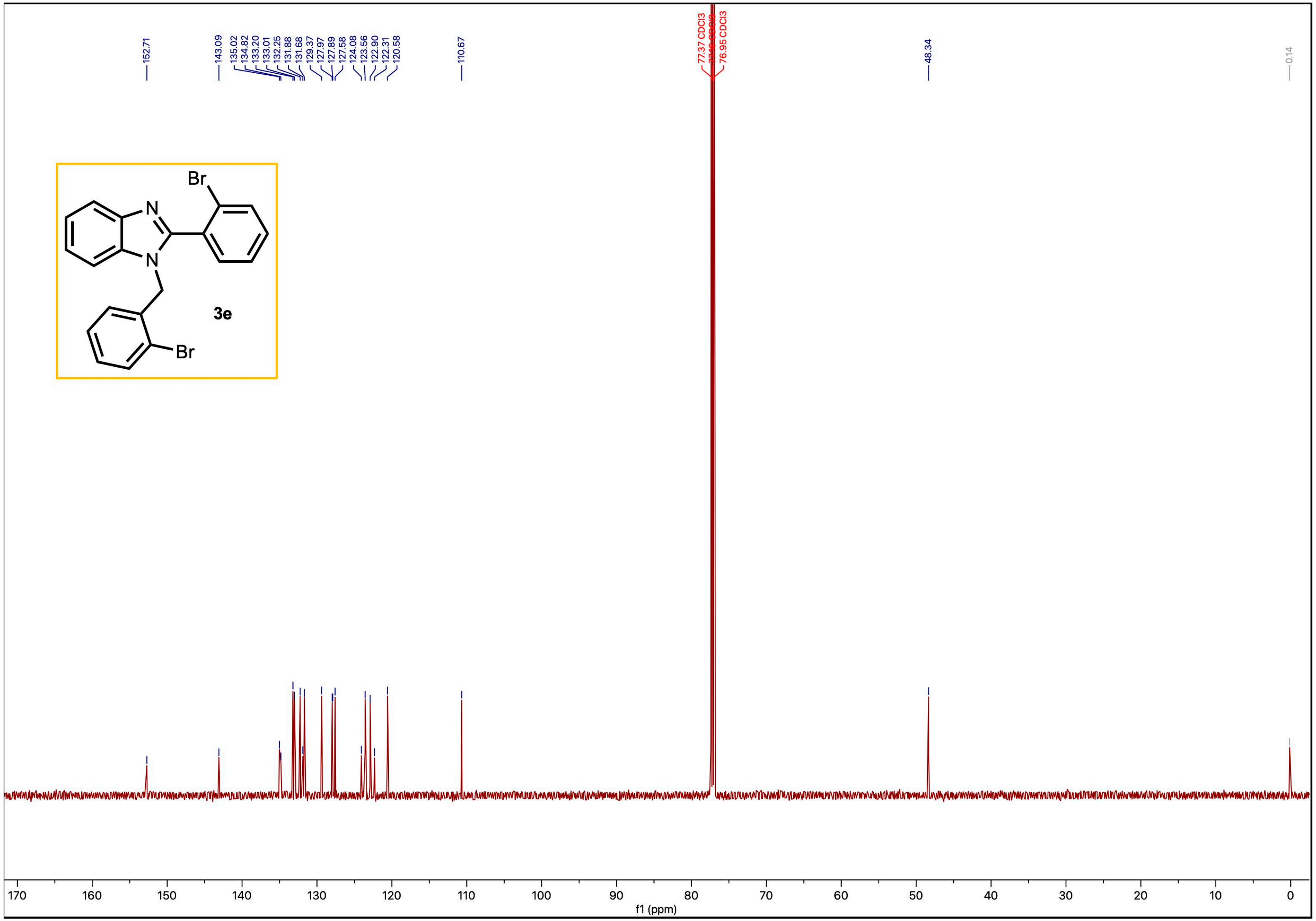
152.71
143.09
135.02
134.82
133.20
133.01
132.25
131.88
131.68
129.37
127.97
127.89
127.58
124.08
123.56
122.90
122.31
120.58
110.67
77.37 CDCl3
76.95 CDCl3
48.34
0.14
Br
N
N
Br
3e
170 160 150 140 130 120 110 100 90 80 70 60 50 40 30 20 10 0
f1 (ppm)

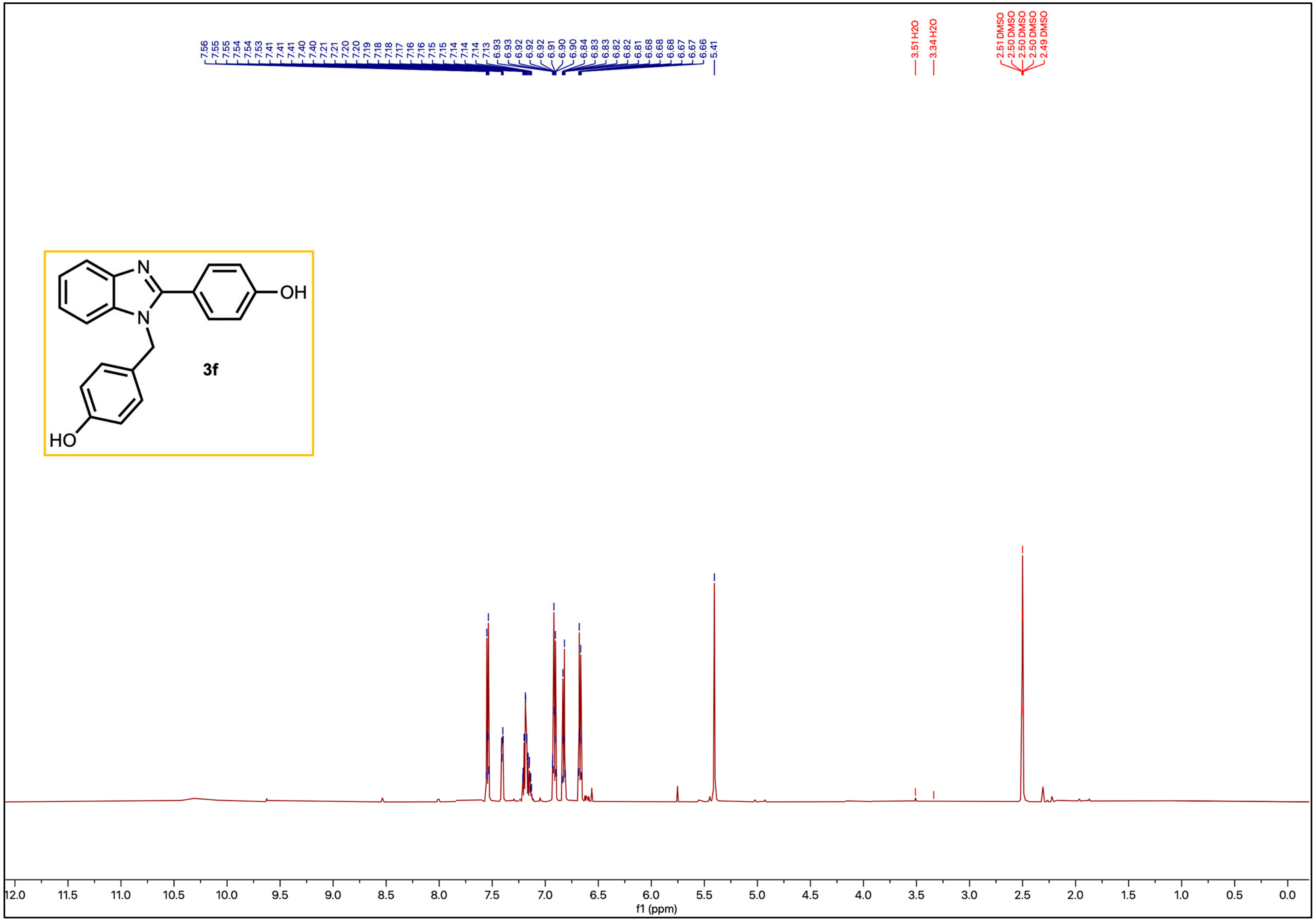
3f
f1 (ppm)

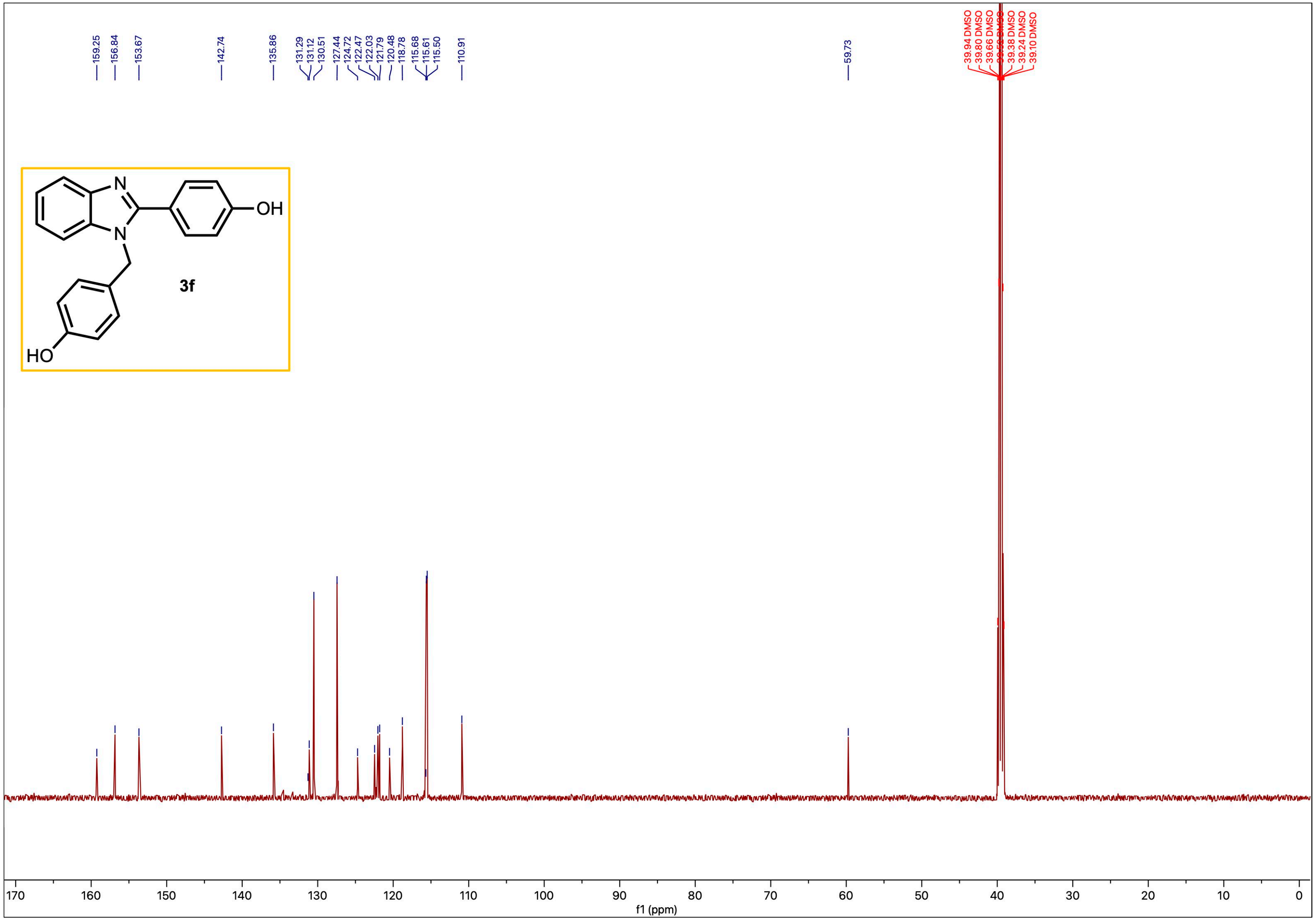

159.25
156.84
153.67
142.74
135.86
131.29
131.12
130.51
127.44
124.72
122.47
122.03
121.79
120.48
118.78
115.68
115.61
115.50
110.91
59.73
39.94 DMSO
39.80 DMSO
39.66 DMSO
39.38 DMSO
39.24 DMSO
39.10 DMSO
N
N
OH
HO
3f
170
160
150
140
130
120
110
100
90
80
70
60
50
40
30
20
10
0
f1 (ppm)

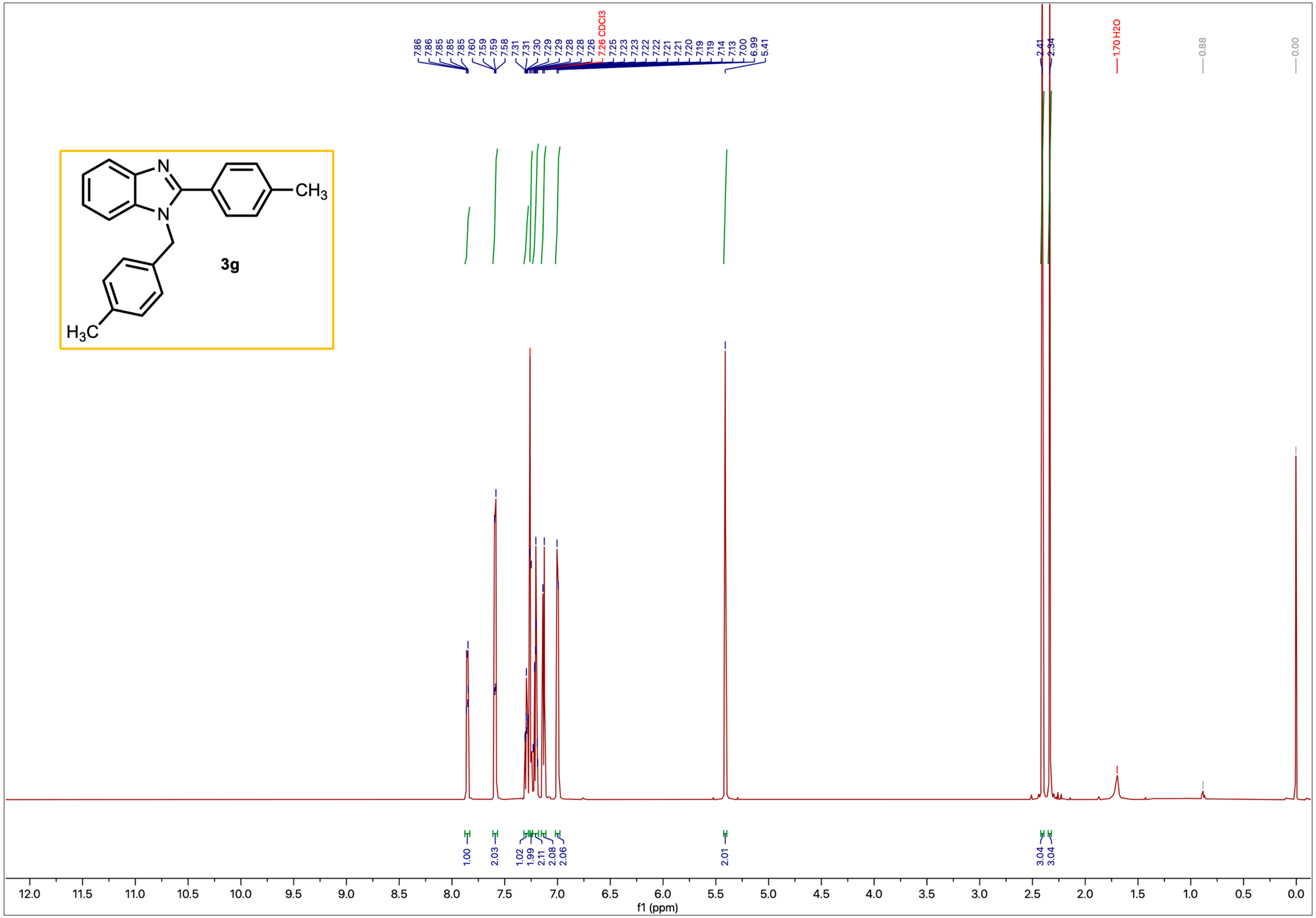

3g
CH3
H3C
7.86 7.86 7.85 7.85 7.85 7.60 7.59 7.59 7.58 7.31 7.31 7.30 7.29 7.29 7.28 7.28 7.26 7.26 CDCl3 7.25 7.23 7.23 7.22 7.22 7.21 7.21 7.20 7.19 7.19 7.14 7.13 7.00 6.99 5.41
2.41 2.34
1.70 H2O
0.88
0.00
1.00 2.03 1.02 1.99 2.11 2.08 2.06 2.01 3.04 3.04
f1 (ppm)

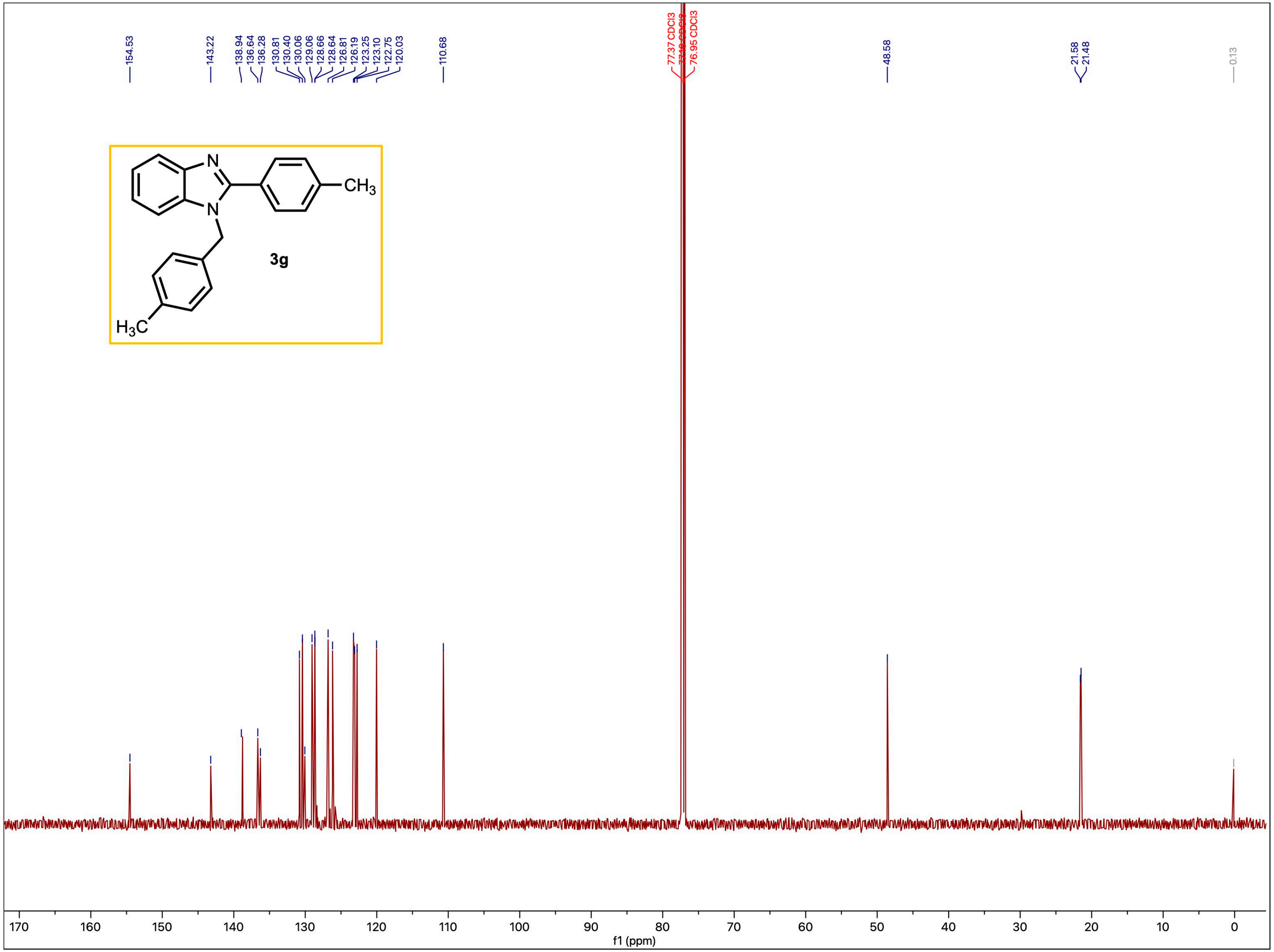

154.53
143.22
138.94
136.64
136.28
130.81
130.40
130.06
129.06
128.66
128.64
126.81
126.19
123.25
123.10
122.75
120.03
110.68
77.37 CDCl3
76.95 CDCl3
48.58
21.58
21.48
0.13
N
N
CH3
H3C
3g
170
160
150
140
130
120
110
100
90
80
70
60
50
40
30
20
10
0
f1 (ppm)

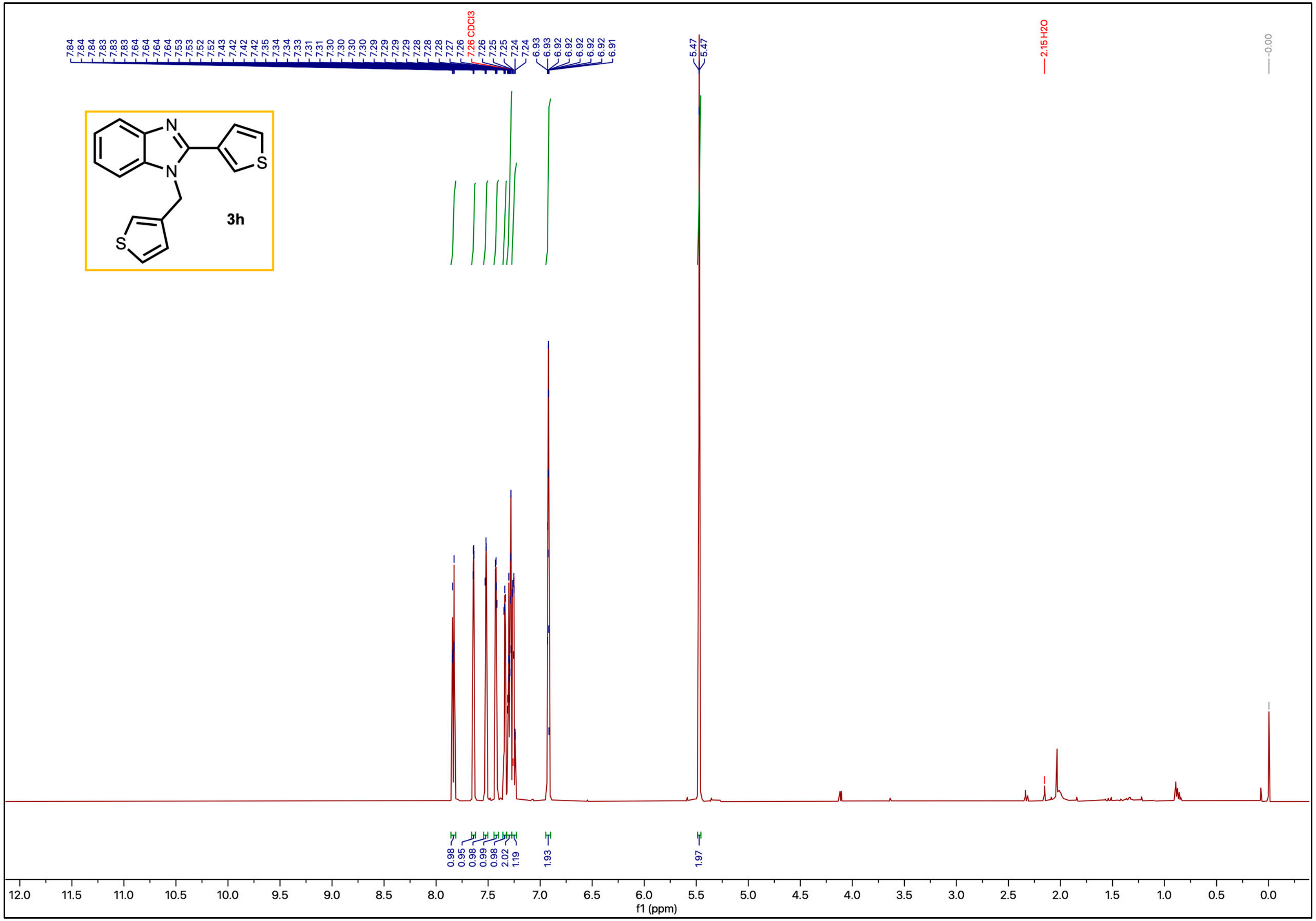

3h
f1 (ppm)
7.26 CDCl3
2.15 H2O

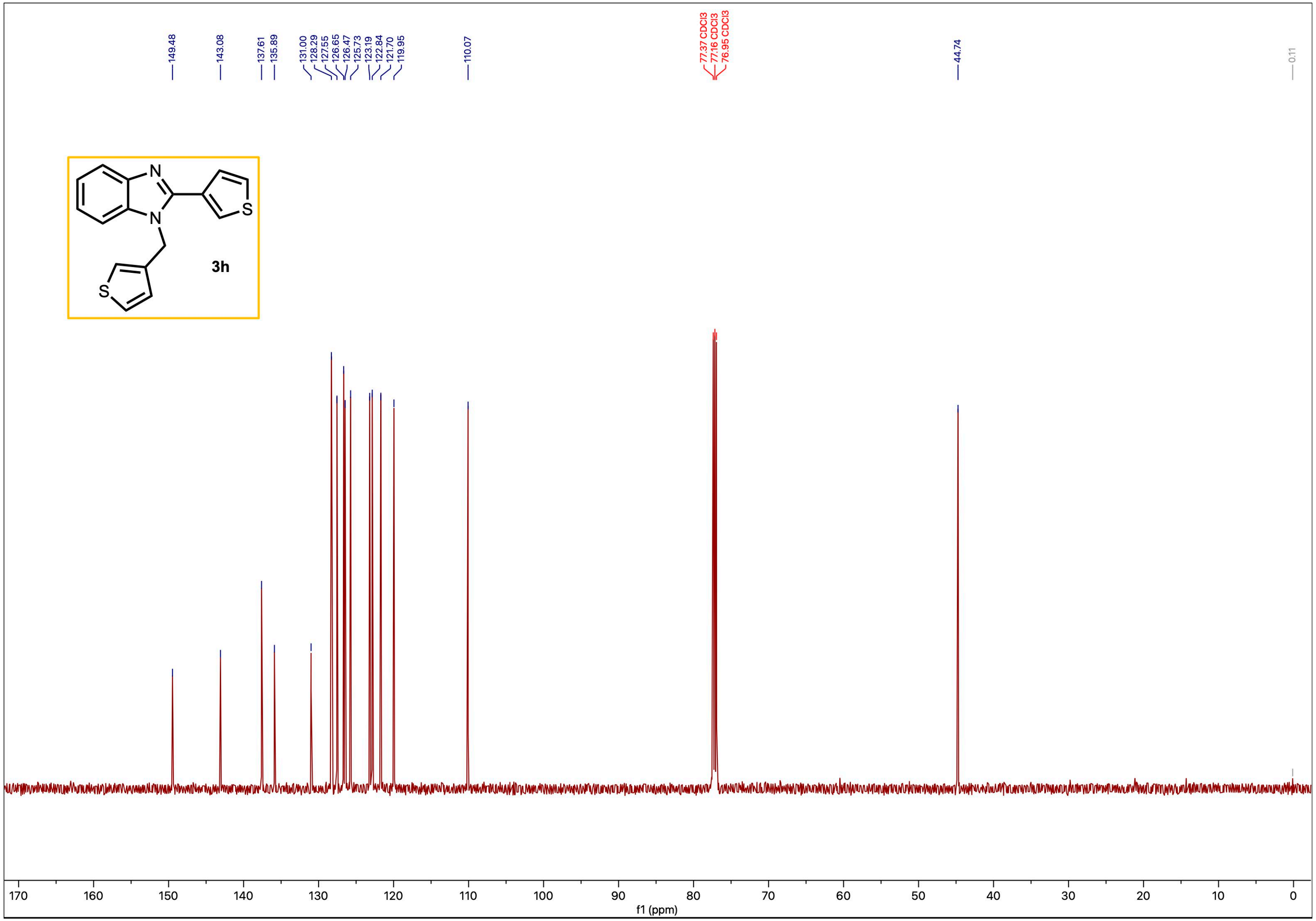
149.48
143.08
137.61
135.89
131.00
128.29
127.55
126.65
126.47
125.73
123.19
122.84
121.70
119.95
110.07
77.37 CDCl3
77.16 CDCl3
76.95 CDCl3
44.74
0.11
N
N
S
S
3h
f1 (ppm)

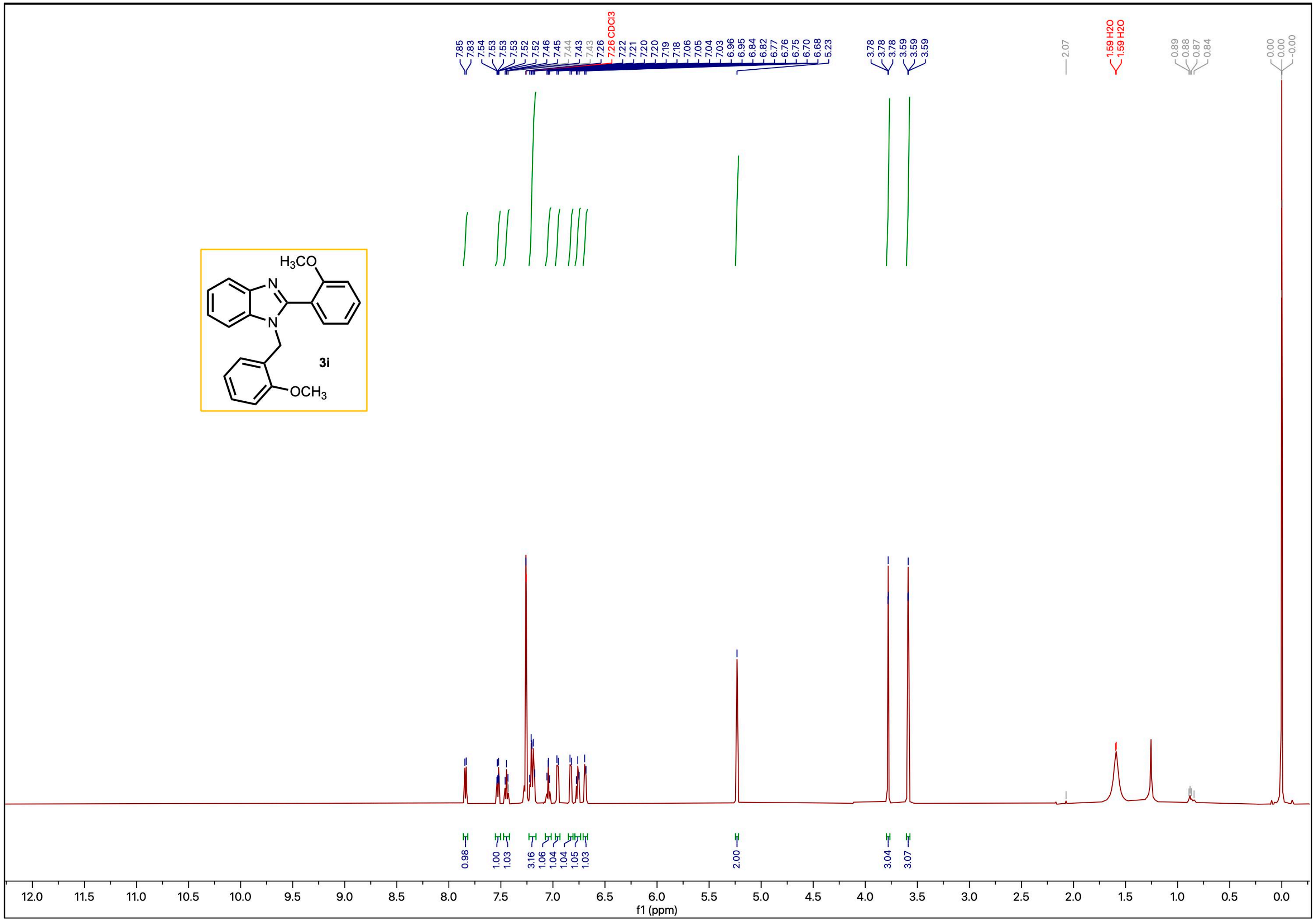
7.85
7.83
7.54
7.53
7.53
7.52
7.52
7.46
7.45
7.44
7.43
7.43
7.26
7.26 CDCl3
7.22
7.21
7.20
7.20
7.19
7.18
7.06
7.05
7.04
7.03
6.96
6.95
6.84
6.82
6.77
6.76
6.75
6.70
6.68
5.23
3.78
3.78
3.78
3.59
3.59
3.59
2.07
1.59 H2O
1.59 H2O
0.89
0.88
0.87
0.84
0.00
0.00
-0.00
H3CO
N
N
3i
OCH3
0.98
1.00
1.03
3.16
1.06
1.04
1.04
1.05
1.03
2.00
3.04
3.07
12.0
11.5
11.0
10.5
10.0
9.5
9.0
8.5
8.0
7.5
7.0
6.5
6.0
5.5
5.0
4.5
4.0
3.5
3.0
2.5
2.0
1.5
1.0
0.5
0.0
f1 (ppm)

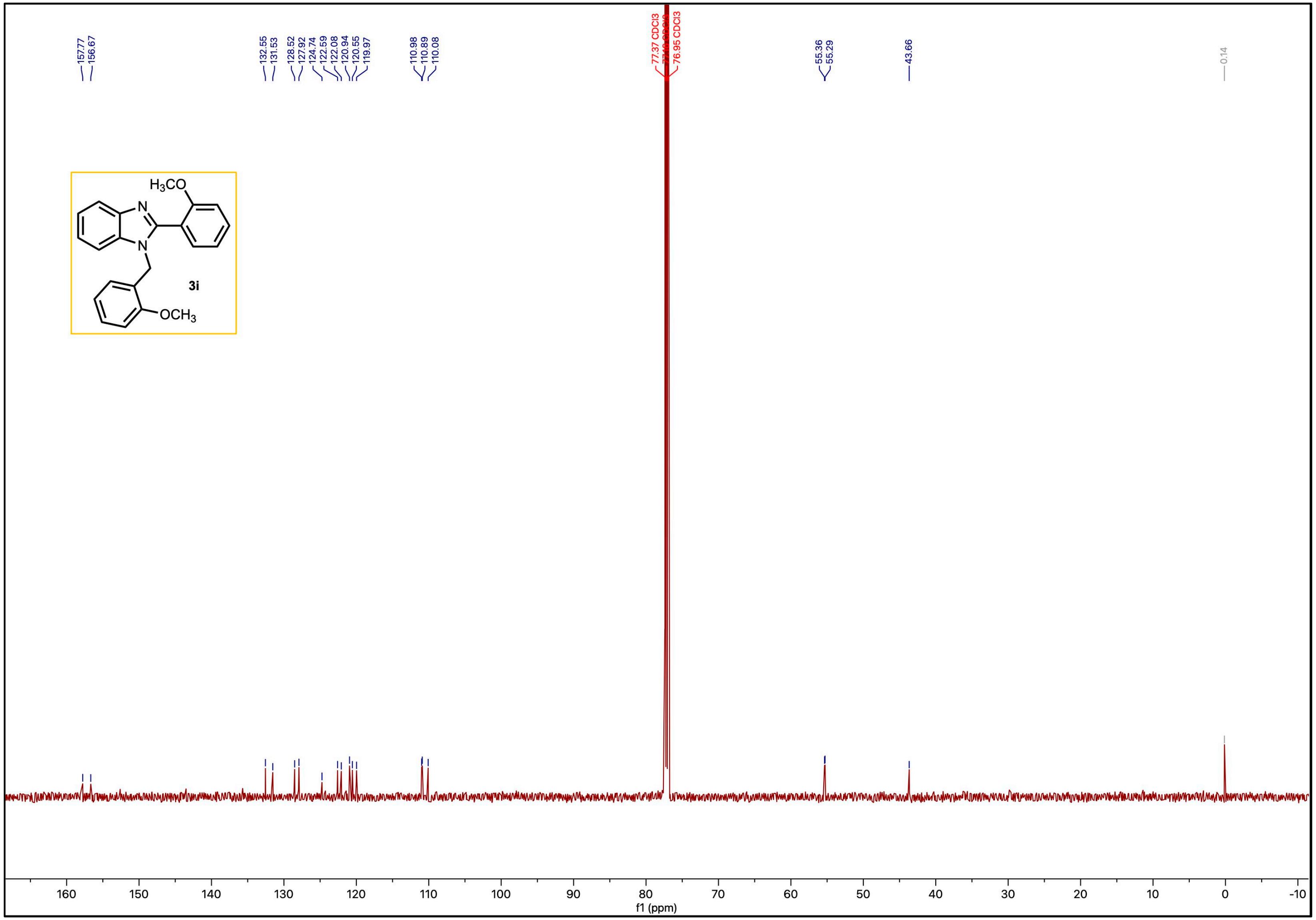
157.77
156.67
132.55
131.53
128.52
127.92
124.74
122.59
122.08
120.94
120.55
119.97
110.98
110.89
110.08
77.37 CDCl3
76.95 CDCl3
55.36
55.29
43.66
0.14
H3CO
N
N
3i
OCH3
f1 (ppm)

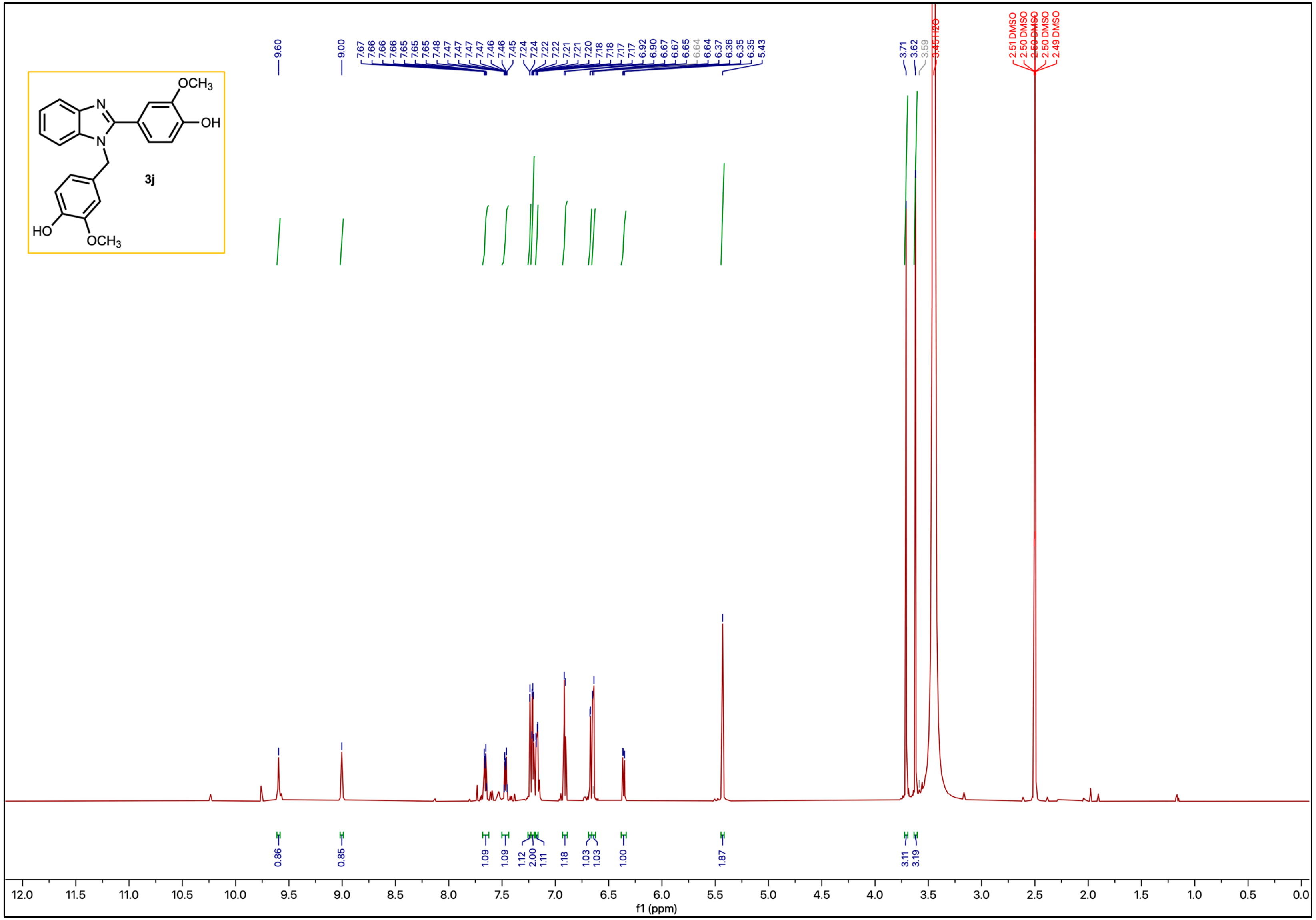

OCH3
OH
N
N
3j
HO
OCH3
f1 (ppm)

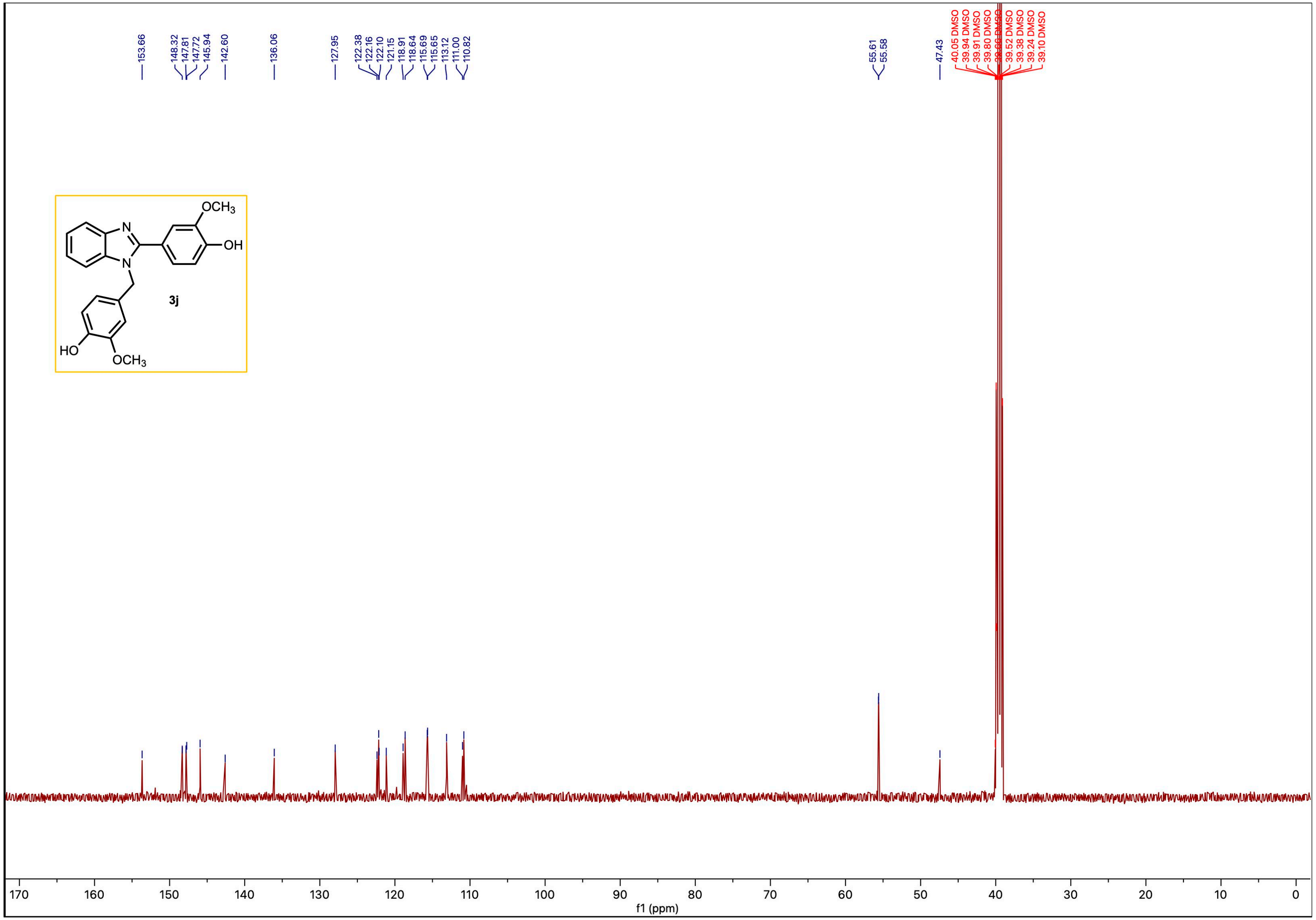
153.66
148.32
147.81
147.72
145.94
142.60
136.06
127.95
122.38
122.16
122.10
121.15
118.91
118.64
115.69
115.65
113.12
111.00
110.82
55.61
55.58
47.43
40.05 DMSO
39.94 DMSO
39.91 DMSO
39.80 DMSO
39.52 DMSO
39.38 DMSO
39.24 DMSO
39.10 DMSO
OCH3
N
N
OH
3j
HO
OCH3
f1 (ppm)

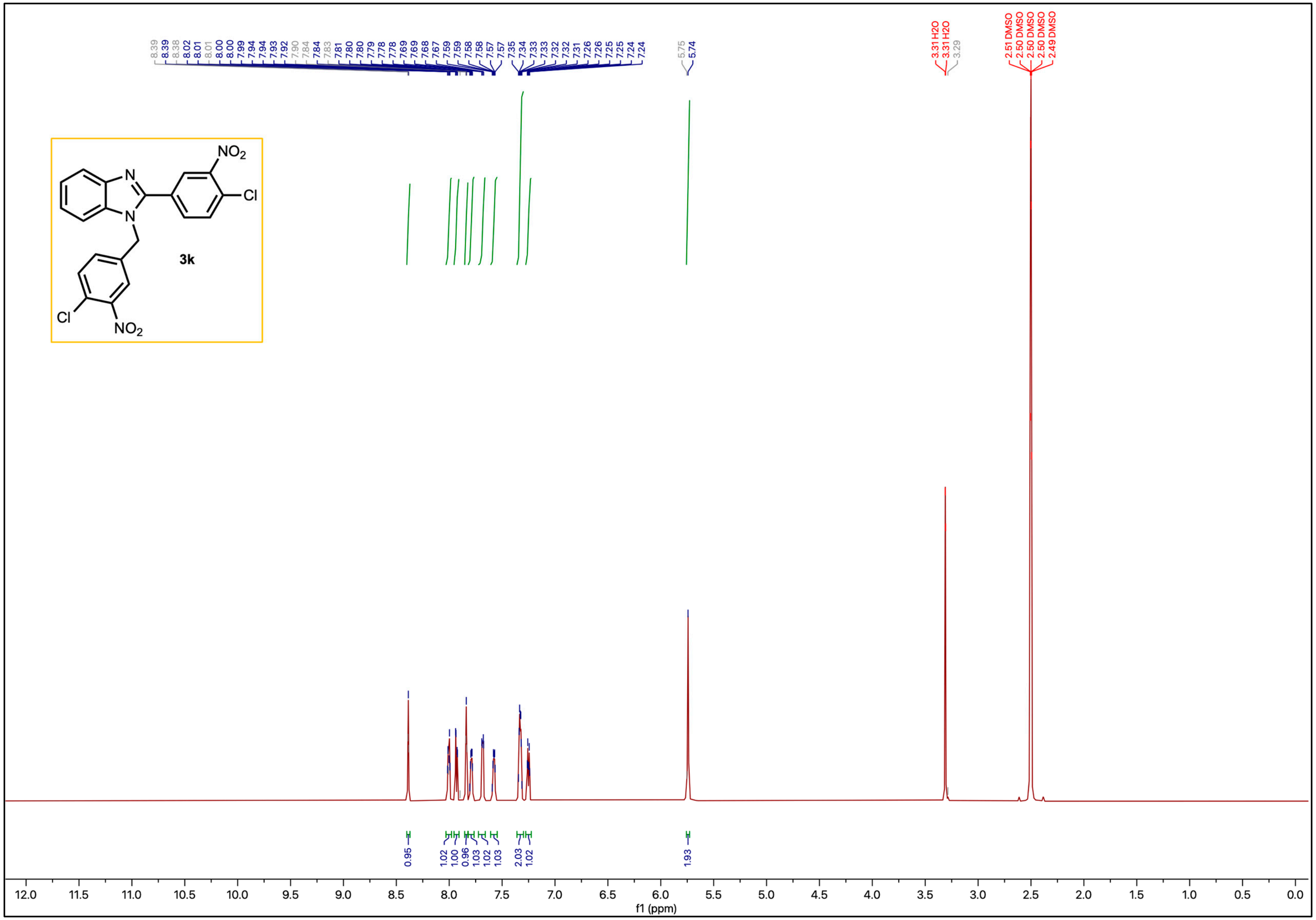
3k
f1 (ppm)

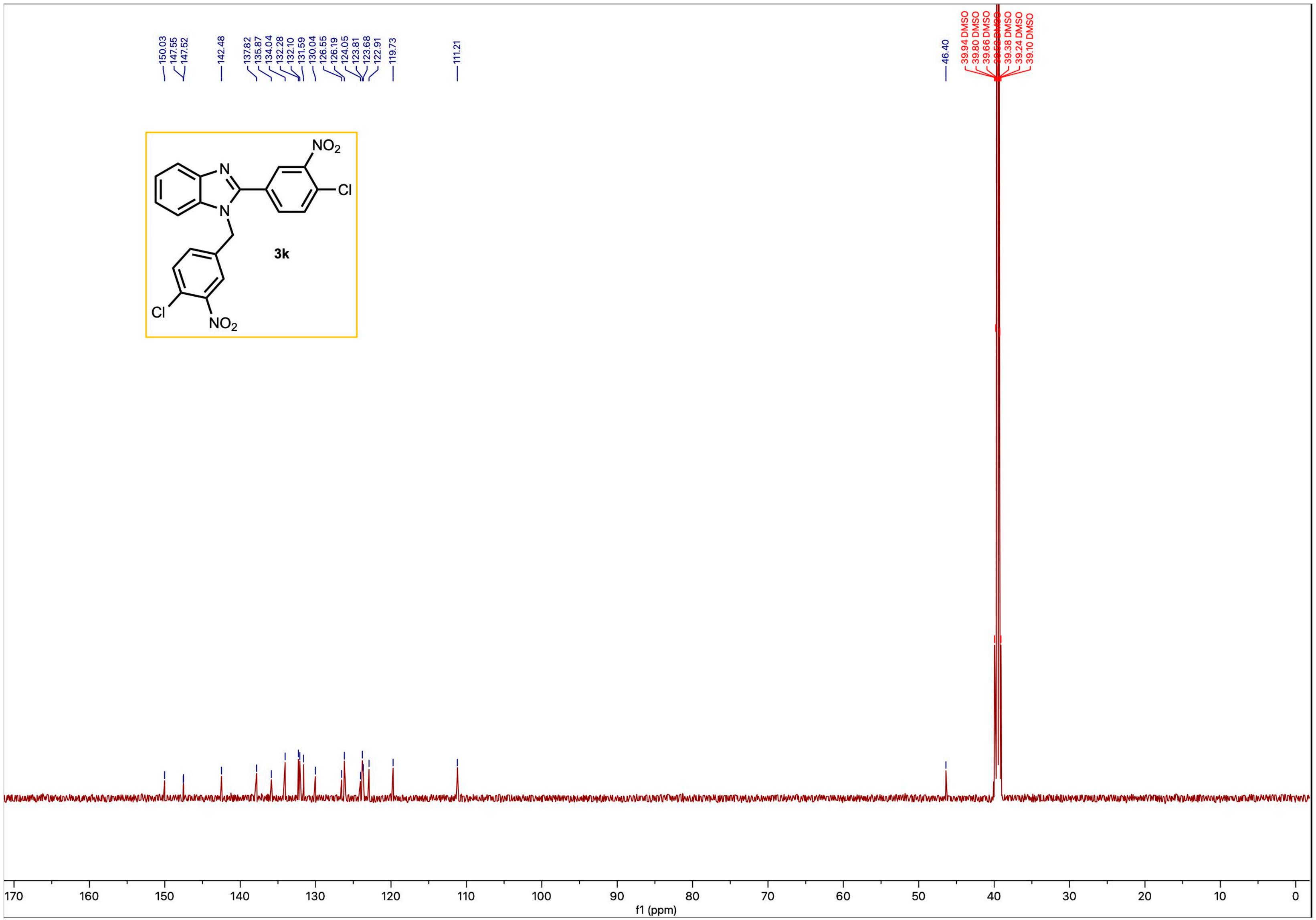
150.03
147.55
147.52
142.48
137.82
135.87
134.04
132.28
132.10
131.59
130.04
126.55
126.19
124.05
123.81
123.68
122.91
119.73
111.21
46.40
39.94 DMSO
39.80 DMSO
39.66 DMSO
39.38 DMSO
39.24 DMSO
39.10 DMSO
NO2
N
N
Cl
3k
Cl
NO2
f1 (ppm)

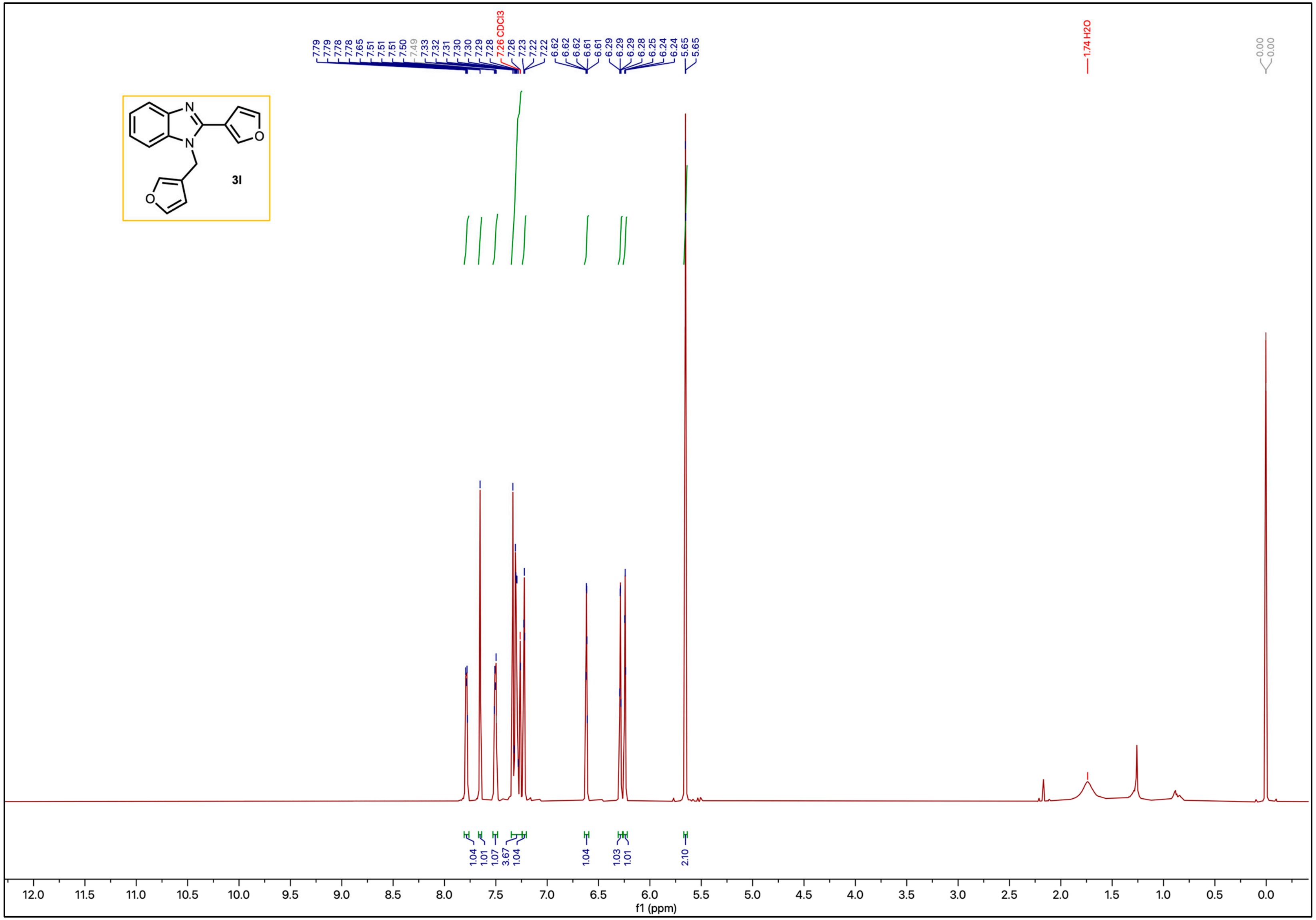
3l
f1 (ppm)
7.26 CDCl3
1.74 H2O

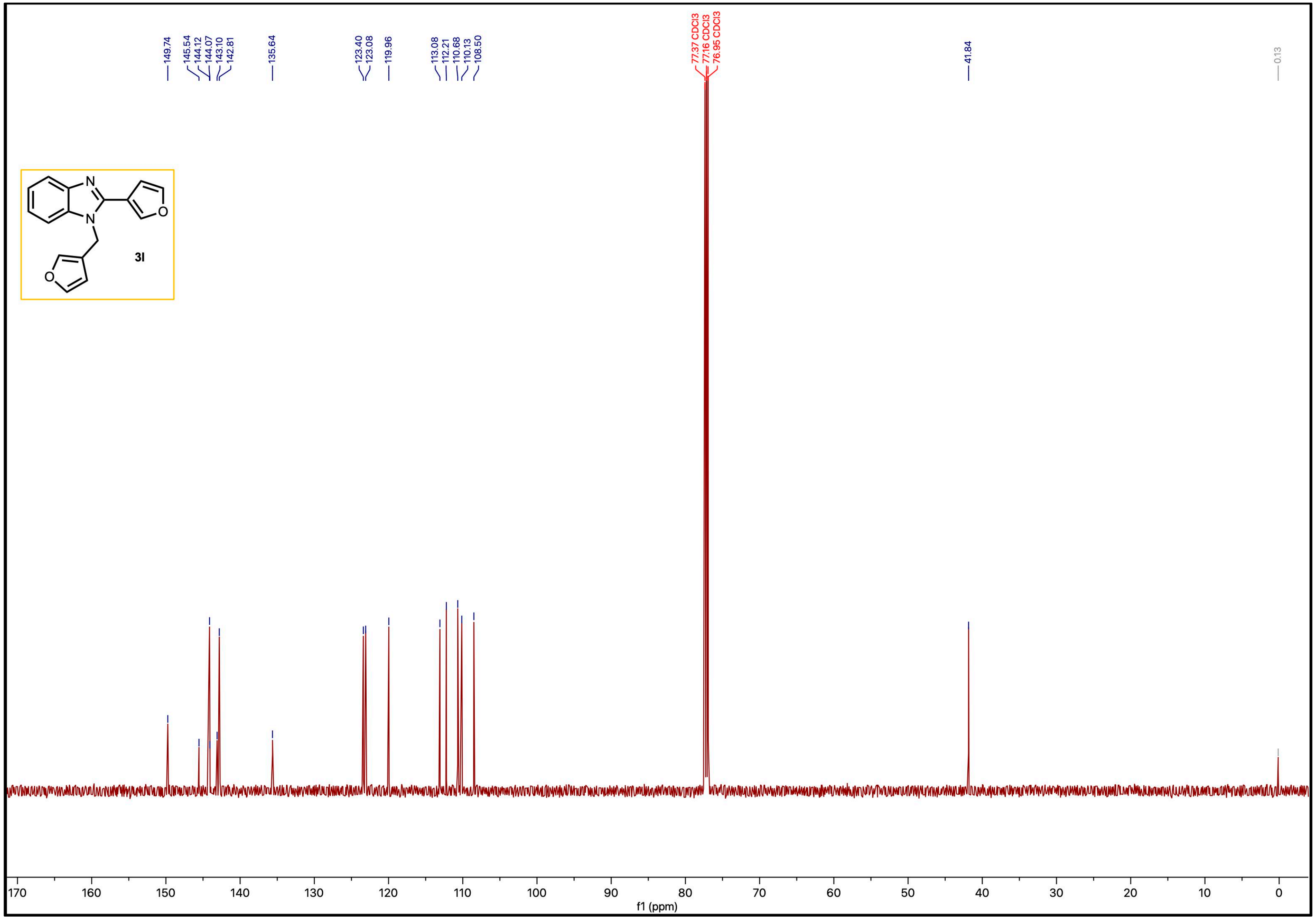

149.74
145.54
144.12
144.07
143.10
142.81
135.64
123.40
123.08
119.96
113.08
112.21
110.68
110.13
108.50
77.37 CDCl3
77.16 CDCl3
76.95 CDCl3
41.84
0.13
N
N
O
O
3l
170
160
150
140
130
120
110
100
90
80
70
60
50
40
30
20
10
0
f1 (ppm)

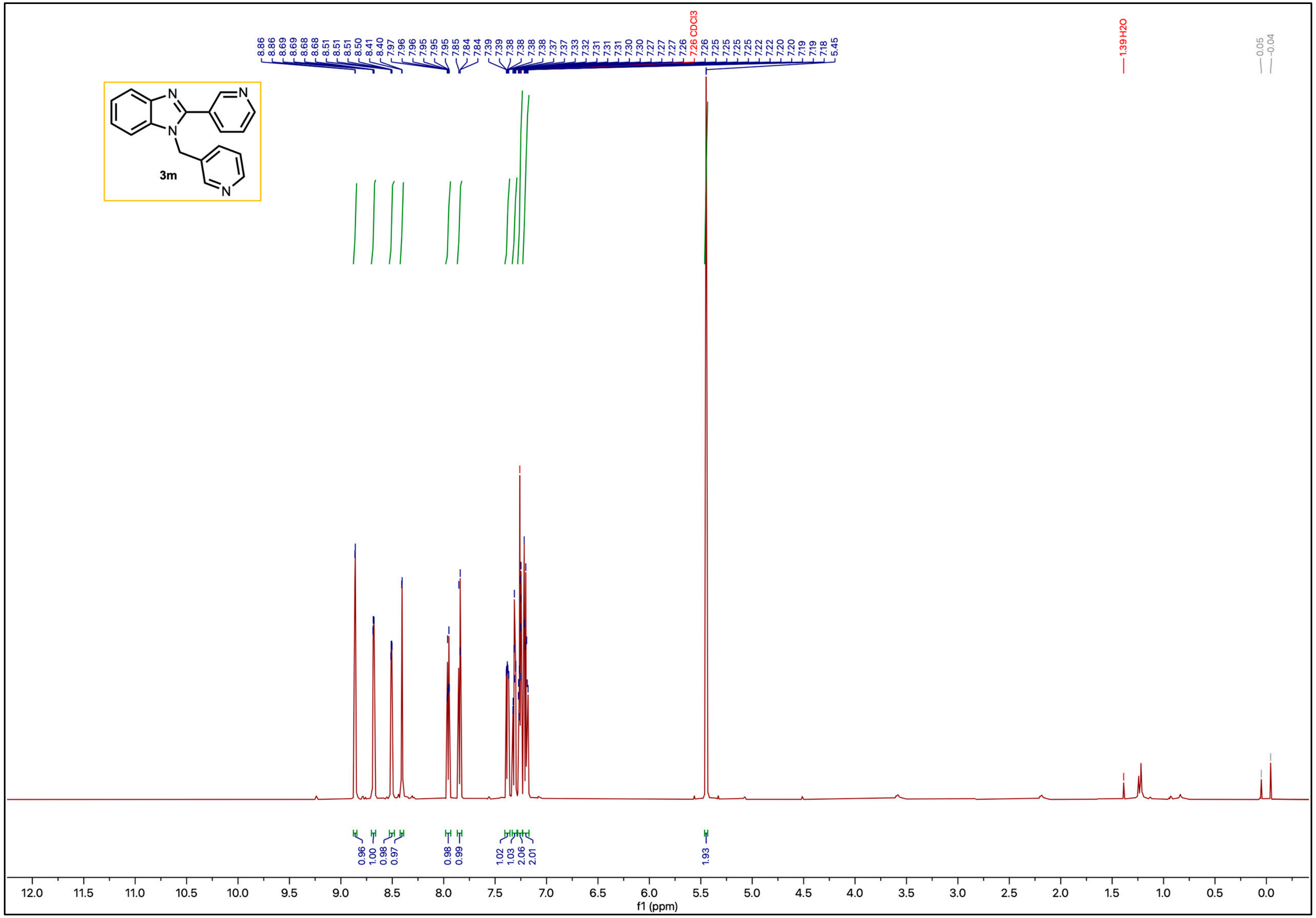

3m
N
N
N
8.86 8.86 8.69 8.69 8.68 8.68 8.51 8.51 8.51 8.50 8.41 8.40 7.97 7.96 7.96 7.95 7.95 7.95 7.85 7.84 7.84 7.39 7.39 7.38 7.38 7.38 7.38 7.37 7.37 7.33 7.32 7.31 7.31 7.31 7.30 7.30 7.27 7.27 7.27 7.26 7.26 CDCl3 7.26 7.25 7.25 7.25 7.25 7.25 7.22 7.22 7.20 7.20 7.19 7.19 7.18 5.45
1.39 H2O
0.05
-0.04
0.96 1.00 0.98 0.97 0.98 0.99 1.02 1.03 2.06 2.01 1.93
12.0 11.5 11.0 10.5 10.0 9.5 9.0 8.5 8.0 7.5 7.0 6.5 6.0 5.5 5.0 4.5 4.0 3.5 3.0 2.5 2.0 1.5 1.0 0.5 0.0
f1 (ppm)

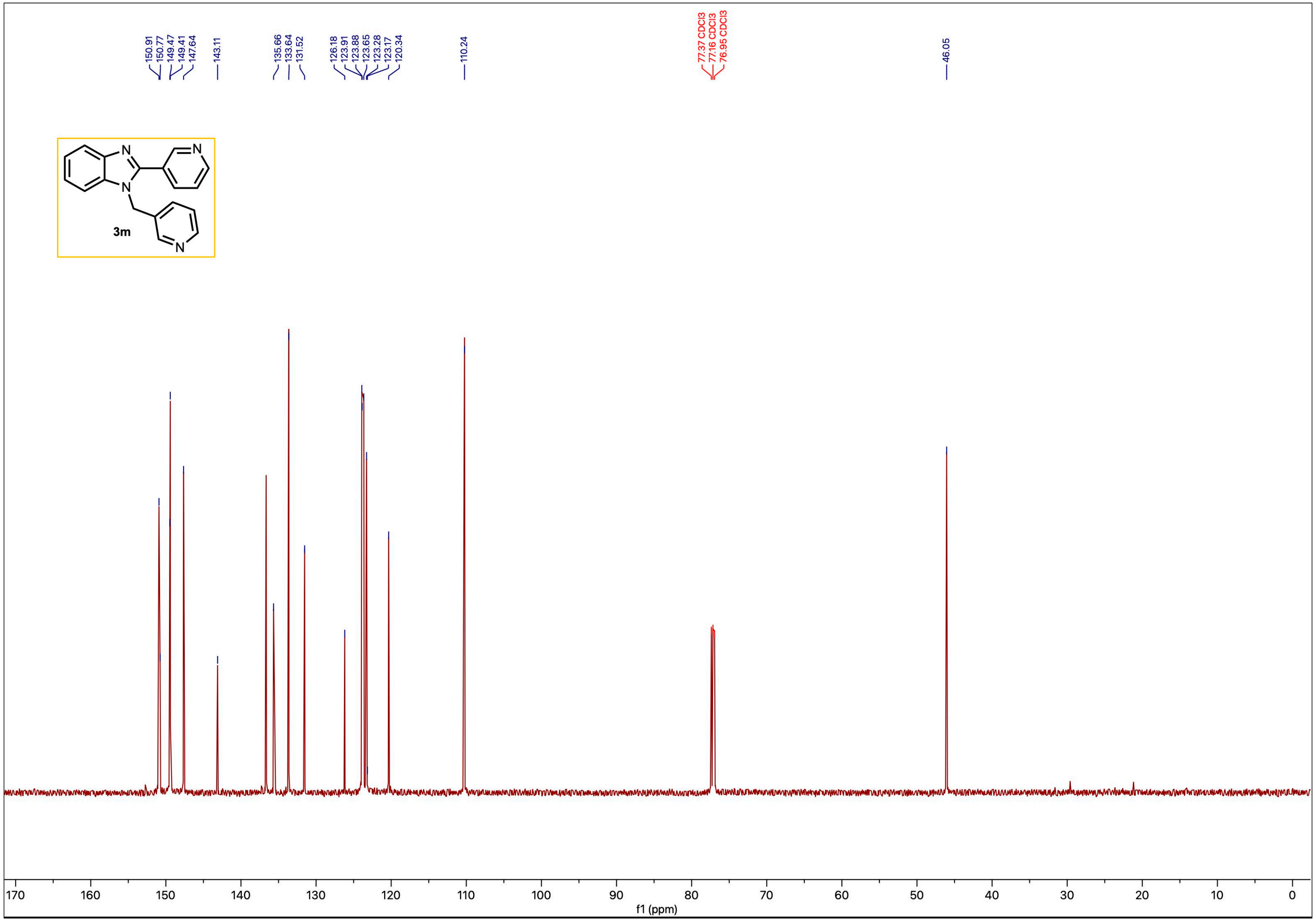
150.91
150.77
149.47
149.41
147.64
143.11
135.66
133.64
131.52
126.18
123.91
123.88
123.65
123.28
123.17
120.34
110.24
77.37 CDCl3
77.16 CDCl3
76.95 CDCl3
46.05
3m
170
160
150
140
130
120
110
100
90
80
70
60
50
40
30
20
10
0
f1 (ppm)

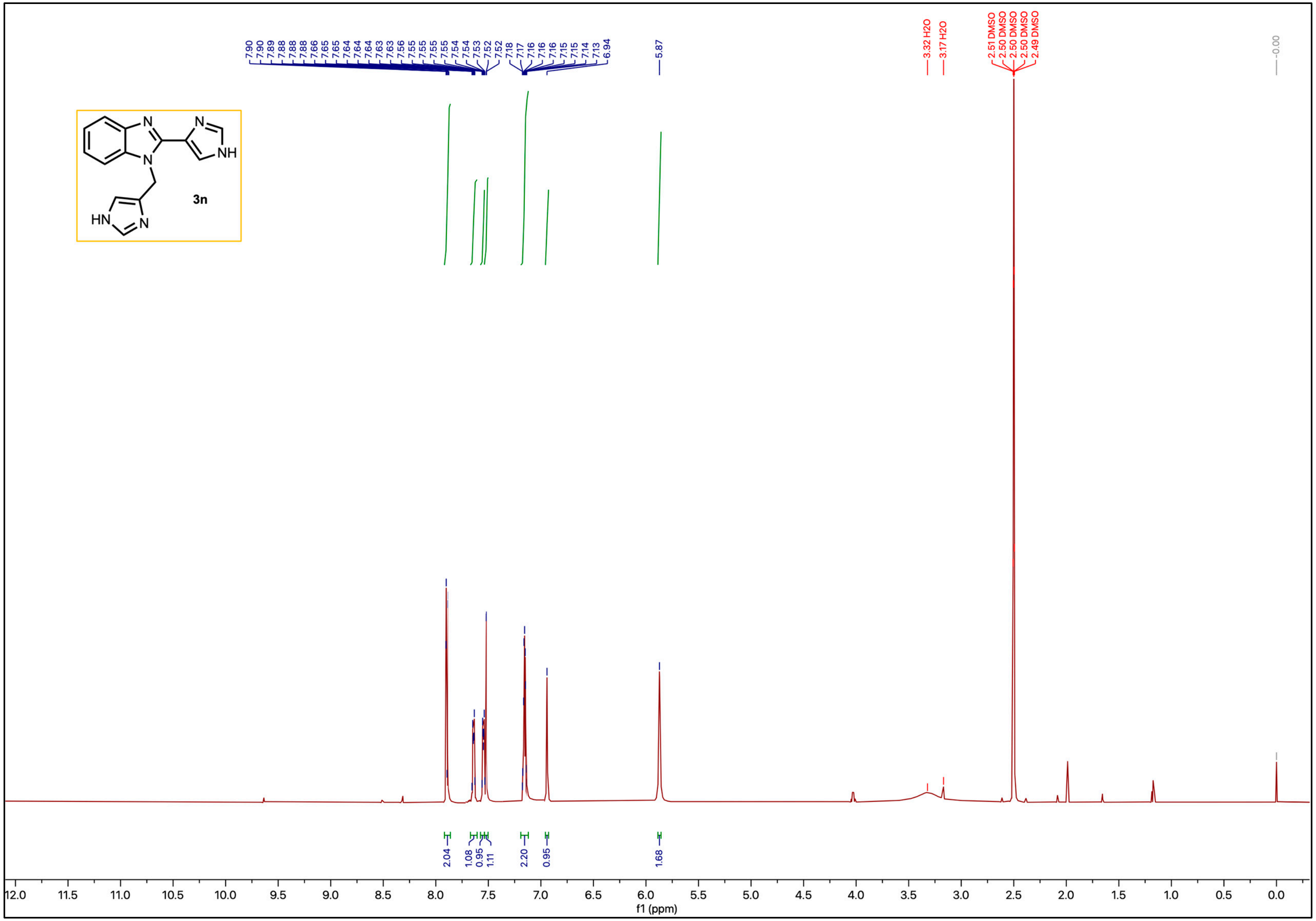

3n
HN
N
NH
f1 (ppm)
5.87
3.32 H2O
3.17 H2O
2.51 DMSO
2.50 DMSO
2.49 DMSO
-0.00
2.04
1.08
0.95
1.11
2.20
0.95
1.68

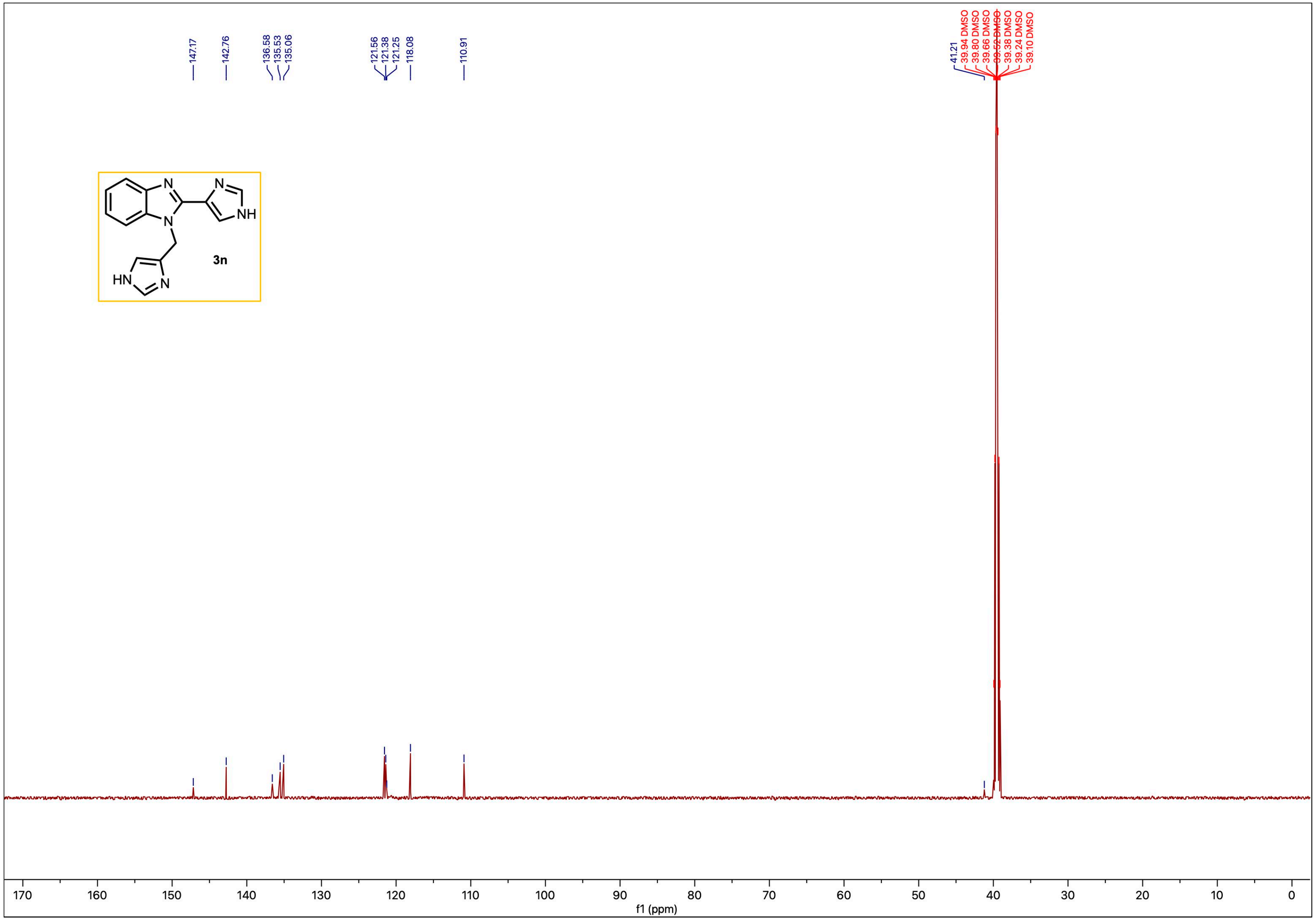
147.17
142.76
136.58
135.53
135.06
121.56
121.38
121.25
118.08
110.91
41.21
39.94 DMSO
39.80 DMSO
39.66 DMSO
39.52 DMSO
39.38 DMSO
39.24 DMSO
39.10 DMSO
N
N
NH
N
HN
N
3n
170
160
150
140
130
120
110
100
90
80
70
60
50
40
30
20
10
0
f1 (ppm)

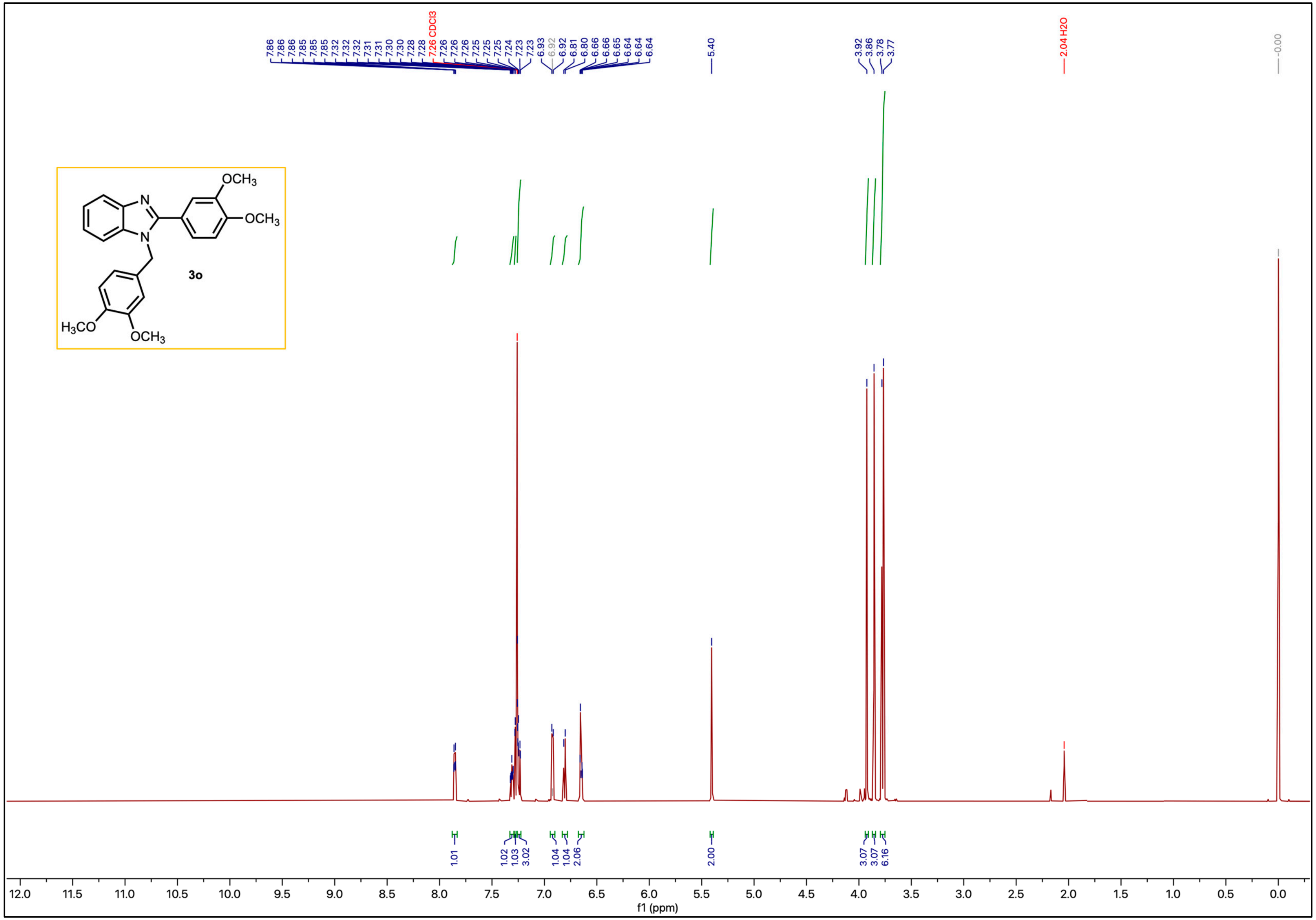

7.86 7.86 7.86 7.85 7.85 7.85 7.32 7.32 7.32 7.31 7.31 7.30 7.30 7.28 7.26 CDCl3 7.26 7.26 7.26 7.25 7.25 7.25 7.24 7.23 7.23 6.93 6.92 6.92 6.81 6.80 6.66 6.66 6.65 6.64 6.64 6.64
5.40
3.92 3.86 3.78 3.77
2.04 H2O
0.00
OCH3
N
OCH3
N
3o
H3CO
OCH3
1.01
1.02 1.03 3.02
1.04 1.04 2.06
2.00
3.07 3.07 6.16
12.0 11.5 11.0 10.5 10.0 9.5 9.0 8.5 8.0 7.5 7.0 6.5 6.0 5.5 5.0 4.5 4.0 3.5 3.0 2.5 2.0 1.5 1.0 0.5 0.0
f1 (ppm)

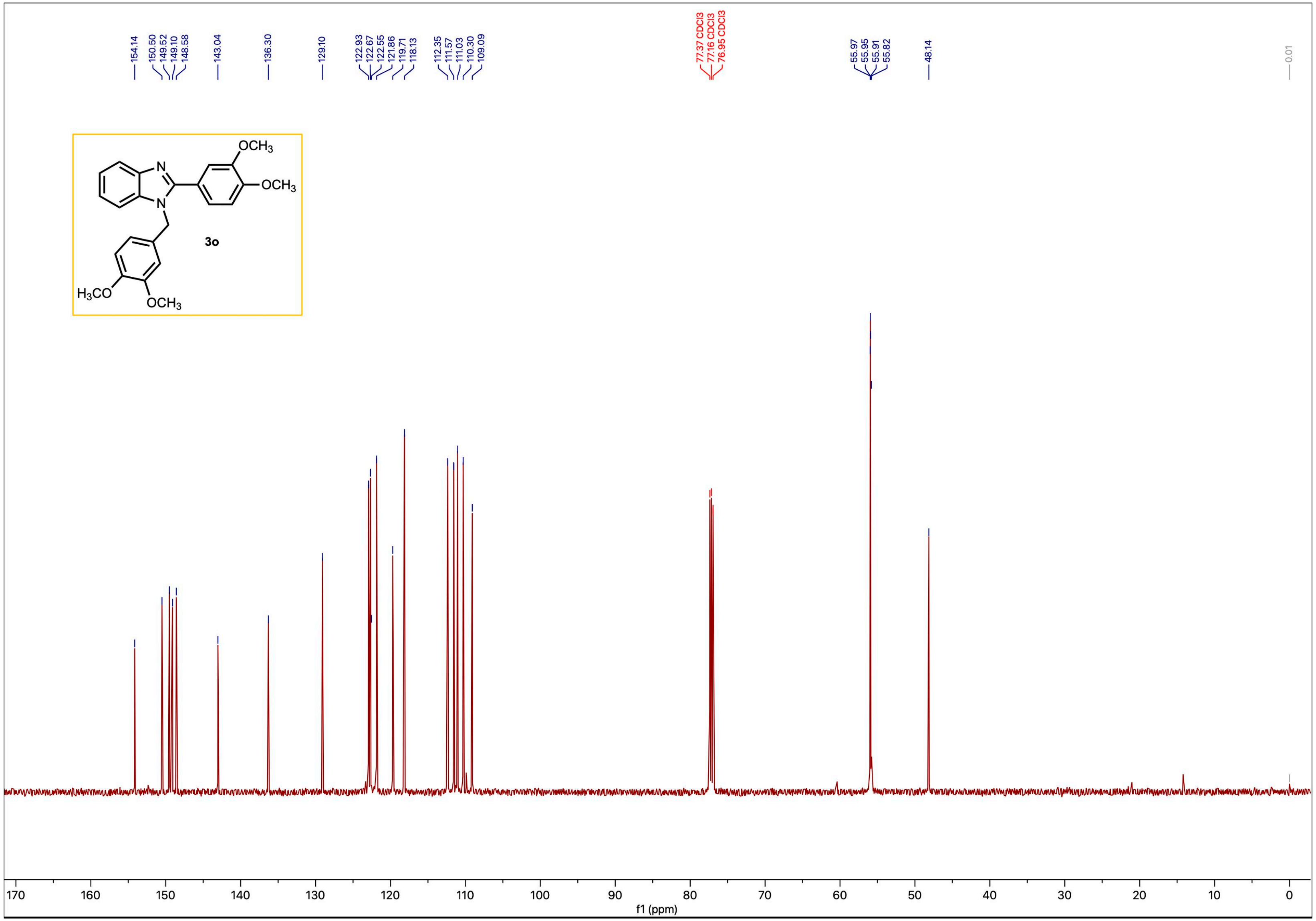
154.14
150.50
149.52
149.10
148.58
143.04
136.30
129.10
122.93
122.67
122.55
121.86
119.71
118.13
112.35
111.57
111.03
110.30
109.09
77.37 CDCl3
77.16 CDCl3
76.95 CDCl3
55.97
55.95
55.91
55.82
48.14
0.01
OCH3
N
OCH3
N
3o
H3CO
OCH3
f1 (ppm)

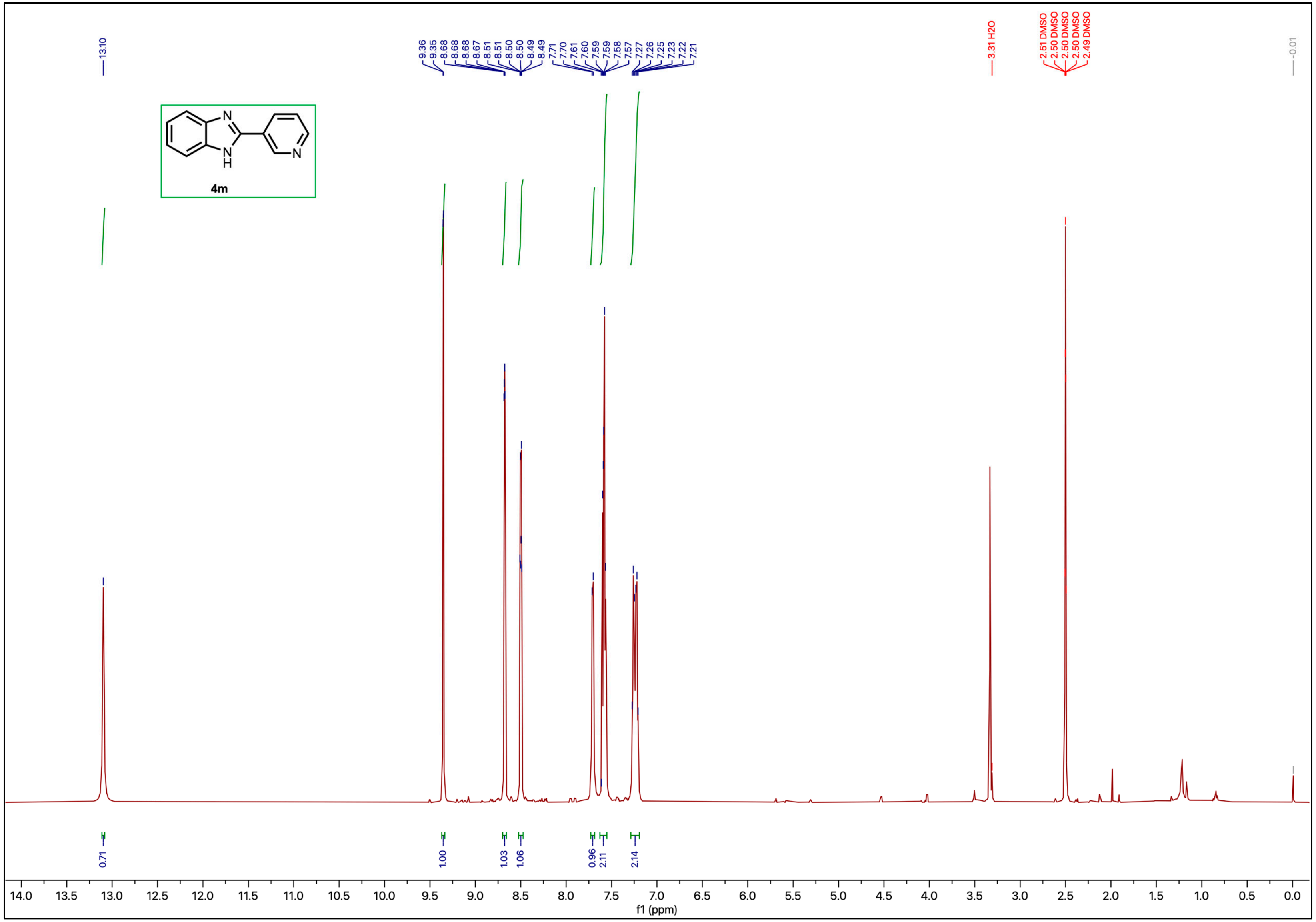

4m
13.10
9.36
9.35
8.68
8.68
8.68
8.67
8.51
8.51
8.50
8.50
8.49
8.49
7.71
7.70
7.61
7.60
7.59
7.59
7.58
7.57
7.27
7.26
7.25
7.23
7.22
7.21
3.31 H2O
2.51 DMSO
2.50 DMSO
2.50 DMSO
2.50 DMSO
2.49 DMSO
-0.01
0.71
1.00
1.03
1.06
0.96
2.11
2.14
14.0 13.5 13.0 12.5 12.0 11.5 11.0 10.5 10.0 9.5 9.0 8.5 8.0 7.5 7.0 6.5 6.0 5.5 5.0 4.5 4.0 3.5 3.0 2.5 2.0 1.5 1.0 0.5 0.0
f1 (ppm)

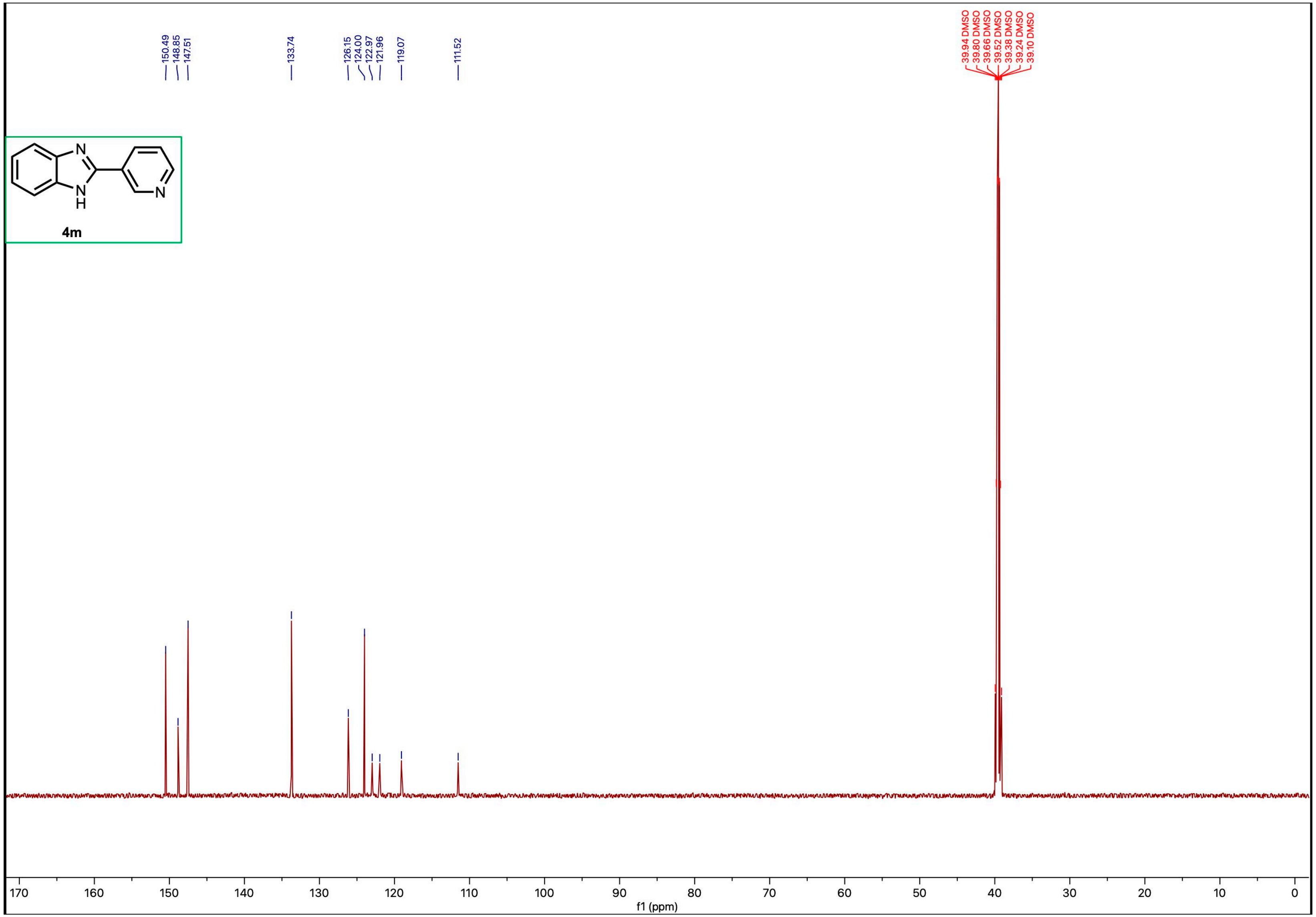
150.49
148.85
147.51
133.74
126.15
124.00
122.97
121.96
119.07
111.52
39.94 DMSO
39.80 DMSO
39.66 DMSO
39.52 DMSO
39.38 DMSO
39.24 DMSO
39.10 DMSO
N
N
H
N
4m
170
160
150
140
130
120
110
100
90
80
70
60
50
40
30
20
10
0
f1 (ppm)

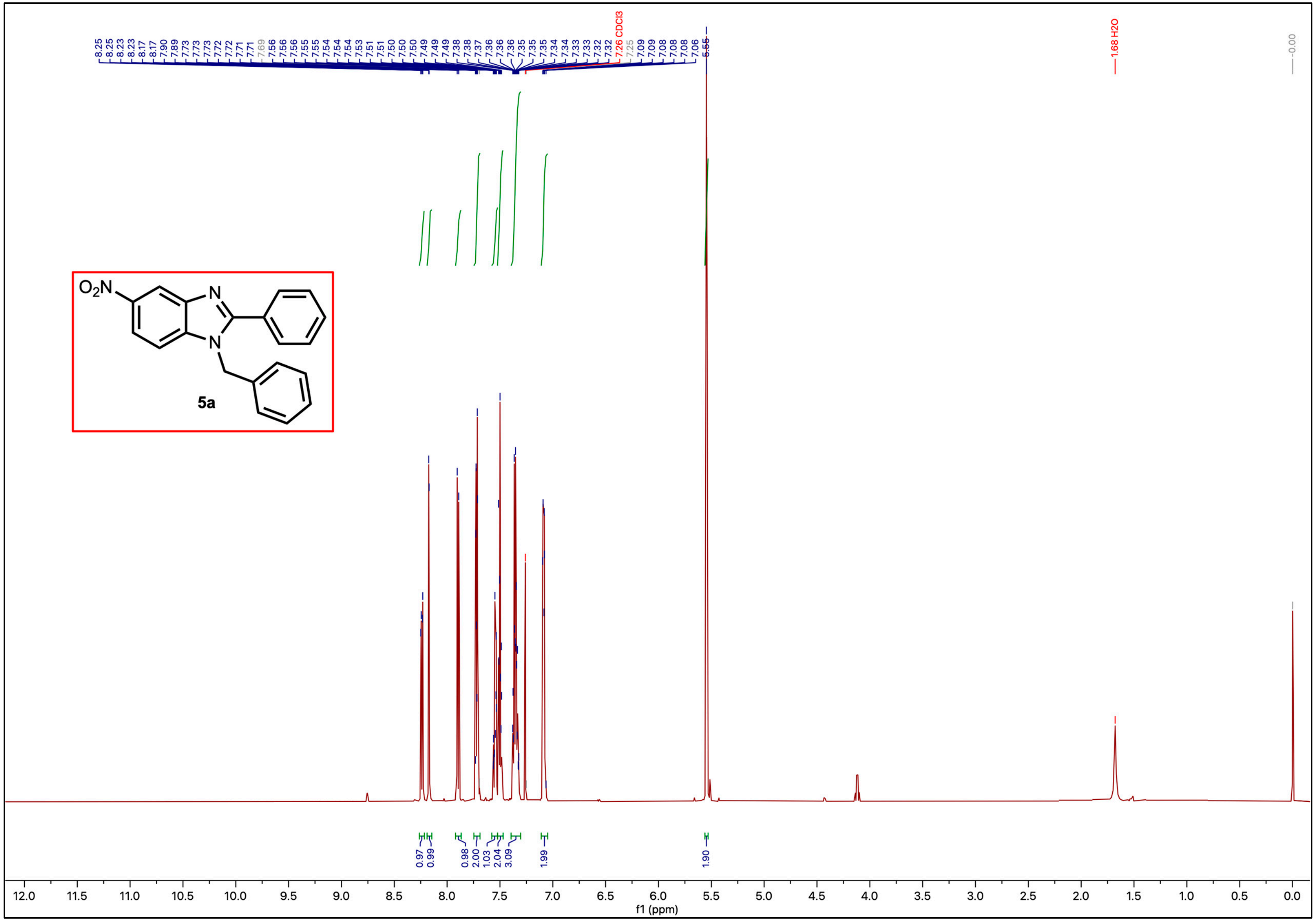

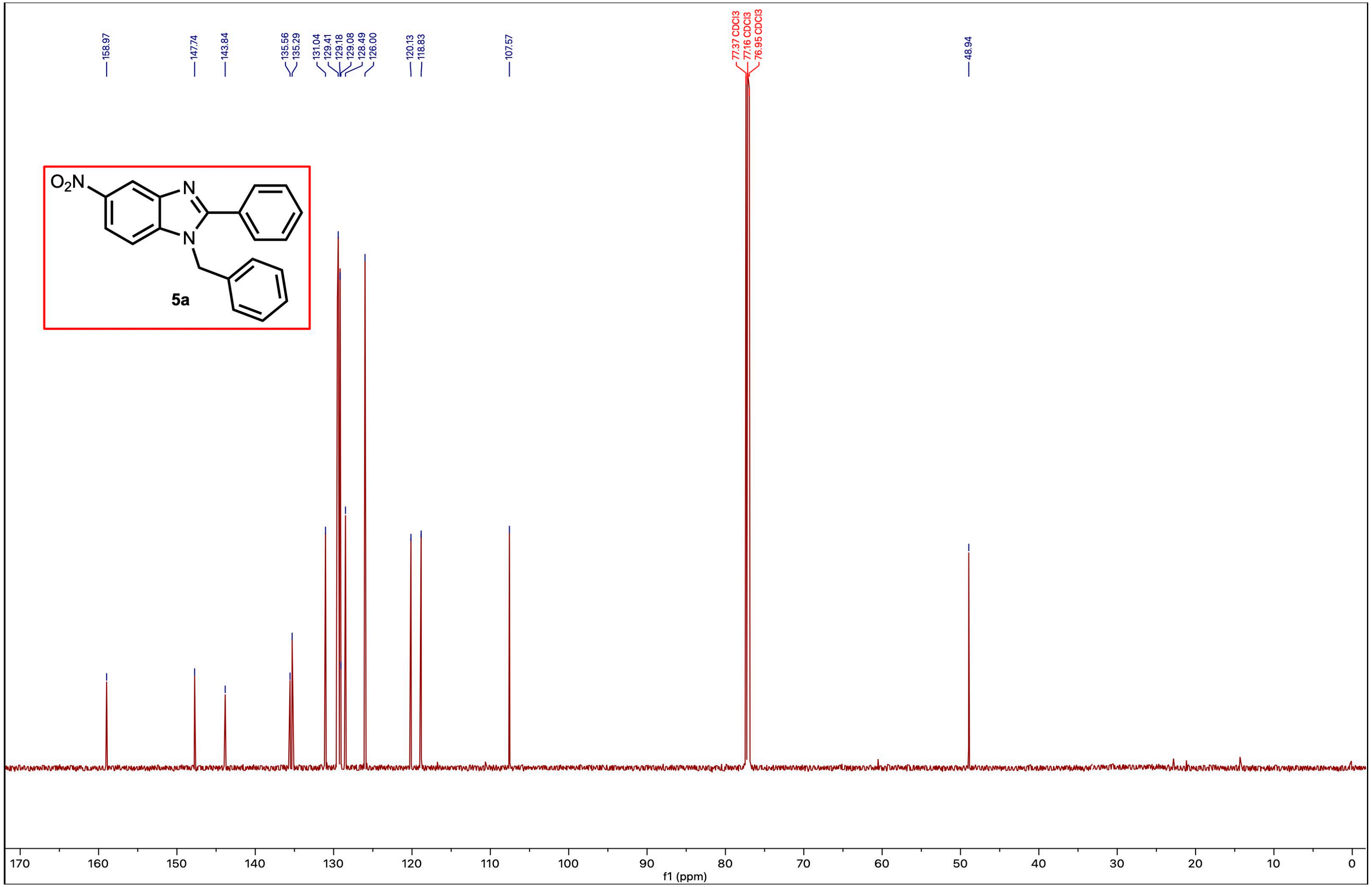
158.97
147.74
143.84
135.56
135.29
131.04
129.41
129.18
129.08
128.49
126.00
120.13
118.83
107.57
77.37 CDCl3
77.16 CDCl3
76.95 CDCl3
48.94
O2N
N
N
5a
170 160 150 140 130 120 110 100 90 80 70 60 50 40 30 20 10 0
f1 (ppm)

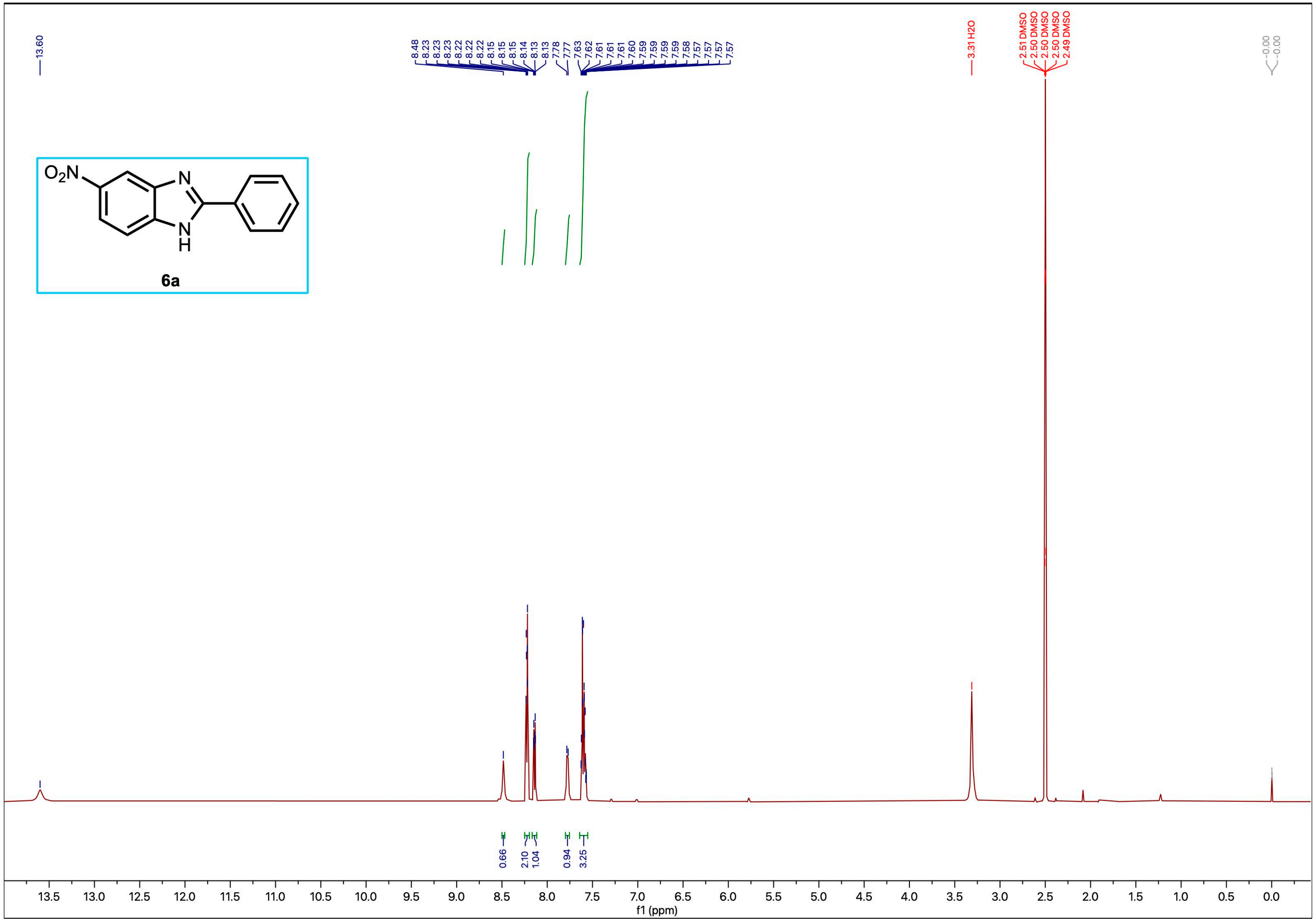
O2N
N
N
H
6a
13.60
8.48
8.23
8.23
8.23
8.22
8.22
8.22
8.15
8.15
8.15
8.14
8.13
8.13
7.78
7.77
7.63
7.62
7.61
7.61
7.61
7.60
7.59
7.59
7.59
7.59
7.58
7.57
7.57
7.57
3.31 H2O
2.51 DMSO
2.50 DMSO
2.50 DMSO
2.50 DMSO
2.49 DMSO
0.00
-0.00
0.66
2.10
1.04
0.94
3.25
f1 (ppm)

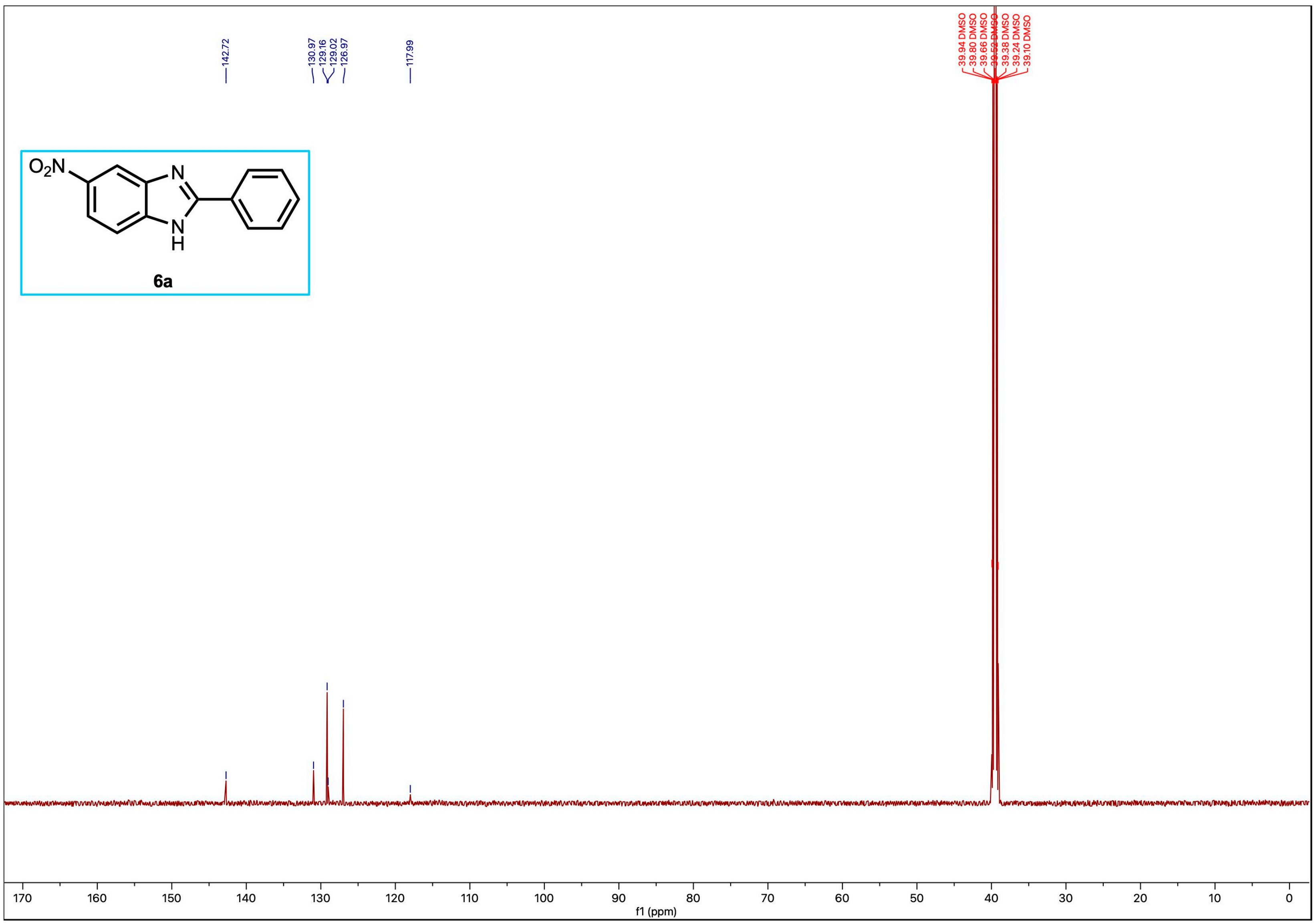
142.72
130.97
129.16
129.02
126.97
117.99
39.94 DMSO
39.80 DMSO
39.66 DMSO
39.38 DMSO
39.24 DMSO
39.10 DMSO
O2N
N
N
H
6a
170
160
150
140
130
120
110
100
90
80
70
60
50
40
30
20
10
0
f1 (ppm)

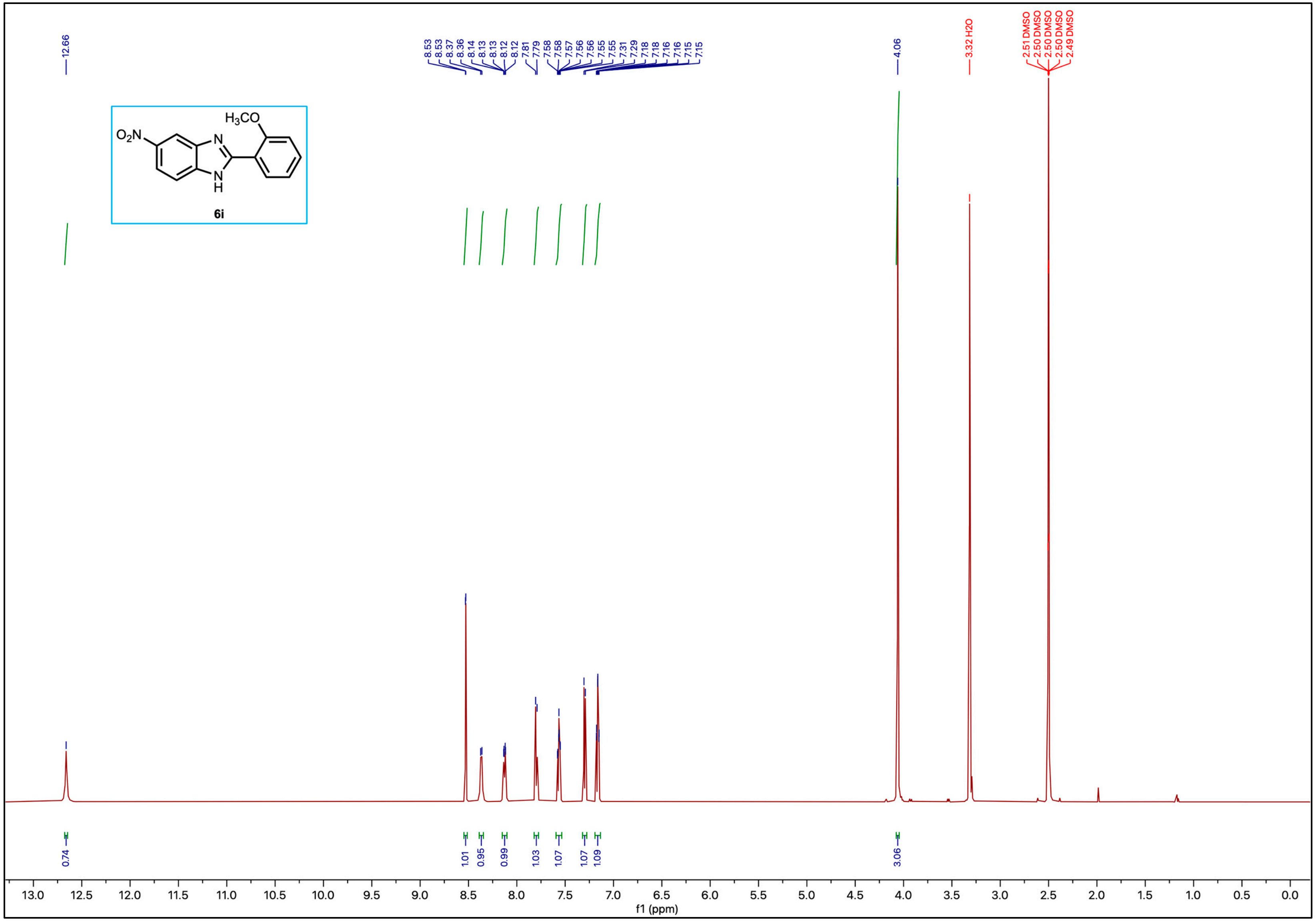
12.66
8.53
8.53
8.37
8.36
8.14
8.13
8.13
8.12
8.12
7.81
7.79
7.58
7.57
7.56
7.56
7.55
7.55
7.31
7.29
7.18
7.18
7.16
7.16
7.15
7.15
4.06
3.32 H2O
2.51 DMSO
2.50 DMSO
2.50 DMSO
2.50 DMSO
2.49 DMSO
H3CO
O2N
N
N
H
6i
0.74
1.01
0.95
0.99
1.03
1.07
1.07
1.09
3.06
f1 (ppm)

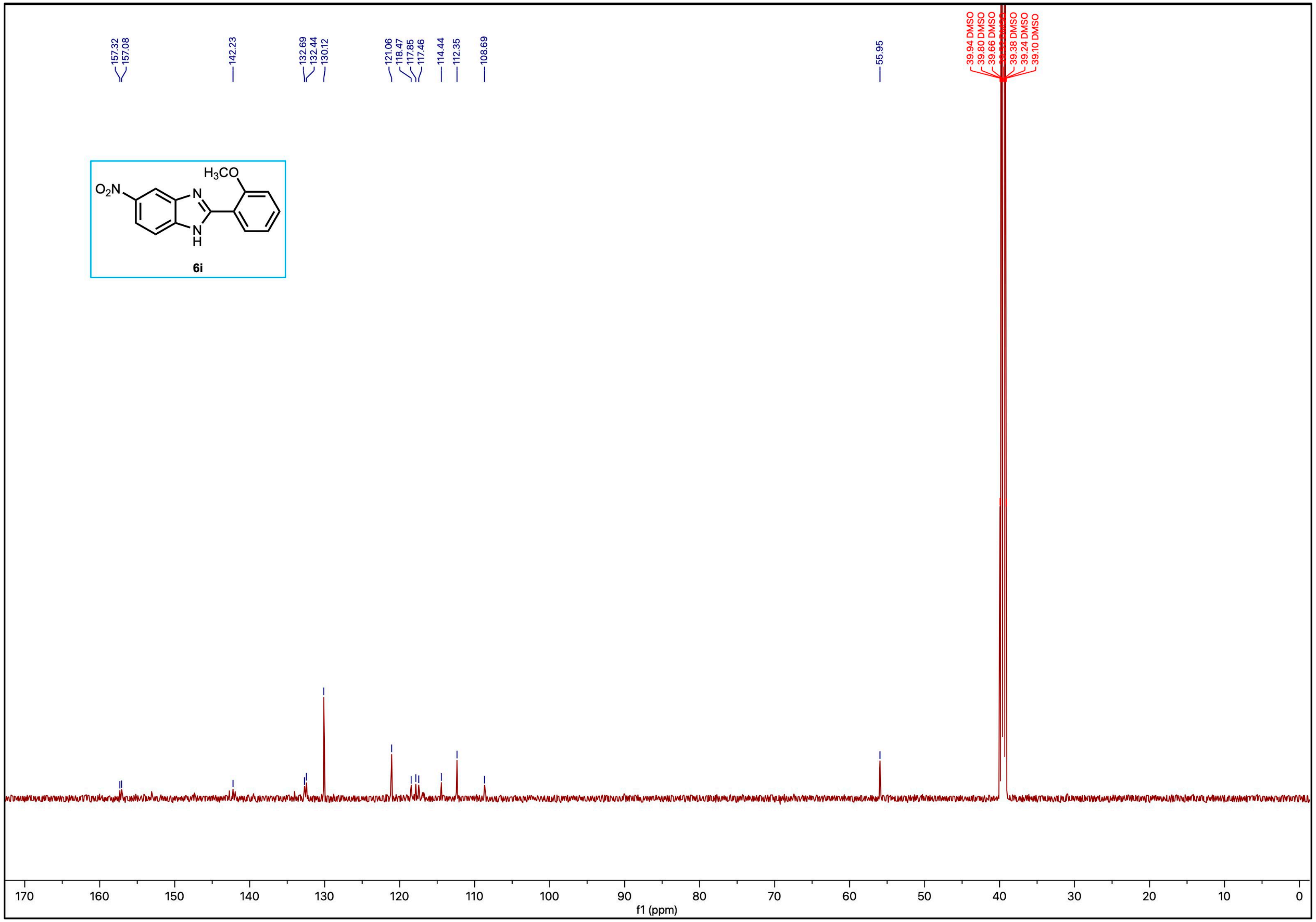

157.32
157.08
142.23
132.69
132.44
130.12
121.06
118.47
117.85
117.46
114.44
112.35
108.69
55.95
39.94 DMSO
39.80 DMSO
39.66 DMSO
39.38 DMSO
39.24 DMSO
39.10 DMSO
H3CO
O2N
N
N
H
6i
170
160
150
140
130
120
110
100
90
80
70
60
50
40
30
20
10
0
f1 (ppm)

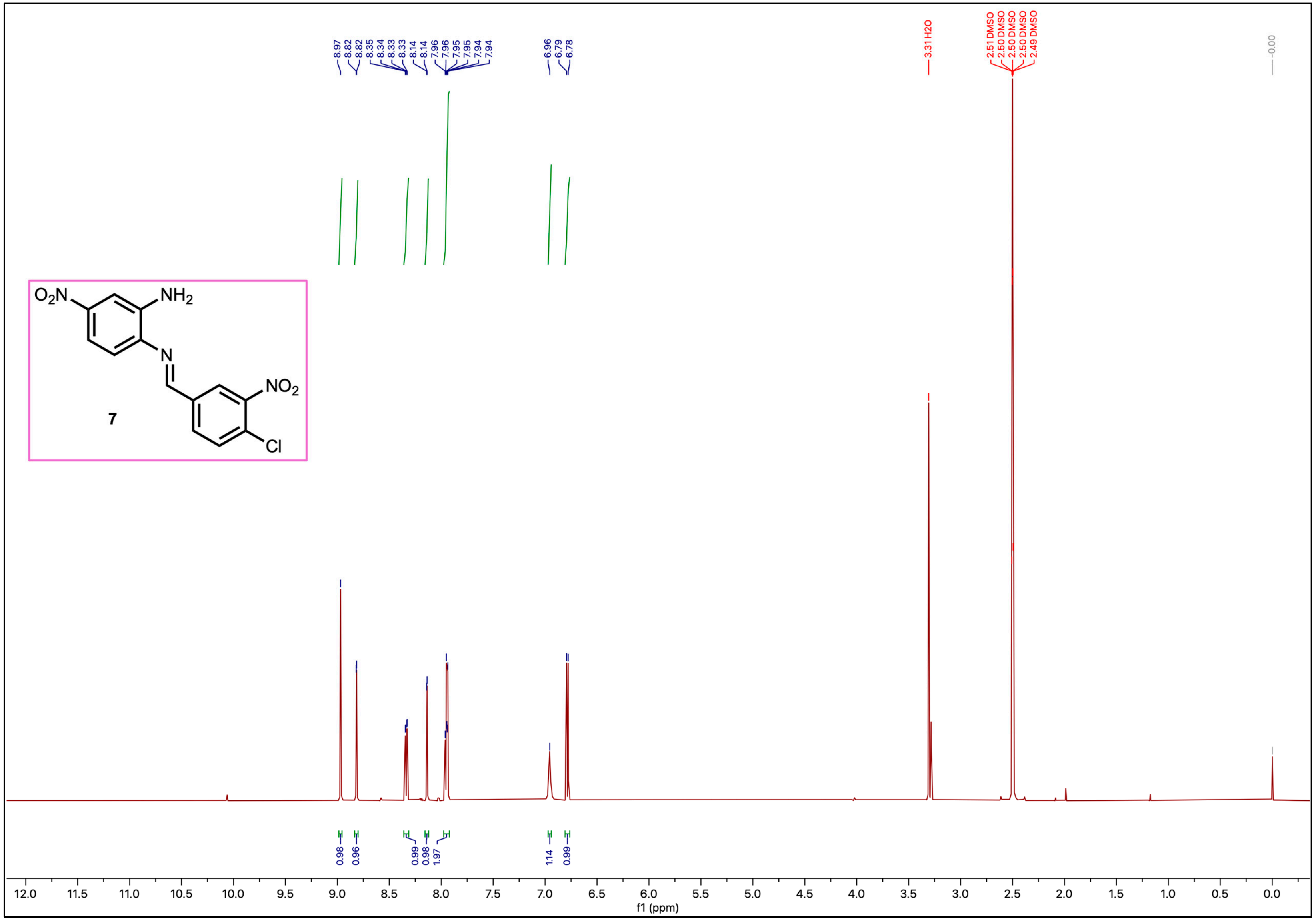

8.97
8.82
8.82
8.35
8.34
8.33
8.33
8.14
8.14
7.96
7.96
7.95
7.95
7.94
7.94
6.96
6.79
6.78
3.31 H2O
2.51 DMSO
2.50 DMSO
2.50 DMSO
2.50 DMSO
2.49 DMSO
0.00
0.98
0.96
0.99
0.98
1.97
1.14
0.99
12.0 11.5 11.0 10.5 10.0 9.5 9.0 8.5 8.0 7.5 7.0 6.5 6.0 5.5 5.0 4.5 4.0 3.5 3.0 2.5 2.0 1.5 1.0 0.5 0.0
f1 (ppm)
O2N
NH2
N
NO2
Cl
7

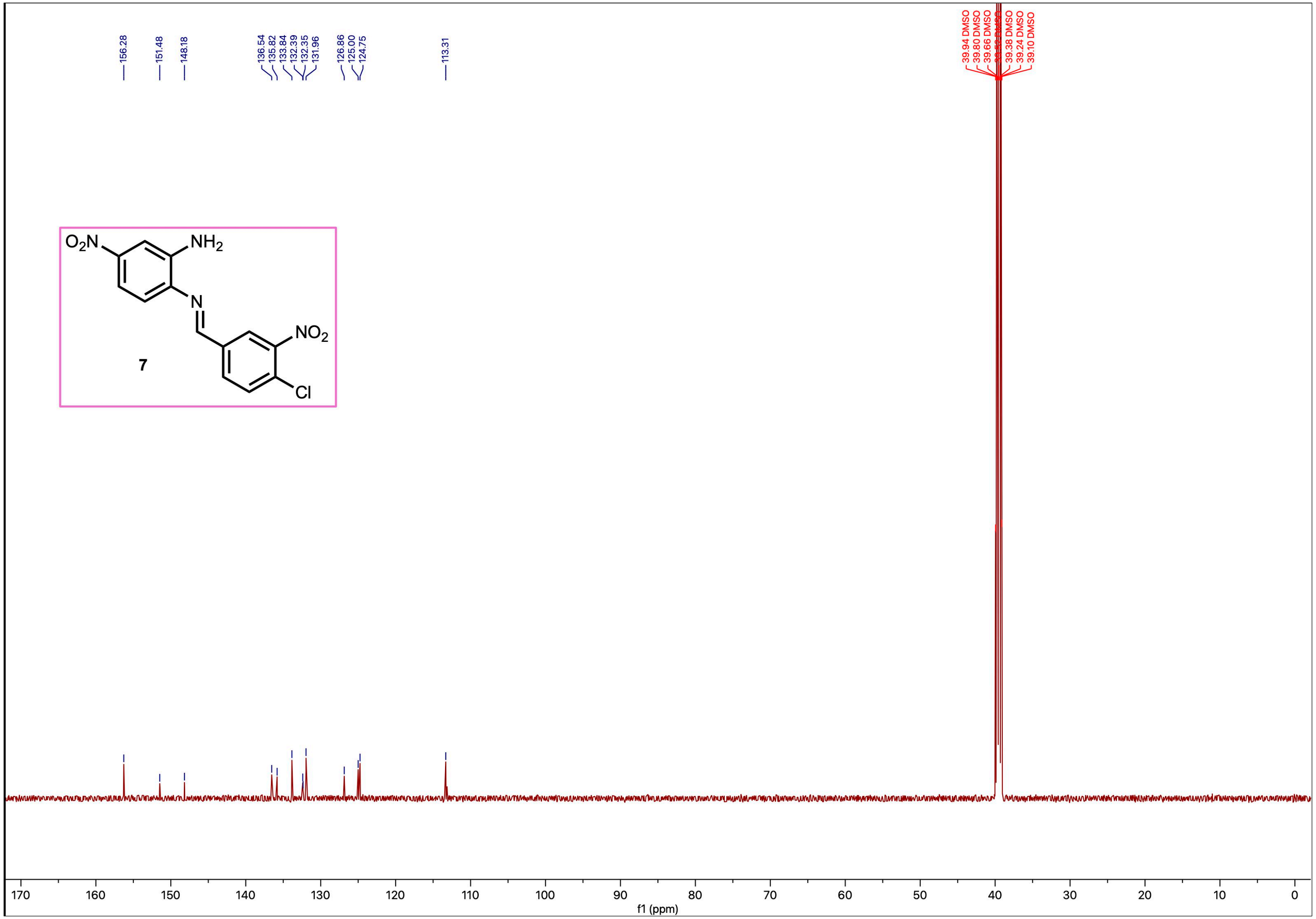
156.28
151.48
148.18
136.54
135.82
133.84
132.39
132.35
131.96
126.86
125.00
124.75
113.31
39.94 DMSO
39.80 DMSO
39.66 DMSO
39.38 DMSO
39.24 DMSO
39.10 DMSO
7
f1 (ppm)

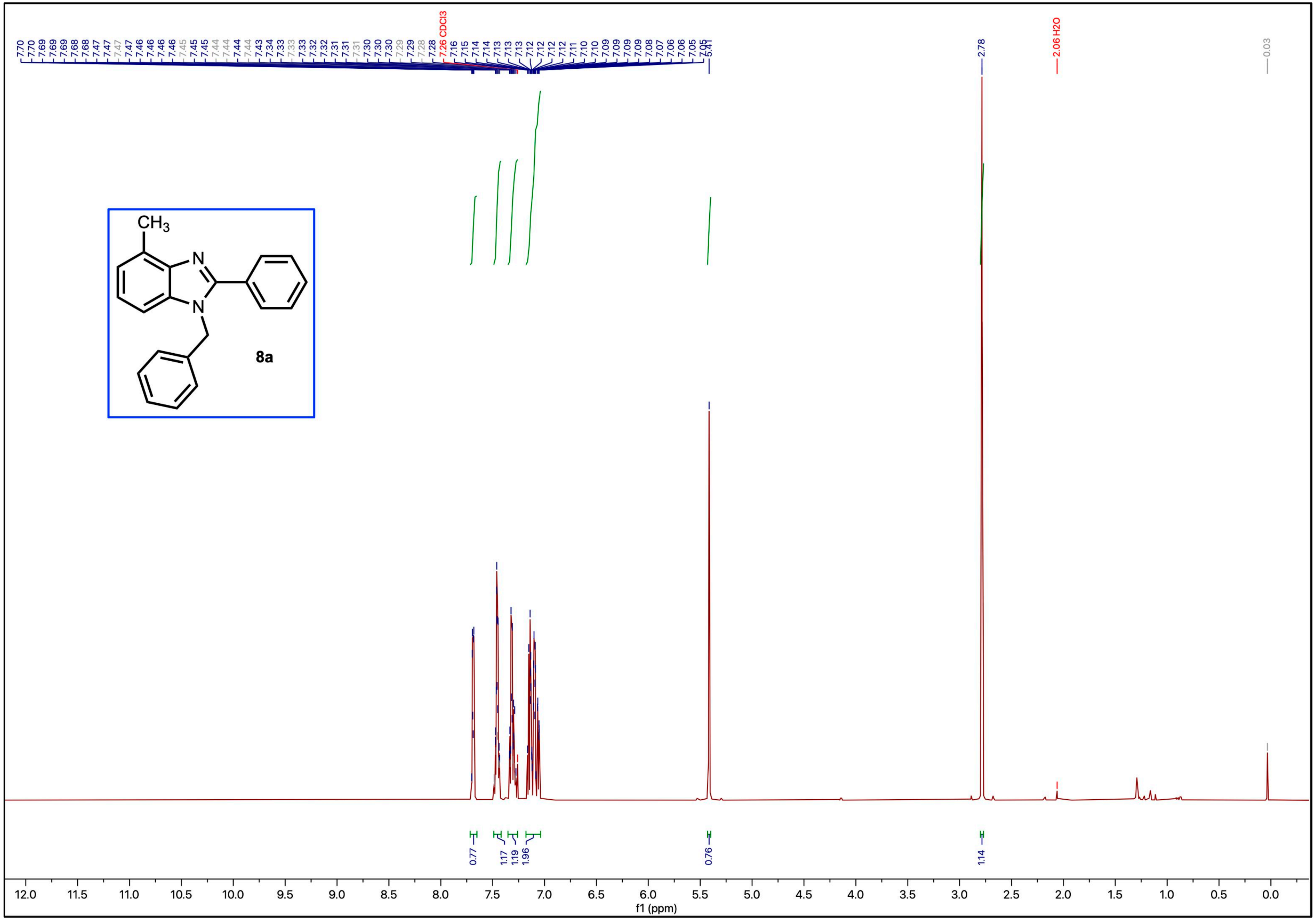

CH3
N
N
8a
7.26 CDCl3
2.78
2.06 H2O
0.03
5.41
0.77
1.17
1.19
1.96
0.76
1.14
12.0 11.5 11.0 10.5 10.0 9.5 9.0 8.5 8.0 7.5 7.0 6.5 6.0 5.5 5.0 4.5 4.0 3.5 3.0 2.5 2.0 1.5 1.0 0.5 0.0
f1 (ppm)

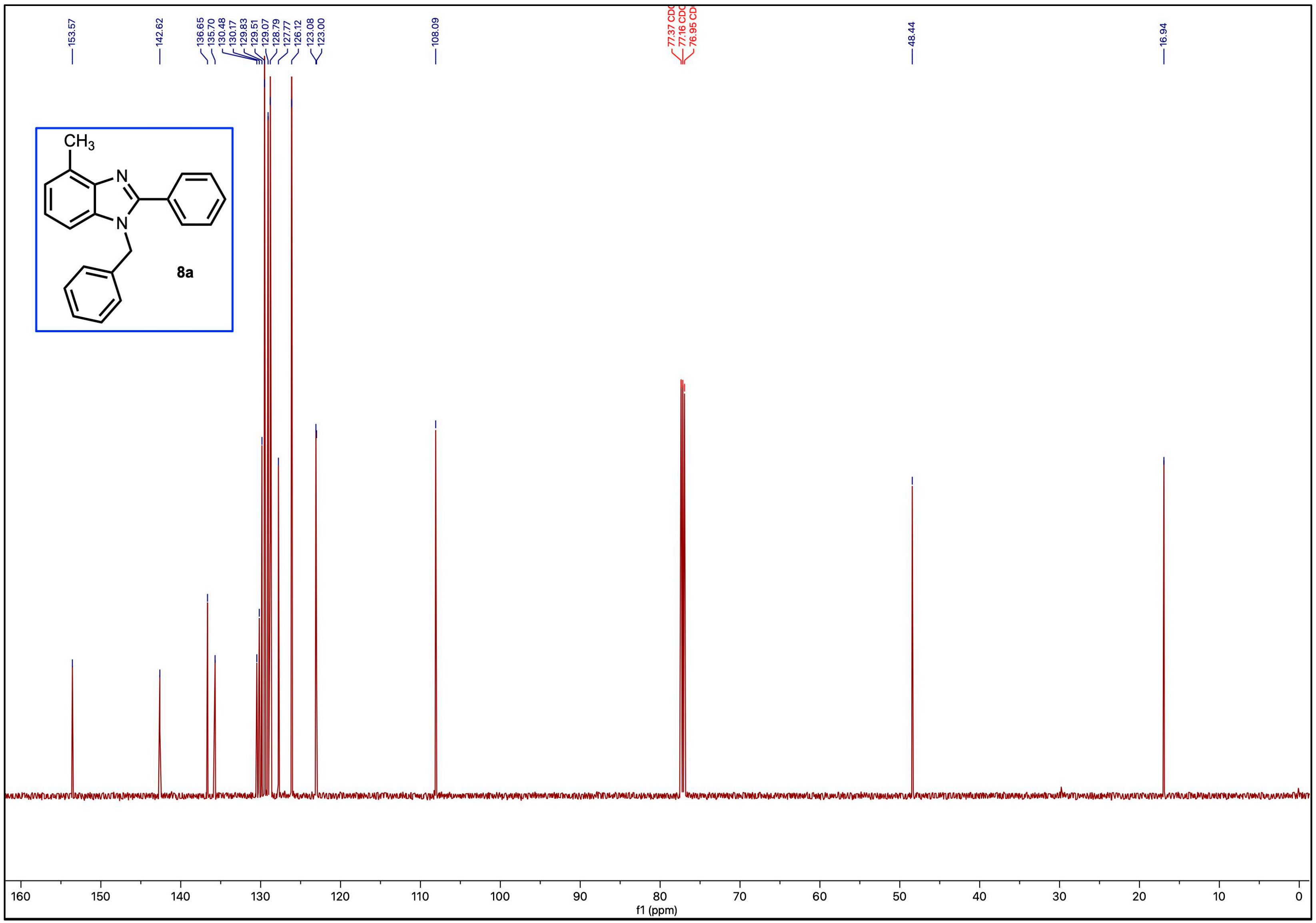

153.57
142.62
136.65
135.70
130.48
130.17
129.83
129.51
129.07
128.79
127.77
126.12
123.08
123.00
108.09
77.37 CDC
77.16 CDC
76.95 CD
48.44
16.94
CH3
N
N
8a
160
150
140
130
120
110
100
90
80
70
60
50
40
30
20
10
0
f1 (ppm)

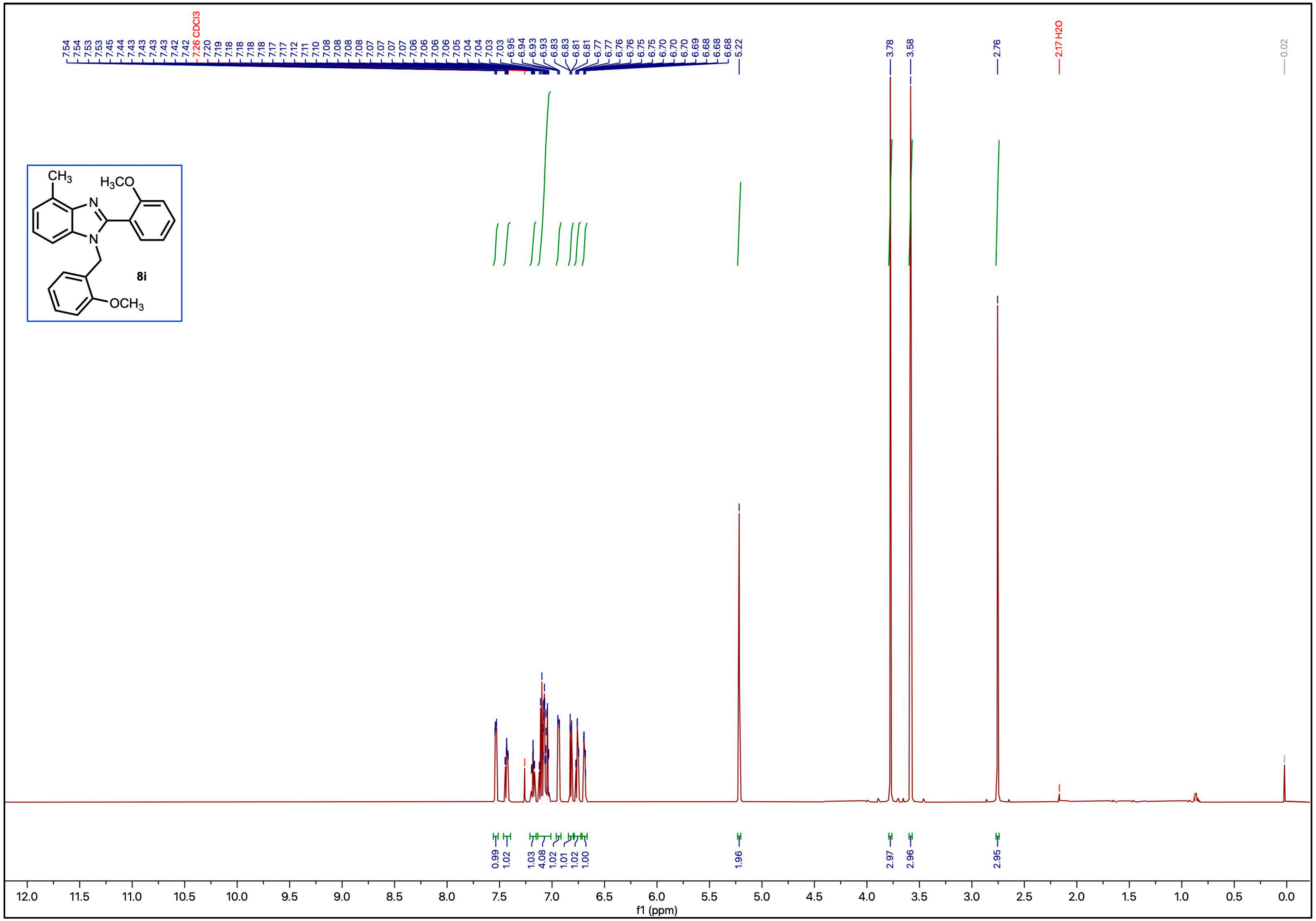
7.54
7.54
7.53
7.53
7.45
7.44
7.43
7.43
7.43
7.42
7.42
7.26 CDCl3
7.20
7.19
7.18
7.18
7.18
7.17
7.17
7.12
7.11
7.10
7.08
7.08
7.08
7.08
7.07
7.07
7.07
7.06
7.06
7.06
7.06
7.05
7.04
7.04
7.03
7.03
6.95
6.94
6.93
6.93
6.83
6.83
6.81
6.81
6.77
6.77
6.76
6.76
6.75
6.75
6.70
6.70
6.69
6.68
6.68
6.68
5.22
3.78
3.58
2.76
2.17 H2O
0.02
CH3
H3CO
N
N
OCH3
8i
0.99
1.02
1.03
4.08
1.02
1.01
1.02
1.00
1.96
2.97
2.96
2.95
12.0
11.5
11.0
10.5
10.0
9.5
9.0
8.5
8.0
7.5
7.0
6.5
6.0
5.5
5.0
4.5
4.0
3.5
3.0
2.5
2.0
1.5
1.0
0.5
0.0
f1 (ppm)

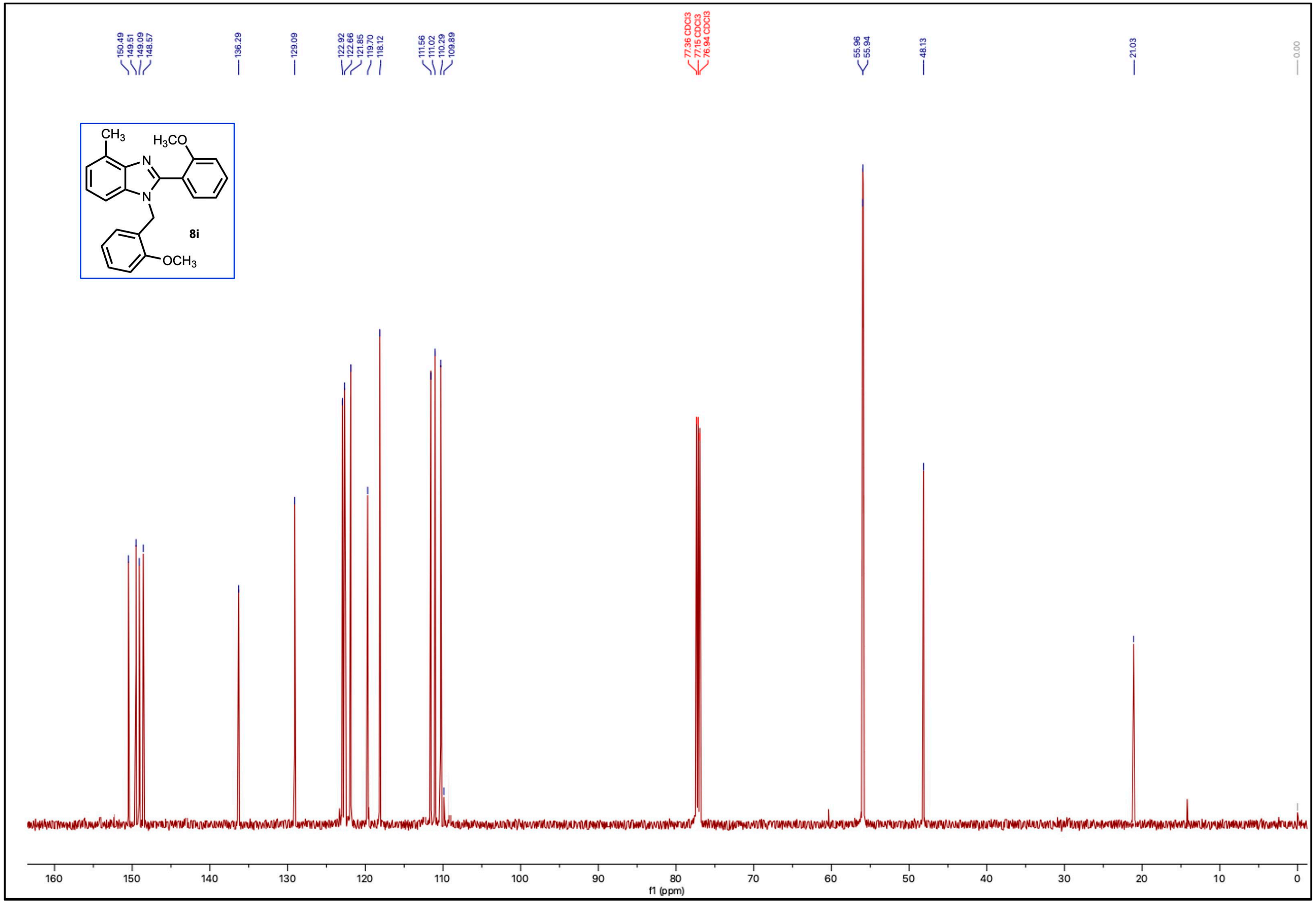
150.49
149.51
149.09
148.57
136.29
129.09
122.92
122.66
121.85
119.70
118.12
111.56
111.02
110.29
109.89
77.36 CDCl3
77.15 CDCl3
76.94 CDCl3
55.96
55.94
48.13
21.03
0.00
CH3
H3CO
N
N
8i
OCH3
f1 (ppm)

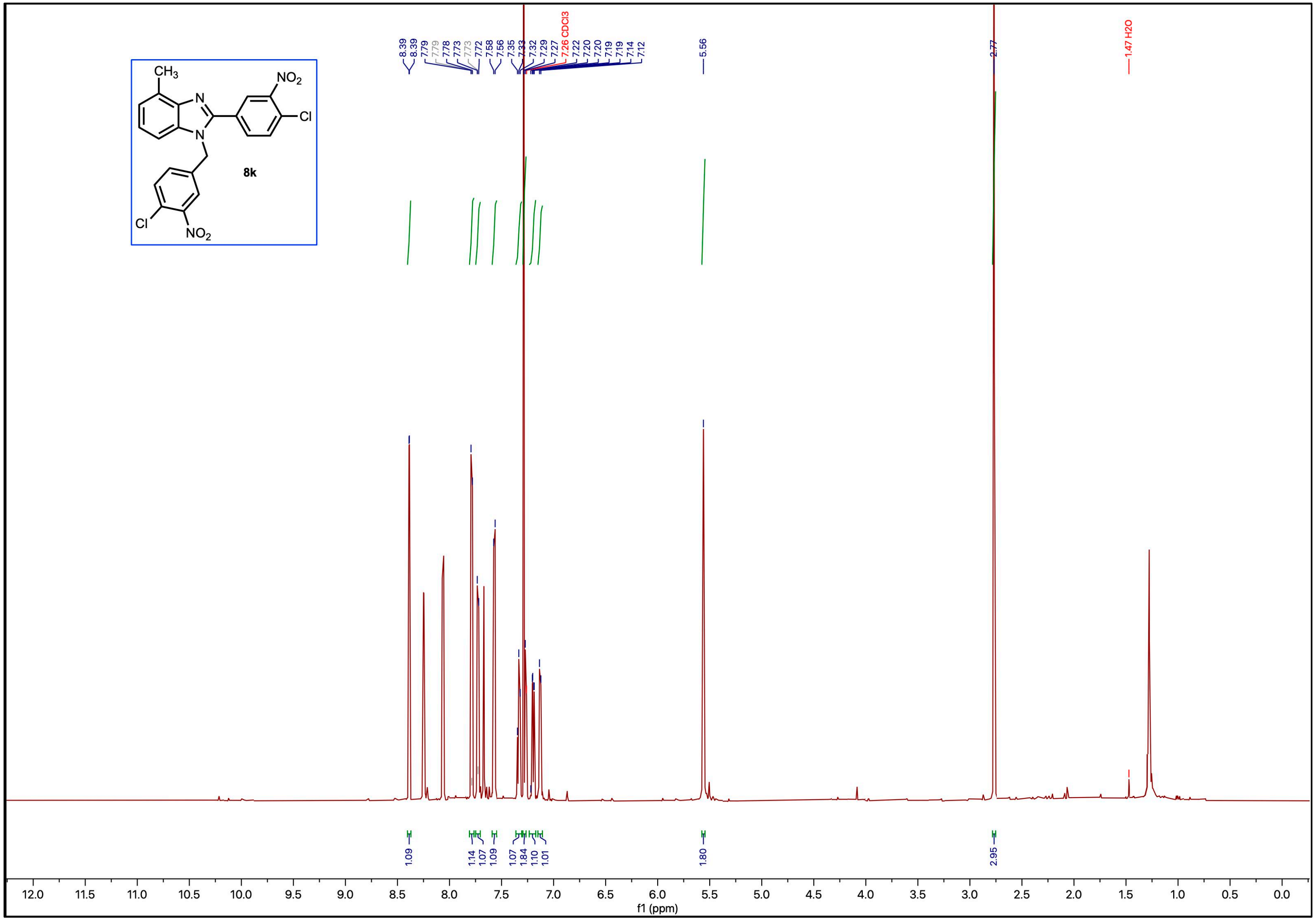
CH3
NO2
N
Cl
N
8k
Cl
NO2
8.39
8.39
7.79
7.79
7.78
7.73
7.73
7.72
7.58
7.56
7.35
7.33
7.32
7.29
7.27
7.26 CDCl3
7.22
7.20
7.20
7.19
7.19
7.14
7.12
5.56
2.77
1.47 H2O
1.09
1.14
1.07
1.09
1.07
1.84
1.10
1.01
1.80
2.95
12.0 11.5 11.0 10.5 10.0 9.5 9.0 8.5 8.0 7.5 7.0 6.5 6.0 5.5 5.0 4.5 4.0 3.5 3.0 2.5 2.0 1.5 1.0 0.5 0.0
f1 (ppm)

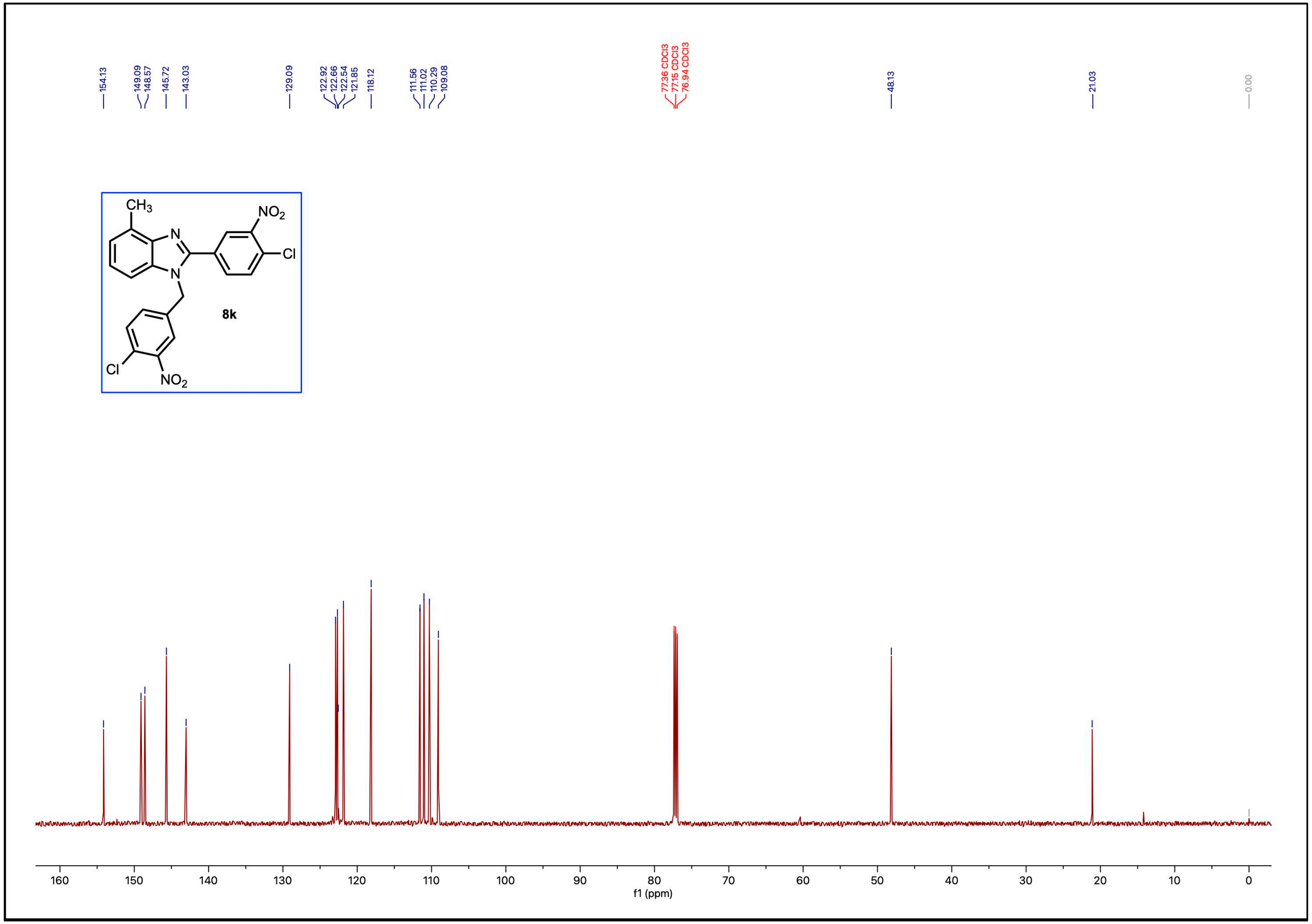
154.13
149.09
148.57
145.72
143.03
129.09
122.92
122.66
122.54
121.85
118.12
111.56
111.02
110.29
109.08
77.36 CDCl3
77.15 CDCl3
76.94 CDCl3
48.13
21.03
0.00
CH3
NO2
N
Cl
N
8k
Cl
NO2
f1 (ppm)

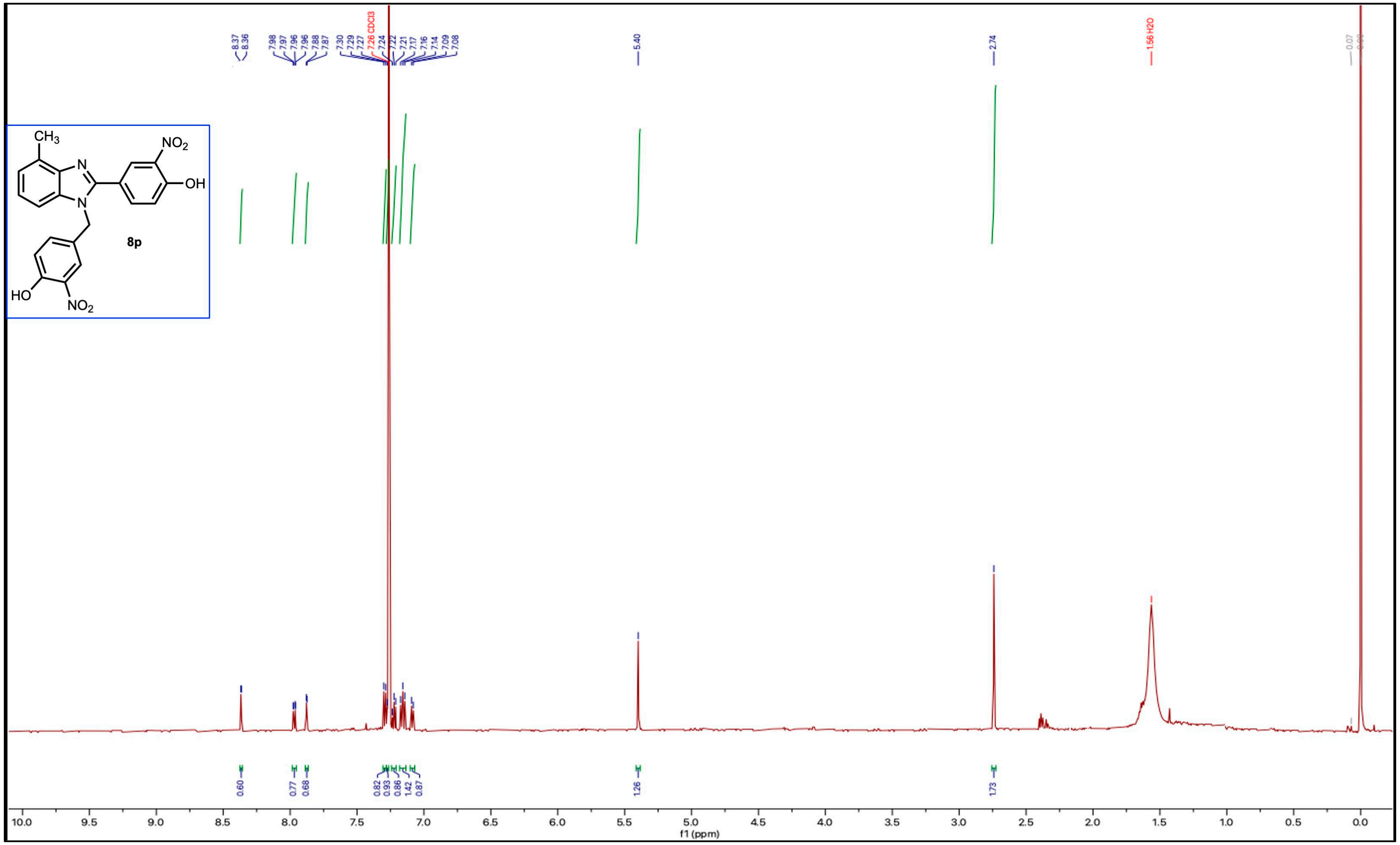
8p
f1 (ppm)

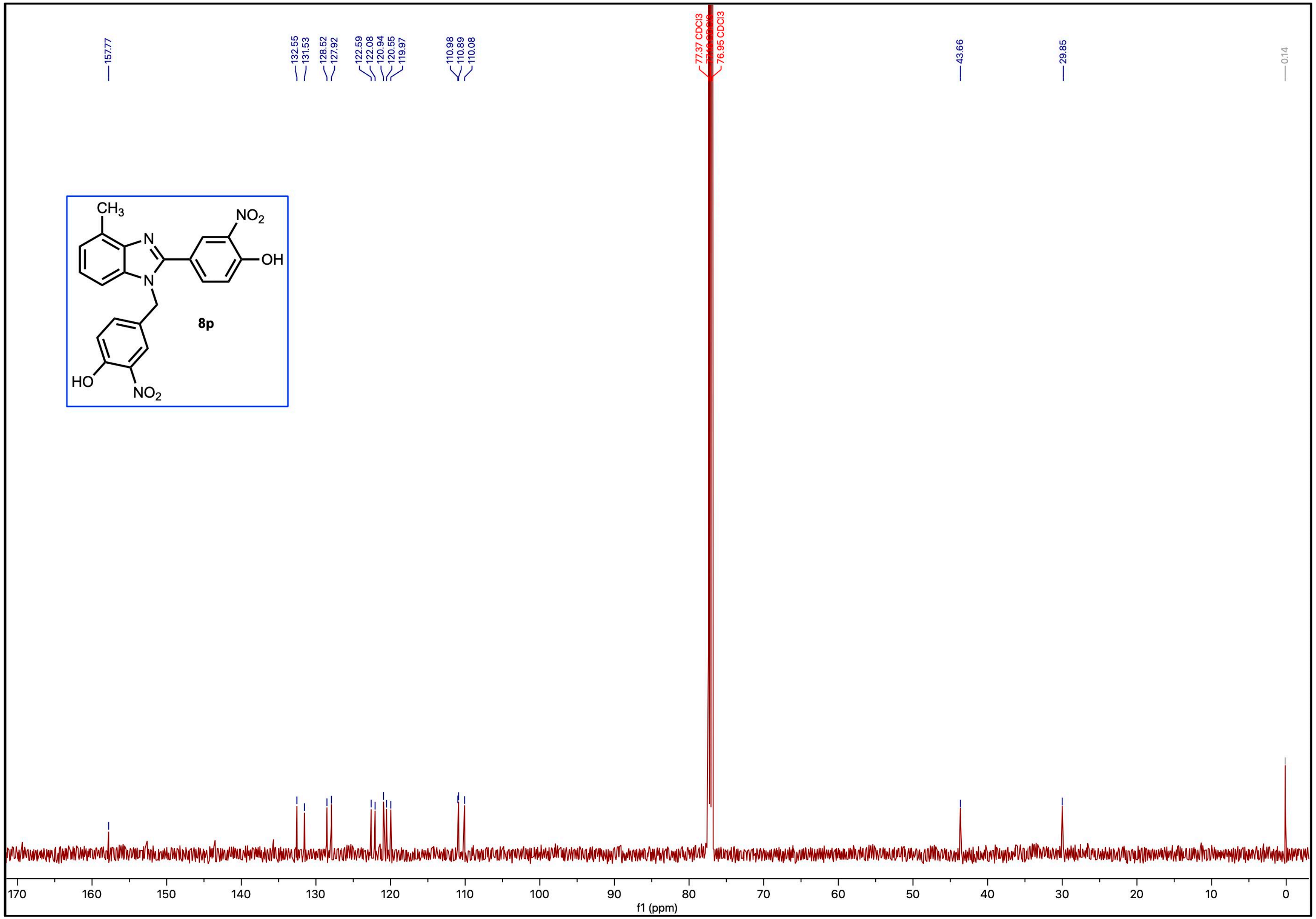

157.77
132.55
131.53
128.52
127.92
122.59
122.08
120.94
120.55
119.97
110.98
110.89
110.08
77.37 CDCl3
76.95 CDCl3
43.66
29.85
0.14
CH3
NO2
N
N
OH
HO
NO2
8p
170
160
150
140
130
120
110
100
90
80
70
60
50
40
30
20
10
0
f1 (ppm)

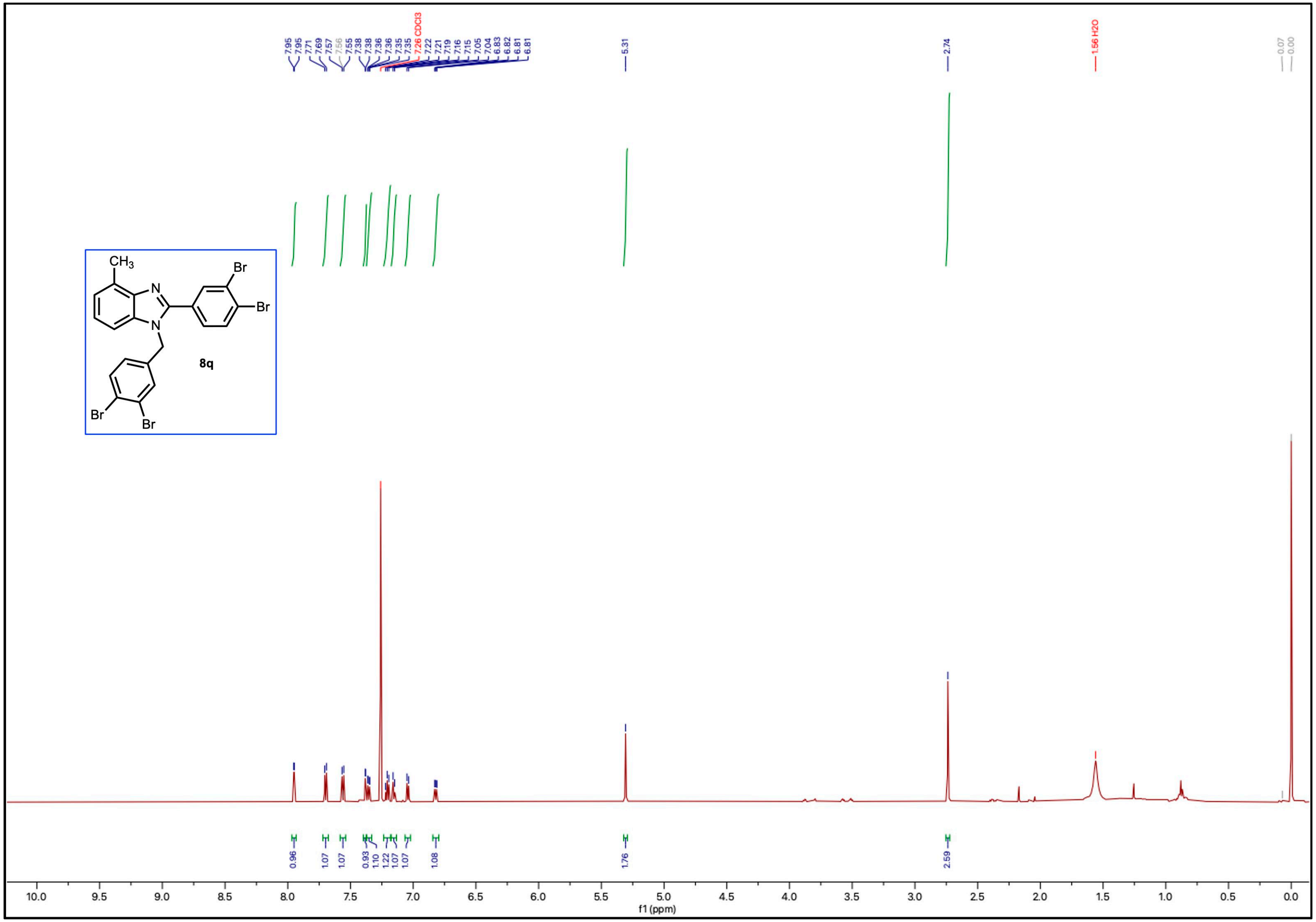

8q
7.26 CDCl3
5.31
2.74
1.56 H2O
0.07
0.00
0.96
1.07
1.07
0.93
1.10
1.22
1.07
1.07
1.08
1.76
2.59
f1 (ppm)

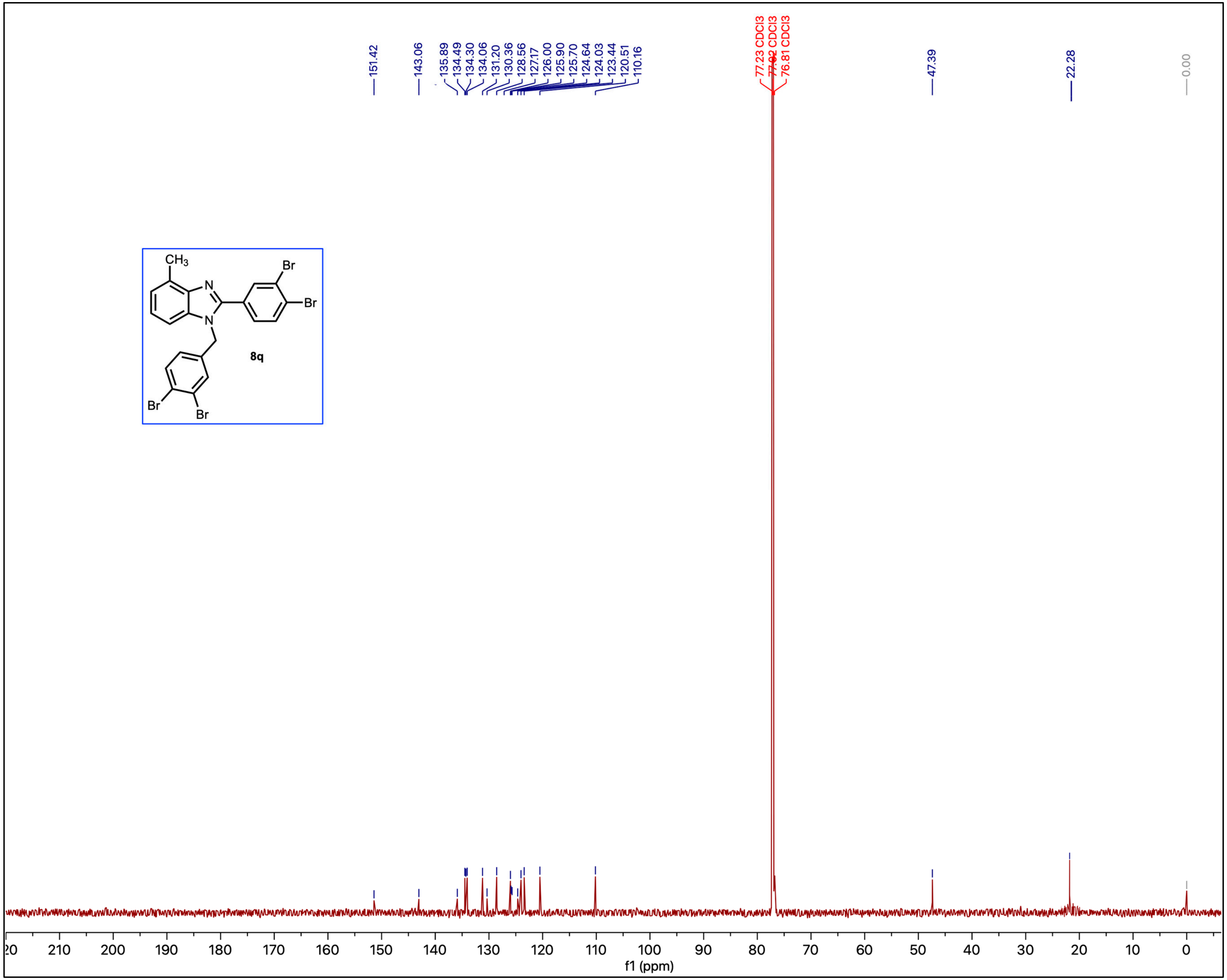
151.42
143.06
135.89
134.49
134.30
134.06
131.20
130.36
128.56
127.17
126.00
125.90
125.70
124.64
124.03
123.44
120.51
110.16
77.23 CDCl3
77.02 CDCl3
76.81 CDCl3
47.39
22.28
0.00
CH3
N
N
Br
Br
Br
Br
8q
f1 (ppm)

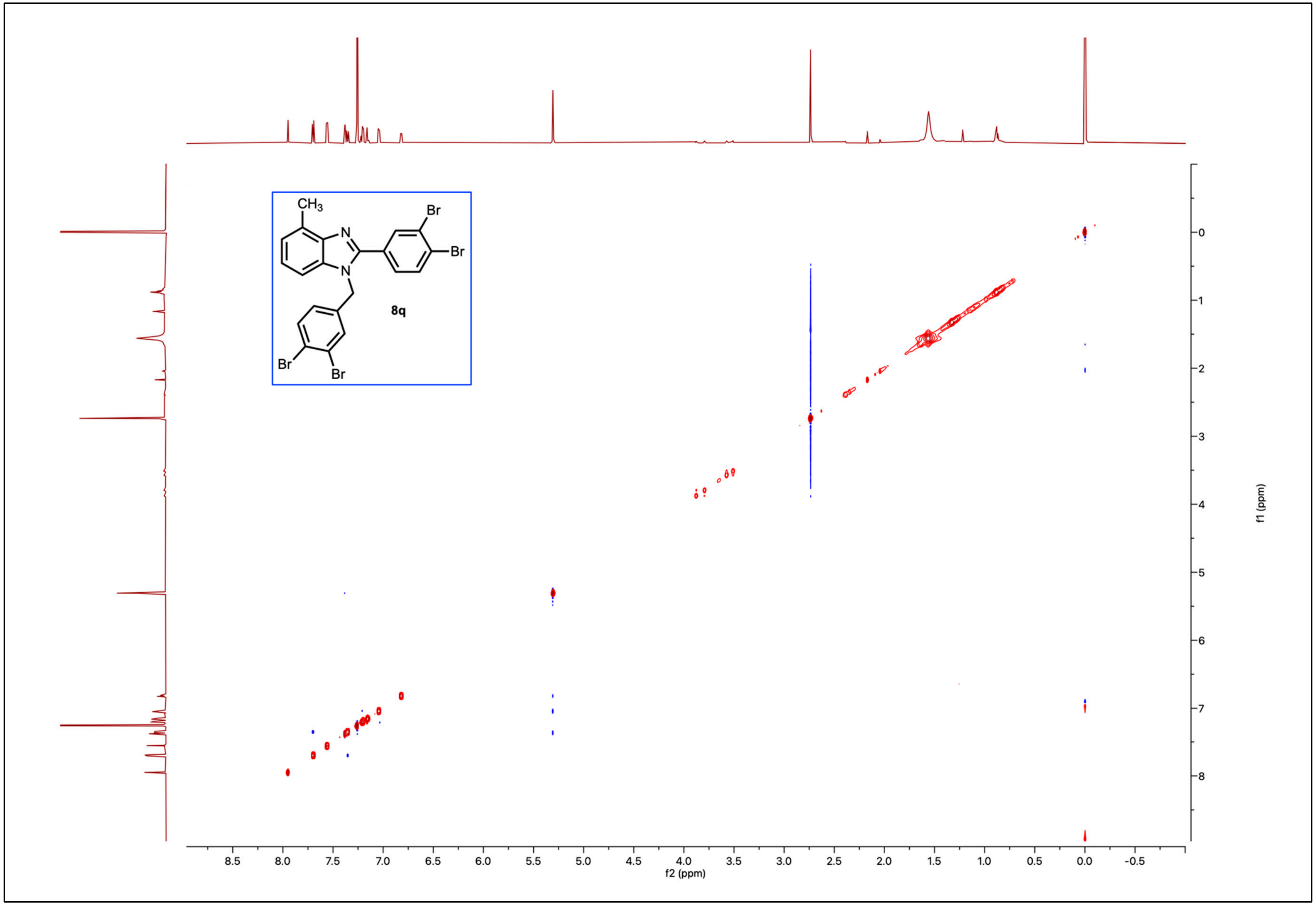

CH3
Br
N
Br
N
8q
Br
Br
8.5 8.0 7.5 7.0 6.5 6.0 5.5 5.0 4.5 4.0 3.5 3.0 2.5 2.0 1.5 1.0 0.5 0.0 -0.5
f2 (ppm)
f1 (ppm)
0 1 2 3 4 5 6 7 8

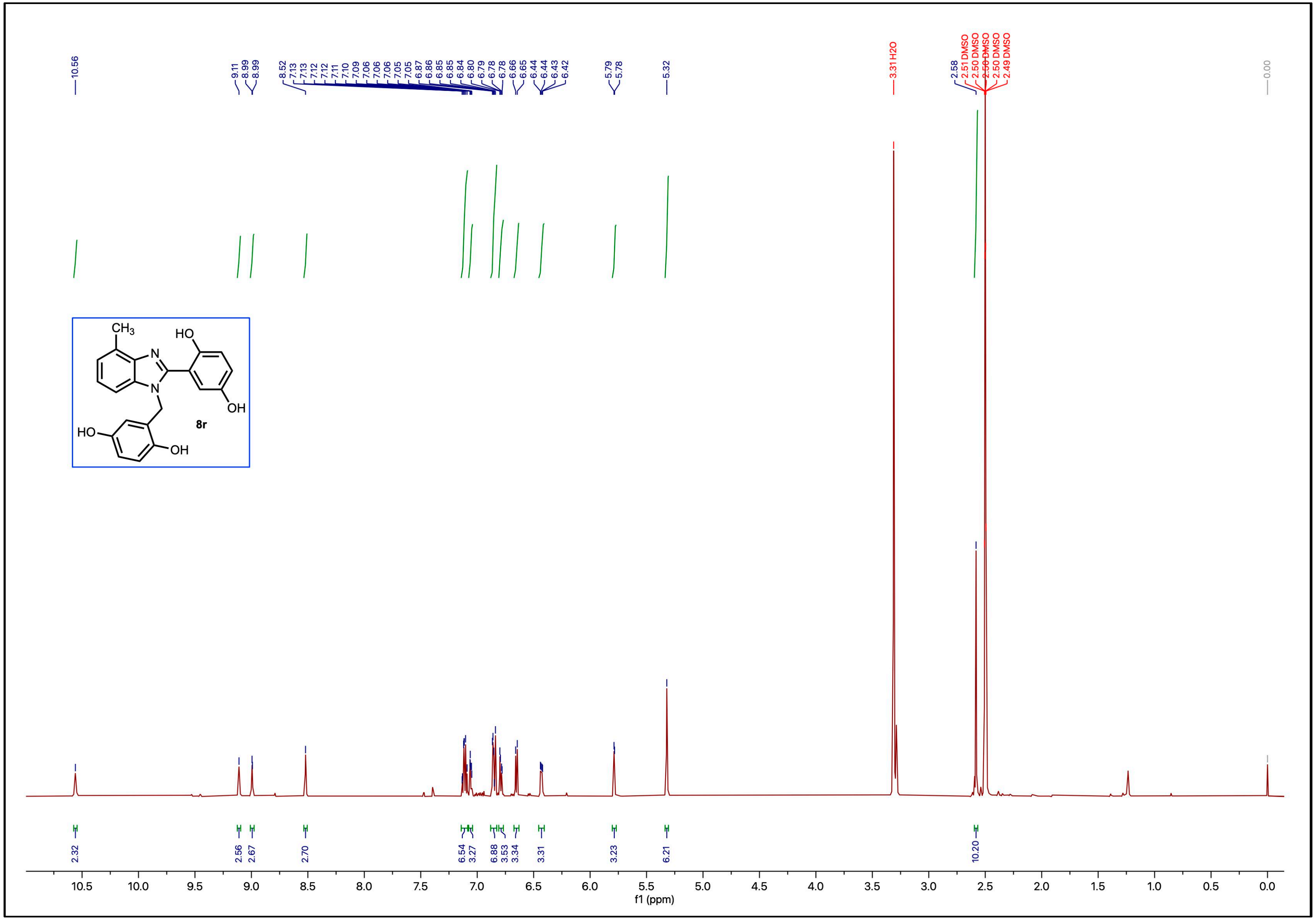

10.56
9.11
8.99
8.99
8.52
3.31 H2O
2.58
2.51 DMSO
2.50 DMSO
2.49 DMSO
0.00
5.79
5.78
5.32
CH3
HO
N
N
OH
HO
OH
8r
2.32
2.56
2.67
2.70
6.54
3.27
6.88
3.53
3.34
3.31
3.23
6.21
10.20
f1 (ppm)

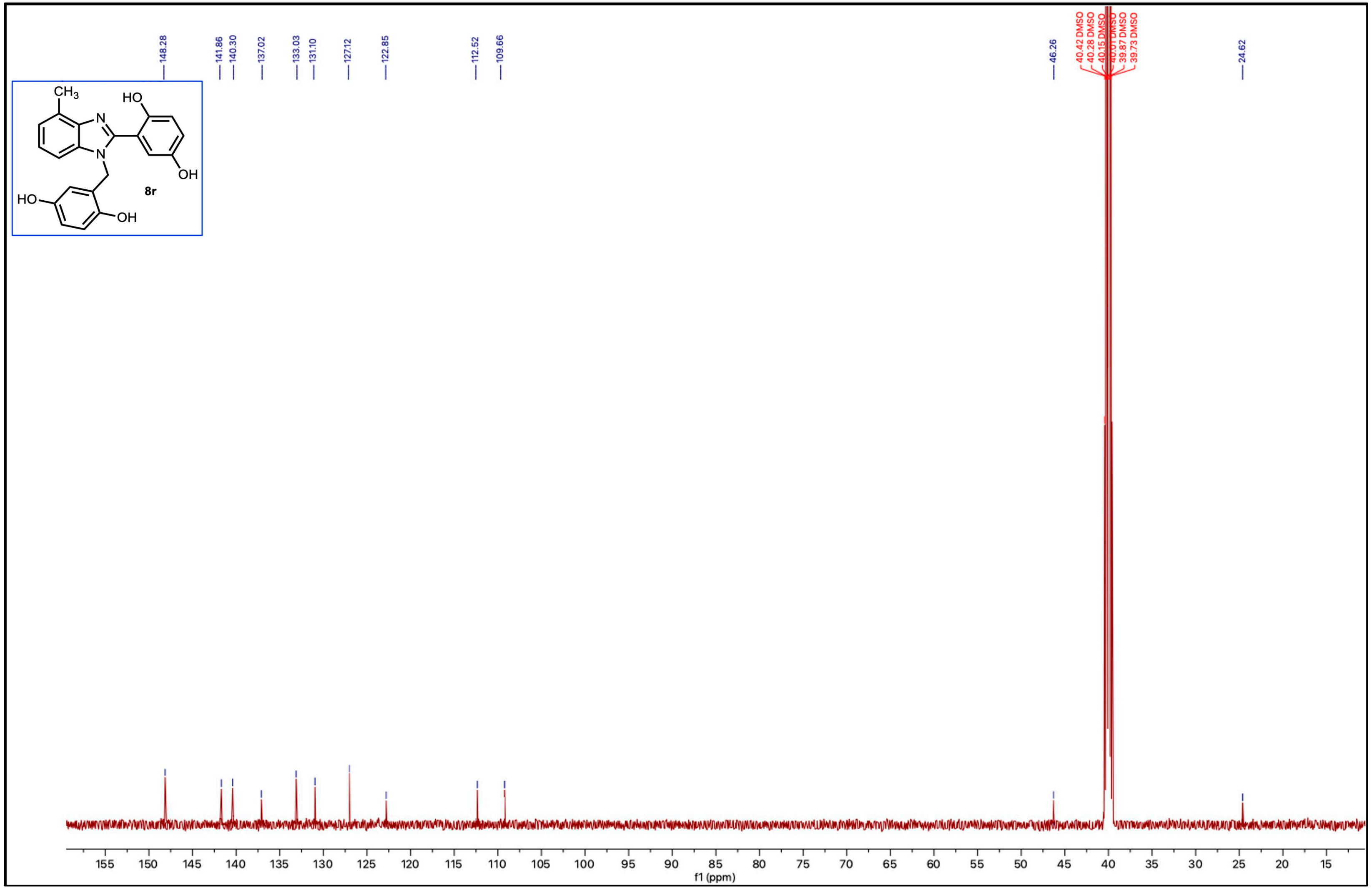
148.28
141.86
140.30
137.02
133.03
131.10
127.12
122.85
112.52
109.66
46.26
40.42 DMSO
40.28 DMSO
40.15 DMSO
39.87 DMSO
39.73 DMSO
24.62
8r
f1 (ppm)

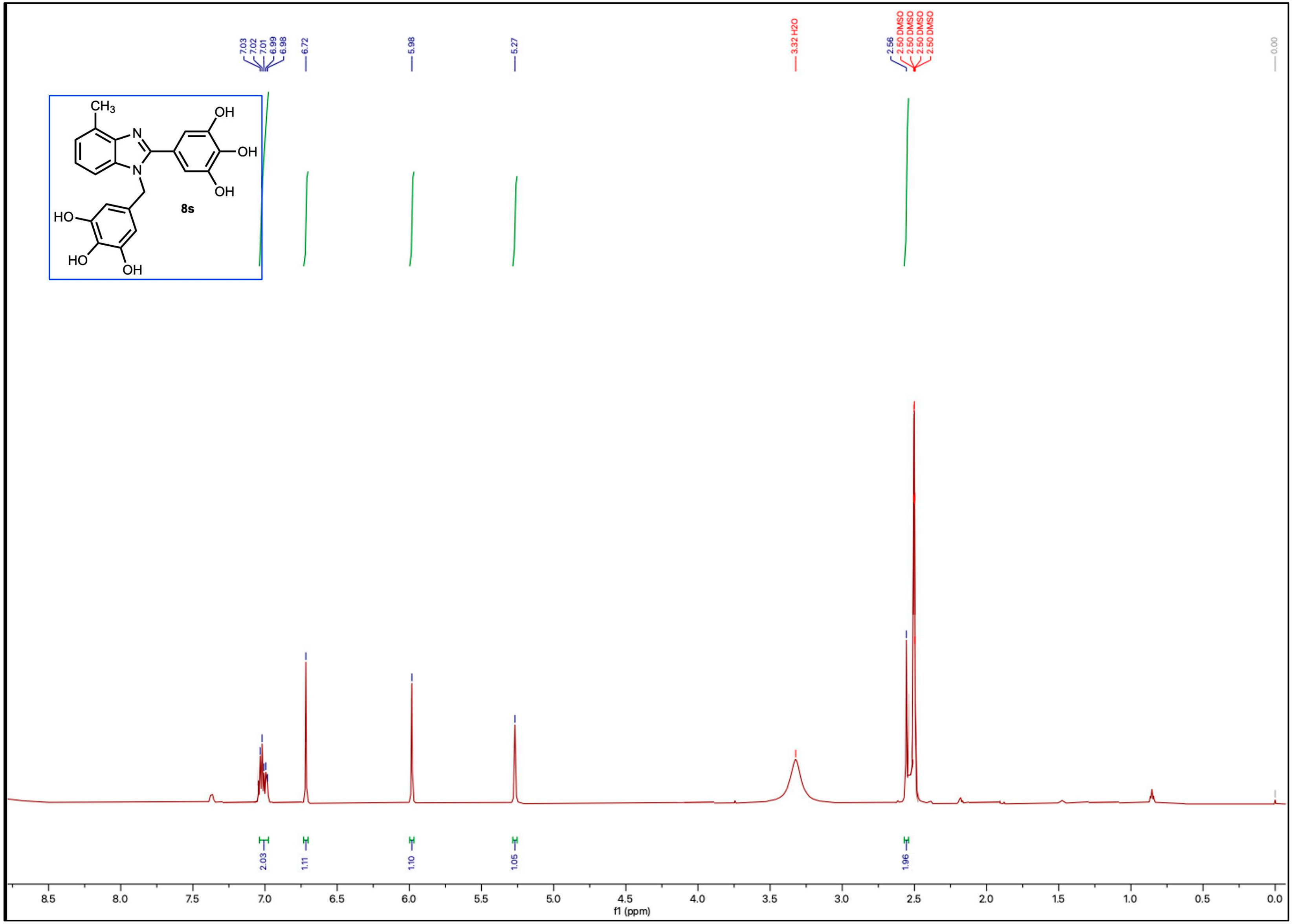

8s
7.03
7.02
7.01
6.99
6.98
6.72
5.98
5.27
3.32 H2O
2.56
2.50 DMSO
2.50 DMSO
2.50 DMSO
2.50 DMSO
2.50 DMSO
0.00
2.03
1.11
1.10
1.05
1.96
f1 (ppm)

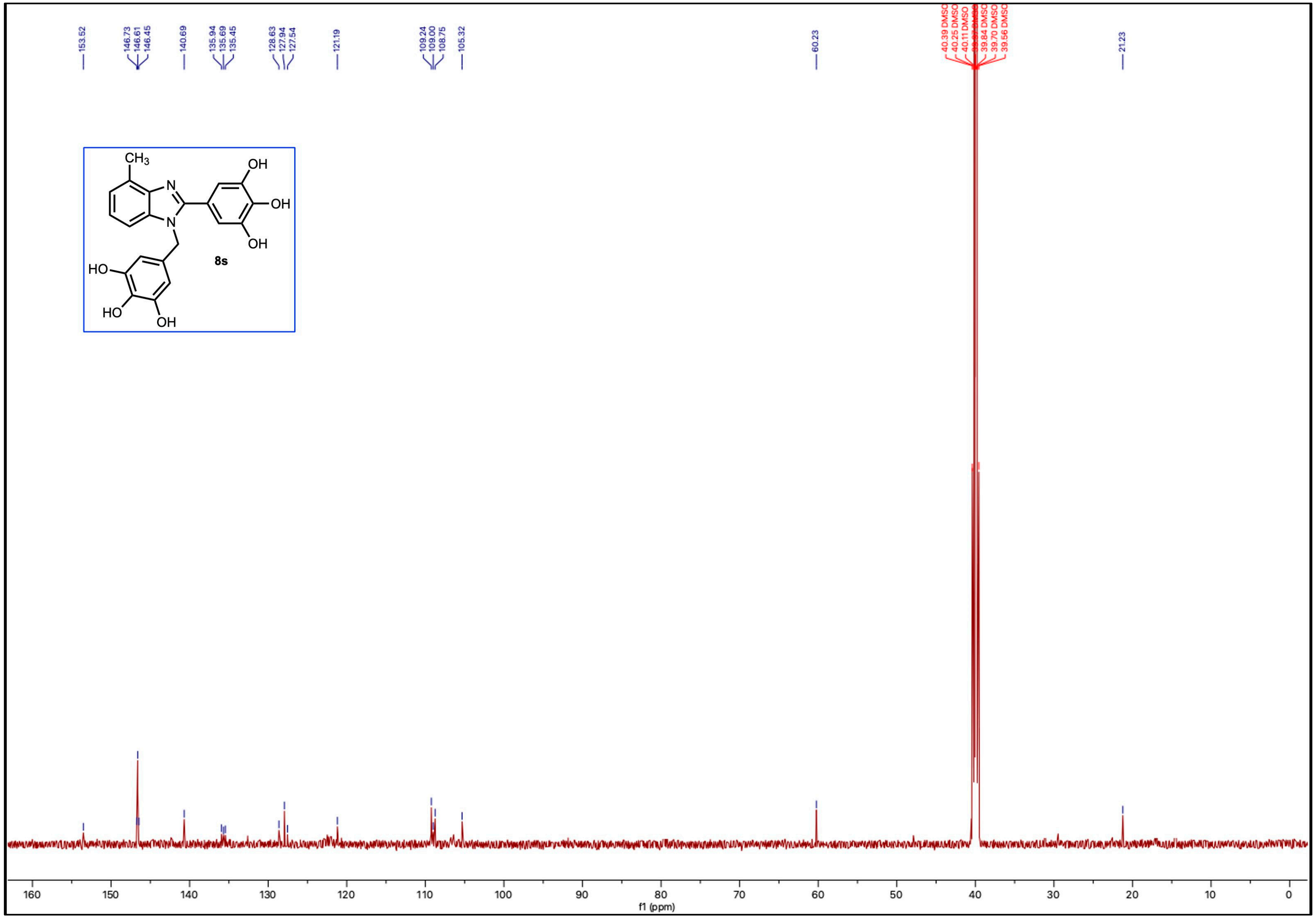

153.52
146.73
146.61
146.45
140.69
135.94
135.69
135.45
128.63
127.94
127.54
121.19
109.24
109.00
108.75
105.32
60.23
40.39 DMSO
40.25 DMSO
40.11 DMSO
39.84 DMSO
39.70 DMSO
39.56 DMSO
21.23
CH_3
N
OH
OH
OH
HO
HO
OH
8s
f1 (ppm)

8s

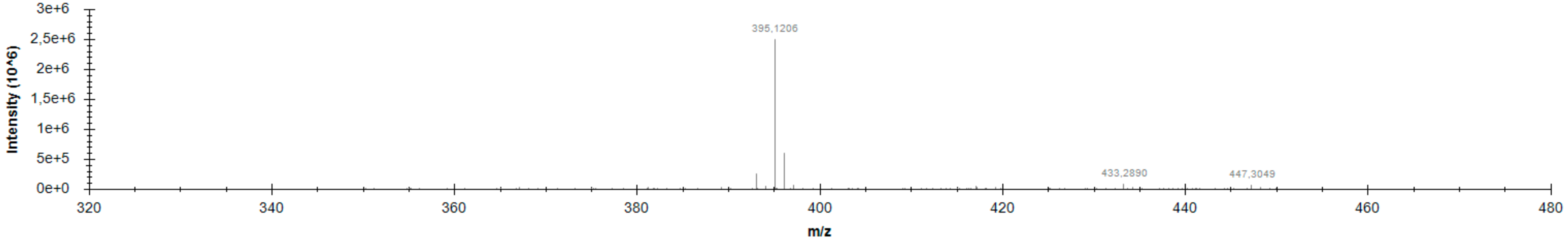

395,1206
433,2890
447,3049
3e+6
2,5e+6
2e+6
1,5e+6
1e+6
5e+5
0e+0
Intensity (10^6)
320
340
360
380
400
420
440
460
480
m/z

8k

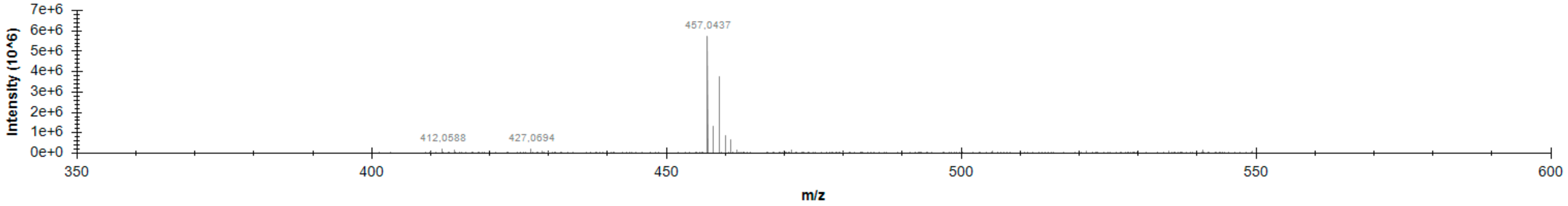

457,0437
412,0588
427,0694
7e+6
6e+6
5e+6
4e+6
3e+6
2e+6
1e+6
0e+0
Intensity (10^6)
350
400
450
500
550
600
m/z

2. *Biology*

ADMET prediction.

ADMET (absorption, distribution, metabolism, excretion, and toxicity) pharmacokinetic properties of most active compound **8s** and acarbose were assessed by means of the online PkCSM predictor [52]. Results detailed in Table 5S, indicate that **8s** possesses auspicious pharmacokinetic features similar to those associated with acarbose. The water solubility and total clearance of both compounds are within the expected parameters [53] indicating efficient absorption and normal metabolism. Notably, both compounds exhibit efficient intestinal absorption and skin permeability, have a limited ability to cross the blood-brain barrier (BBB), and do not penetrate the central nervous system (CNS). Furthermore, **8s** and acarbose are identified as substrates for P-glycoprotein and CYP3A4, both of which are essential for cellular protection from exogenous toxic agents and play a critical role in drug metabolism. This observation, along with the absence of AMES toxicity and their role as hERG I inhibitors, suggests that these compounds are not expected to show significant drug toxicity, reinforcing the conclusions drawn from experimental cytotoxicity test findings.

Table 5S. Predicted ADMET of **8s** against Acarbose.

Model	8s	Acarbose	Reference values	Unit
Water solubility	-2.894	-2.609	-6 to ~ 0	Numeric (log mol/L)
Caco2 permeability	-0.73	-0.694	>0.9 (high permeability)	Numeric (log Papp in 10-6 cm/s)
Intestinal absorption (human)	58.159	4.697	>30	Numeric (% Absorbed)
Skin permeability	-2.735	-2.735	<-2.5	Numeric (log Kp)
P-glycoprotein substrate	Yes	Yes		Categorical (Yes/No)
BBB permeability	-1.822	-2.871	>0.3 (high absorption)	Numeric (log BB)

			<-1 (low absorption)	
CNS permeability	-3.797	-7.658	>-2 (high absorption)	
			<-3 (low absorption)	Numeric (log PS)
CYP2D6 substrate	No	No	-	Categorical (Yes/No)
CYP3A4 substrate	No	No	-	Categorical (Yes/No)
CYP2D6 inhibitor	No	No	-	Categorical (Yes/No)
CYP3A4 inhibitor	No	No	-	Categorical (Yes/No)
Total clearance	0.826	0.522	0.77 to ~ 1.17	Numeric (log ml/min/kg)
AMES toxicity	No	No	-	Categorical (Yes/No)
hERG I inhibitor	No	No	-	Categorical (Yes/No)
hERG II inhibitor	Yes	Yes	-	Categorical (Yes/No)
Hepatotoxicity	No	No	-	Categorical (Yes/No)
Skin sensitization	No	No	-	Categorical (Yes/No)

[52] Pires D.E.V.; Blundell T.L.; Ascher D.B. pkCSM: Predicting Small-Molecule Pharmacokinetic and Toxicity Properties Using Graph-Based Signatures *J. Med.Chem.*, **2015**, *58*, 4066. https://doi.org/10.1021/acs.jmedchem.5b00104

[53] Fan Z. Y.; Pang W.;Yu Y. Y.; Xu S. H.; Cheng L.P. Discovery and synthesis of novel phenoxyacetate ester Schiff base α-glucosidase inhibitors *Bioorg.Chem.*,**2025**,*154*, 107952. https://doi.org/10.1016/j.bioorg.2024.107952.

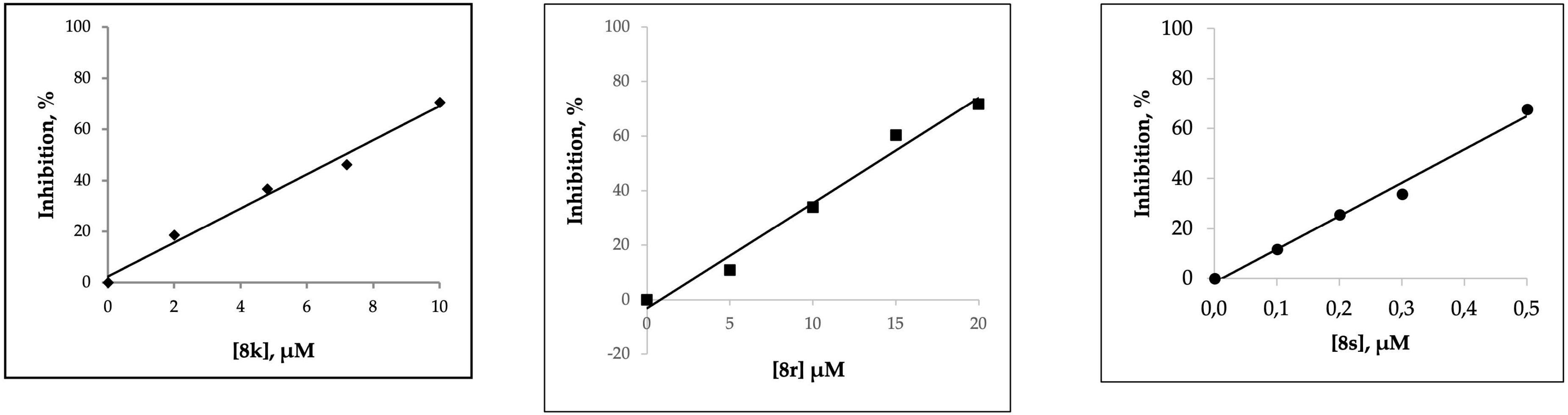

[8k], μM	% Inhibition
0	0
2	18.5
4.8	36.6
7.2	46.1
10	70.4

[8r], μM	% Inhibition
0	0
5	11.0
10	34.1
15	60.3
20	71.8

[8s], μM	% Inhibition
0	0
0.10	11.7
0.20	25.6
0.30	33.8
0.50	67.5

Figure 2S. The inhibition plots of compounds **8k**, **8r**, and **8s**.

3. *In silico* studies

8q

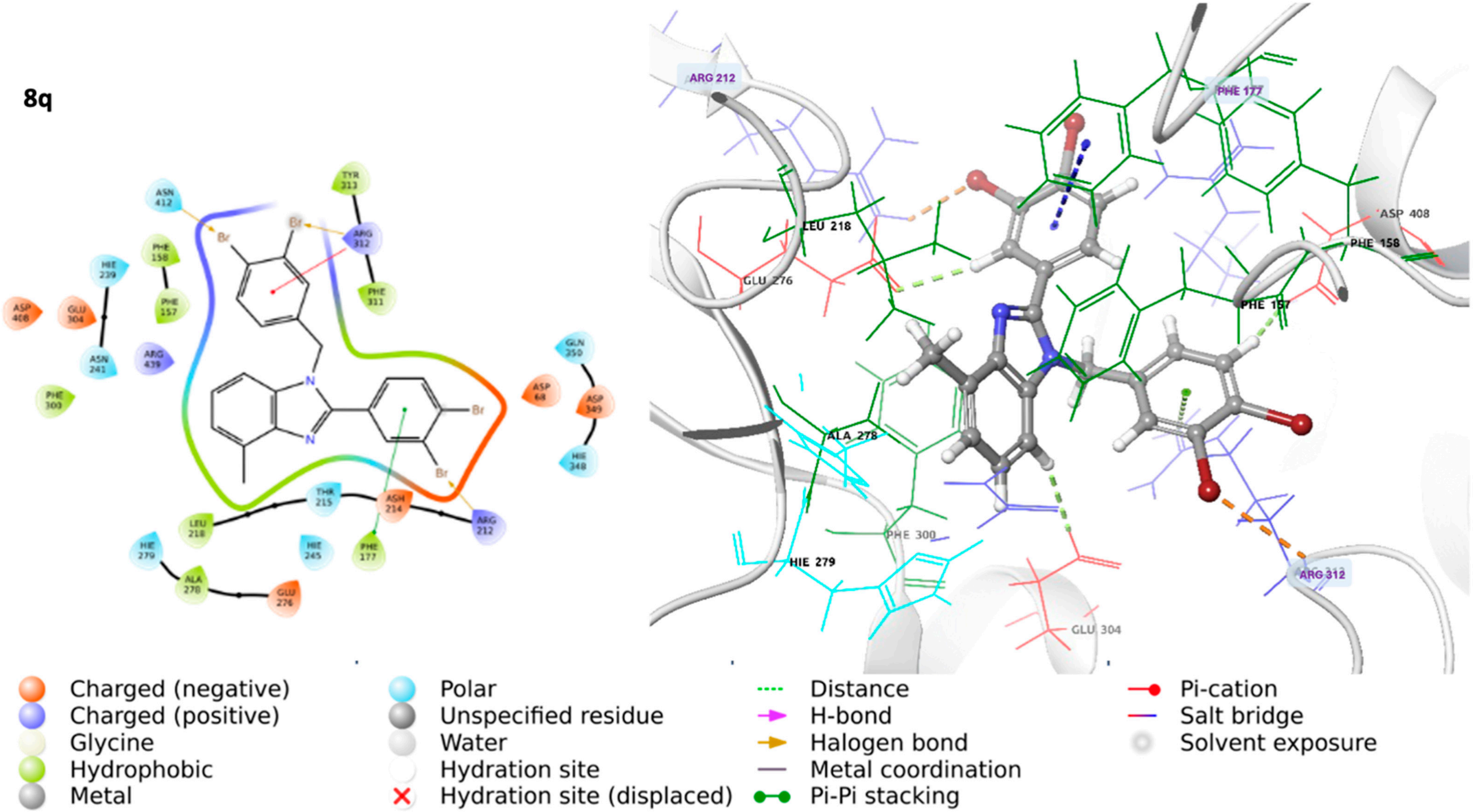

Figure 3S. 2D and 3D representations of the docking pose of compound **8q** within α-glucosidase active site

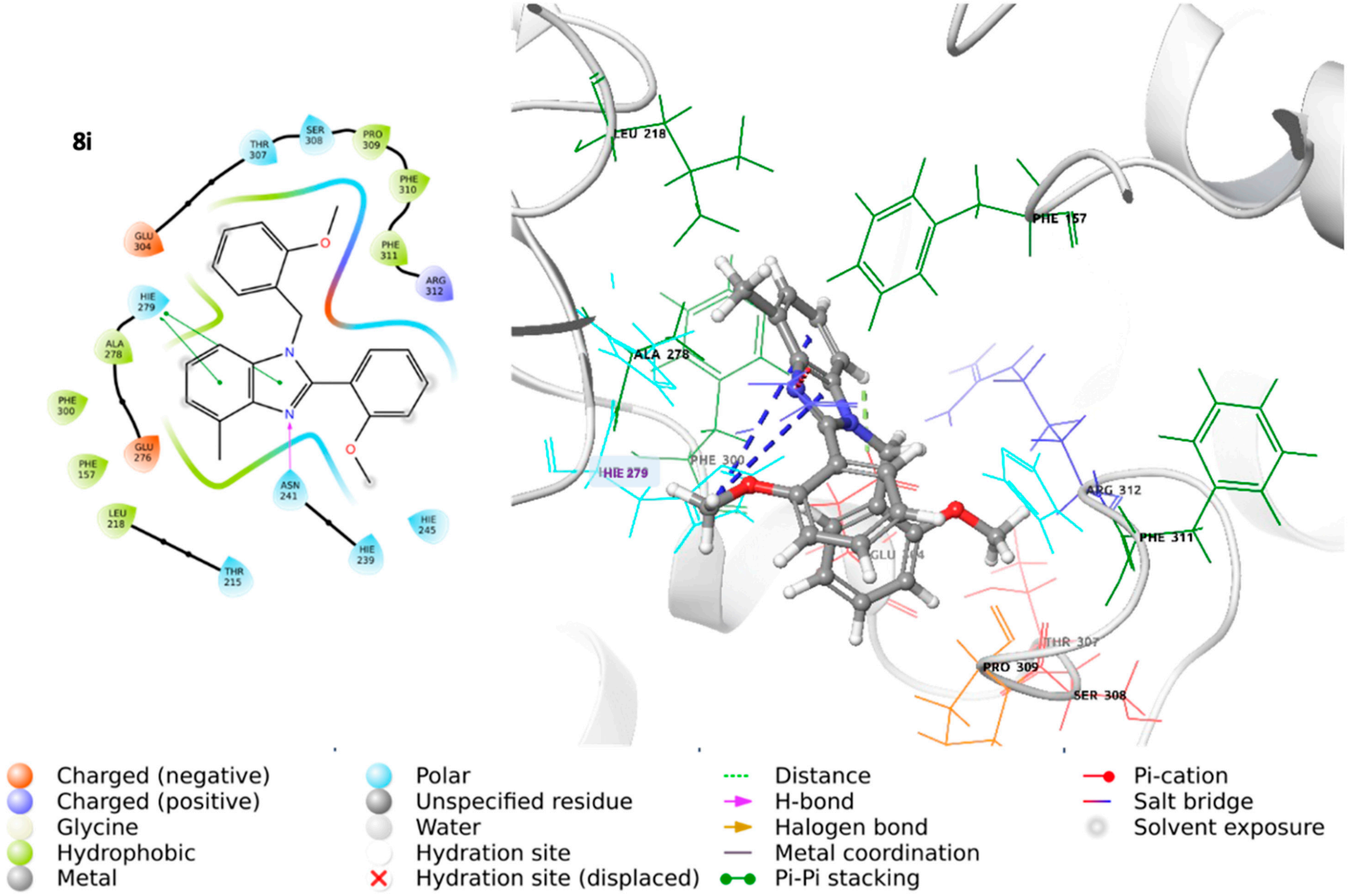

Figure 4S. 2D and 3D representations of the docking pose of compound **8i** within α-glucosidase active site.

10

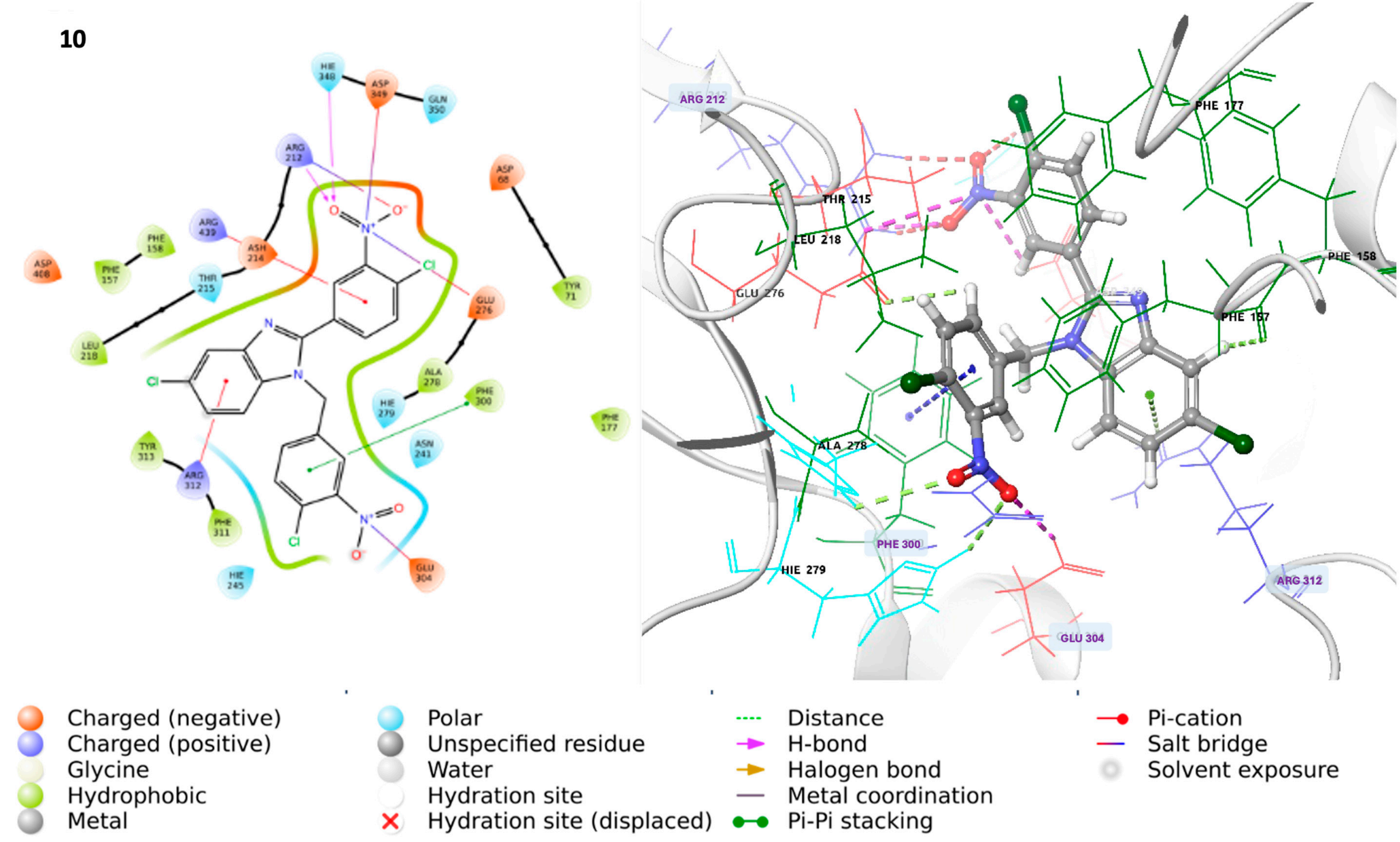

Figure 5S. 2D and 3D representations of the docking pose of compound **10** within α-glucosidase active site.

3k

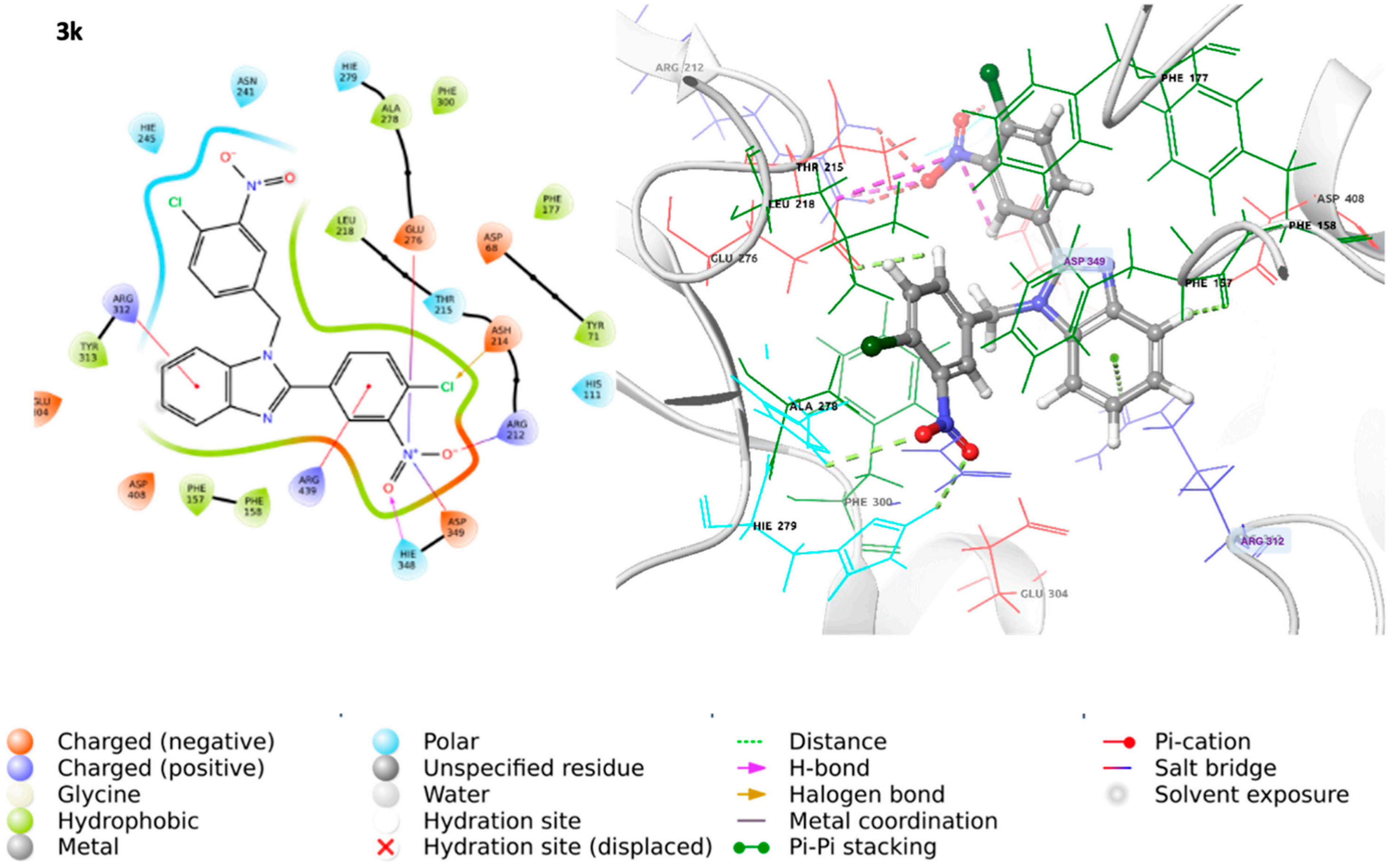

Figure 6S. 2D and 3D representations of the docking pose of compound **3k** within α-glucosidase active site

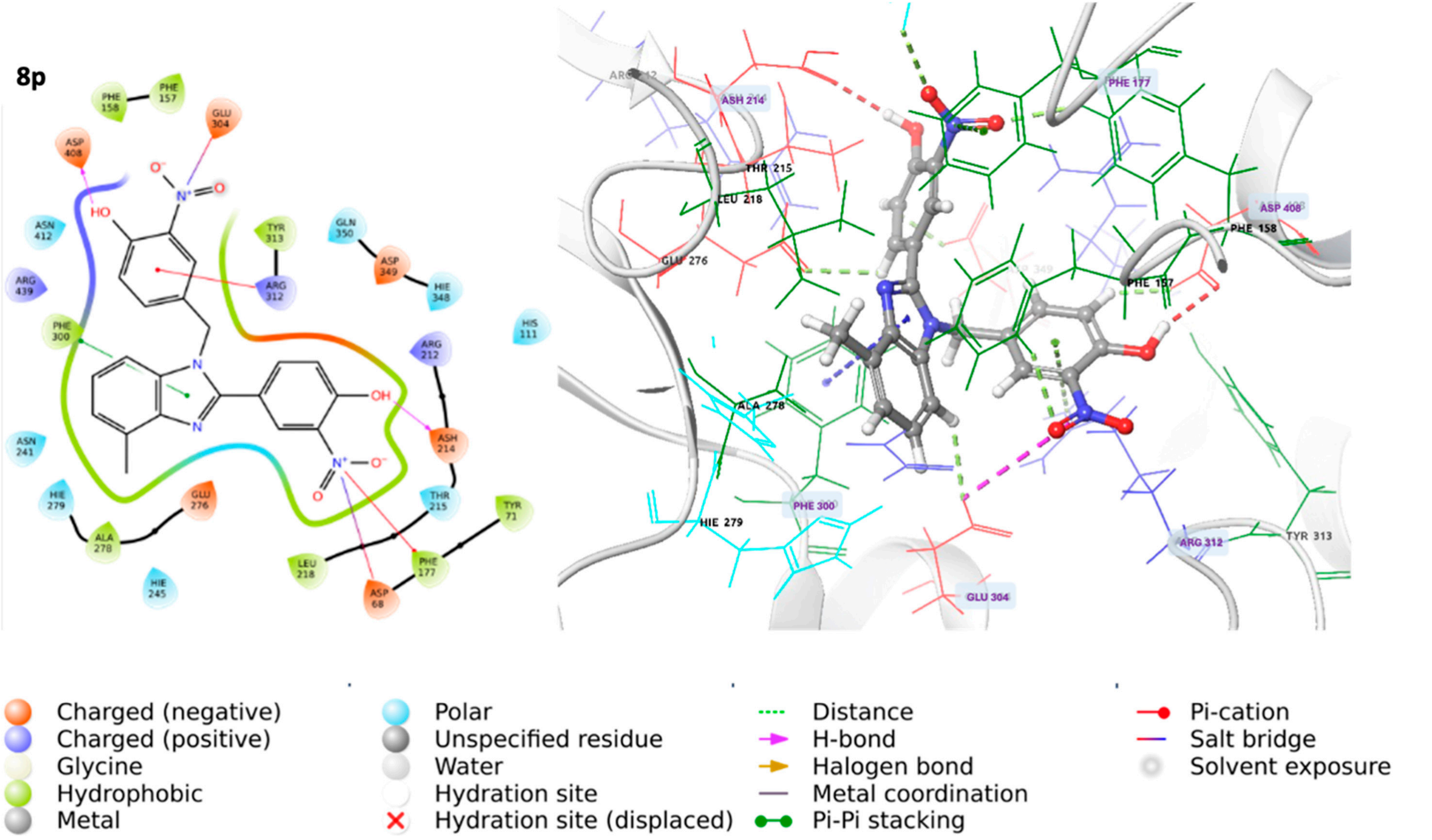

Figure 7S. 2D and 3D representations of the docking pose of compound **8p** within α-glucosidase active site.

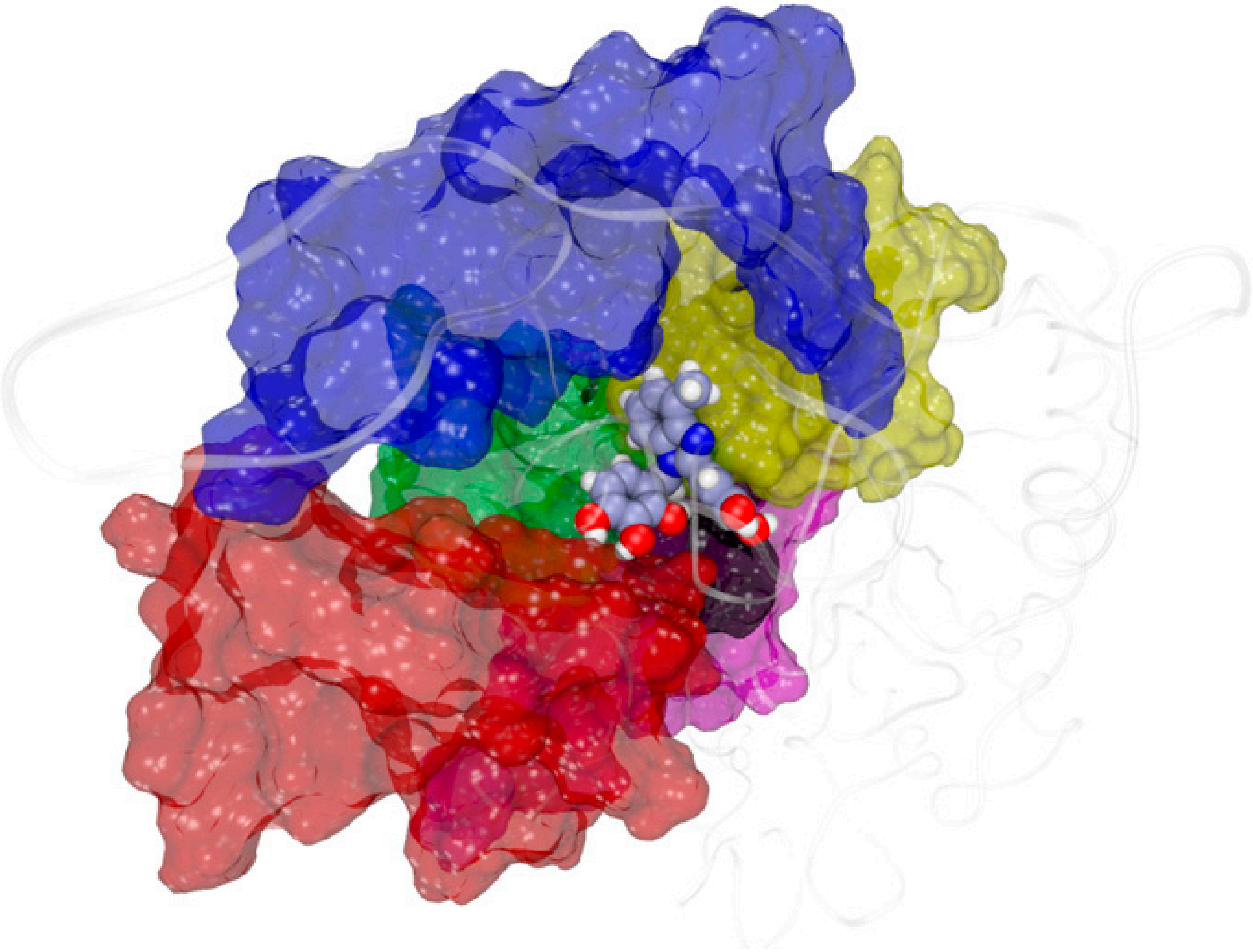

FIGURE 8S. 3D representation of regions involved in key fluctuations: blue (residues 200-250), yellow (residues 250-300), gray (residues 325-350), green (300-350), purple (350-400), and red (400-450).

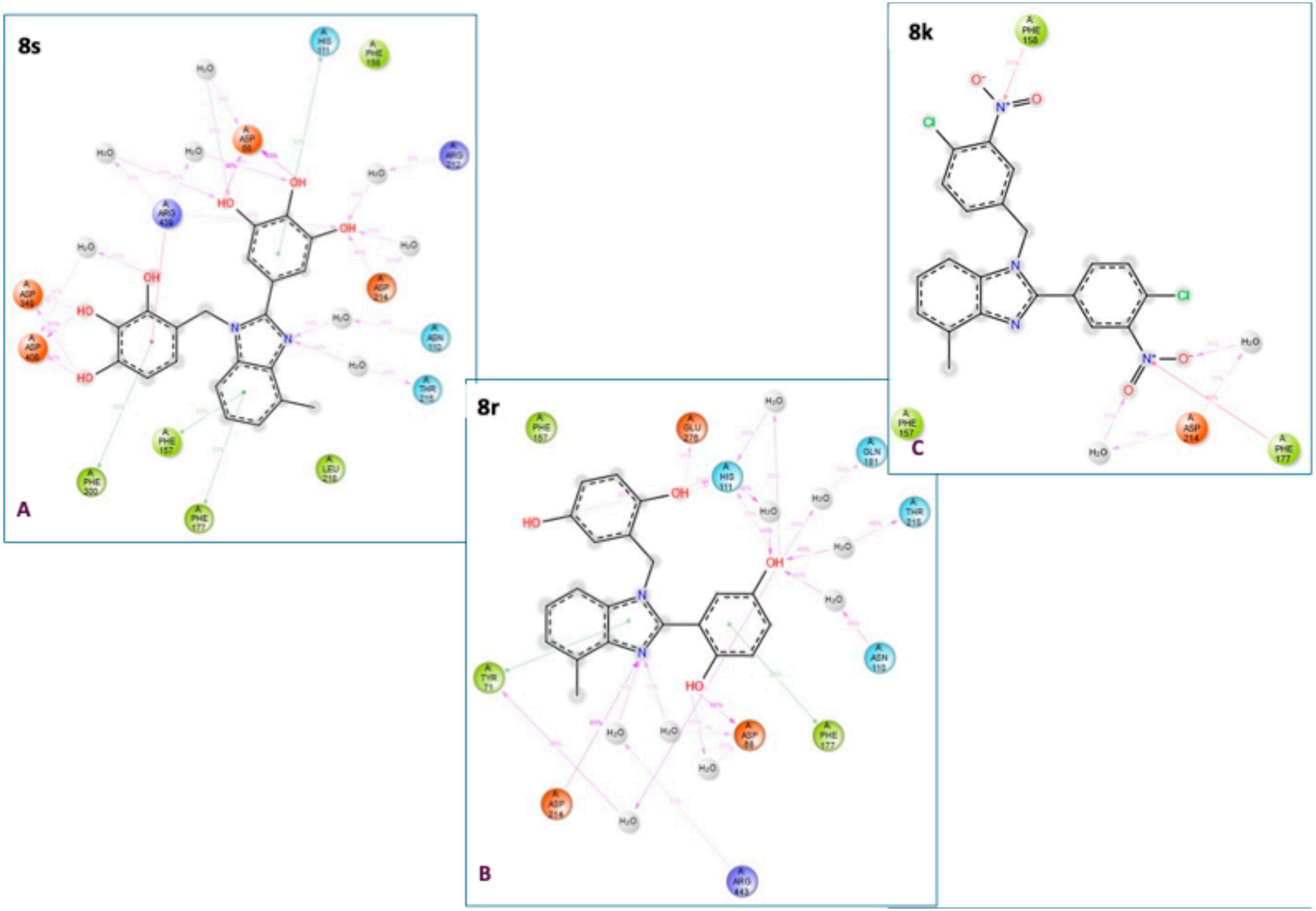

FIGURE 9S. 2D representation of the percentage of interactions that compound 8s (A), 8k (B) and 8r (C) form during the simulation. Red ball (charged positive), Blue ball (charged negative), Cyan ball (neutral), Green ball (hydrophobic), Green line (π-π), Red line (π -cation), Red-Bue line (salt bridge), Grey shadow (solvent exposure), Purple line (hydrogen bond).

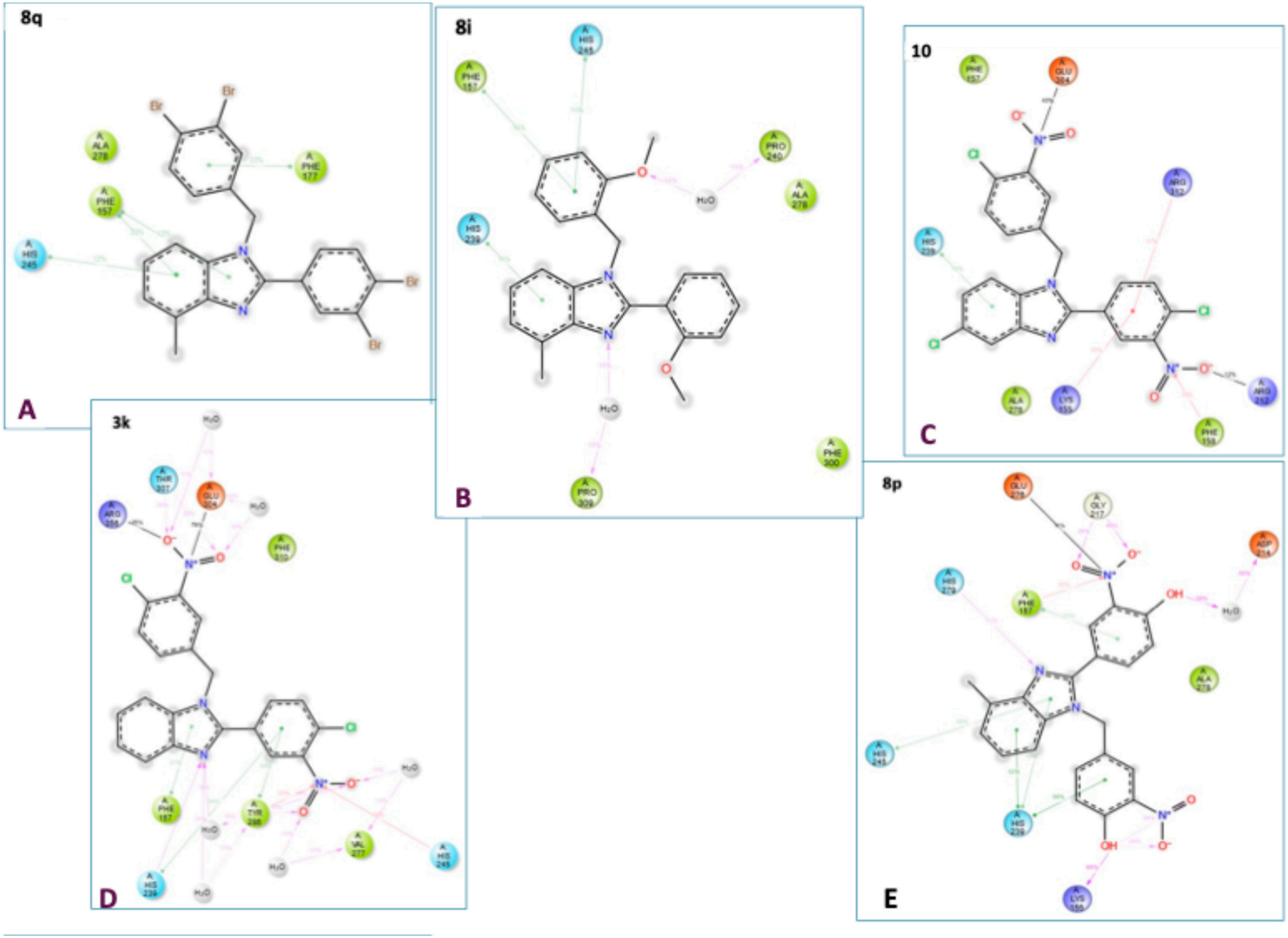

FIGURE 10S. 2D representation of the percentage of interactions that compound **8q** (A), **8i** (B), **10** (C), **3k** (D) and 8p (E) form during the simulation. Red ball (charged positive), Blue ball (charged negative), Cyan ball (neutral), Green ball (hydrophobic), Green line (π-π), Red line (π -cation), Red-Bue line (salt bridge), Grey shadow (solvent exposure), Purple line (hydrogen bond).

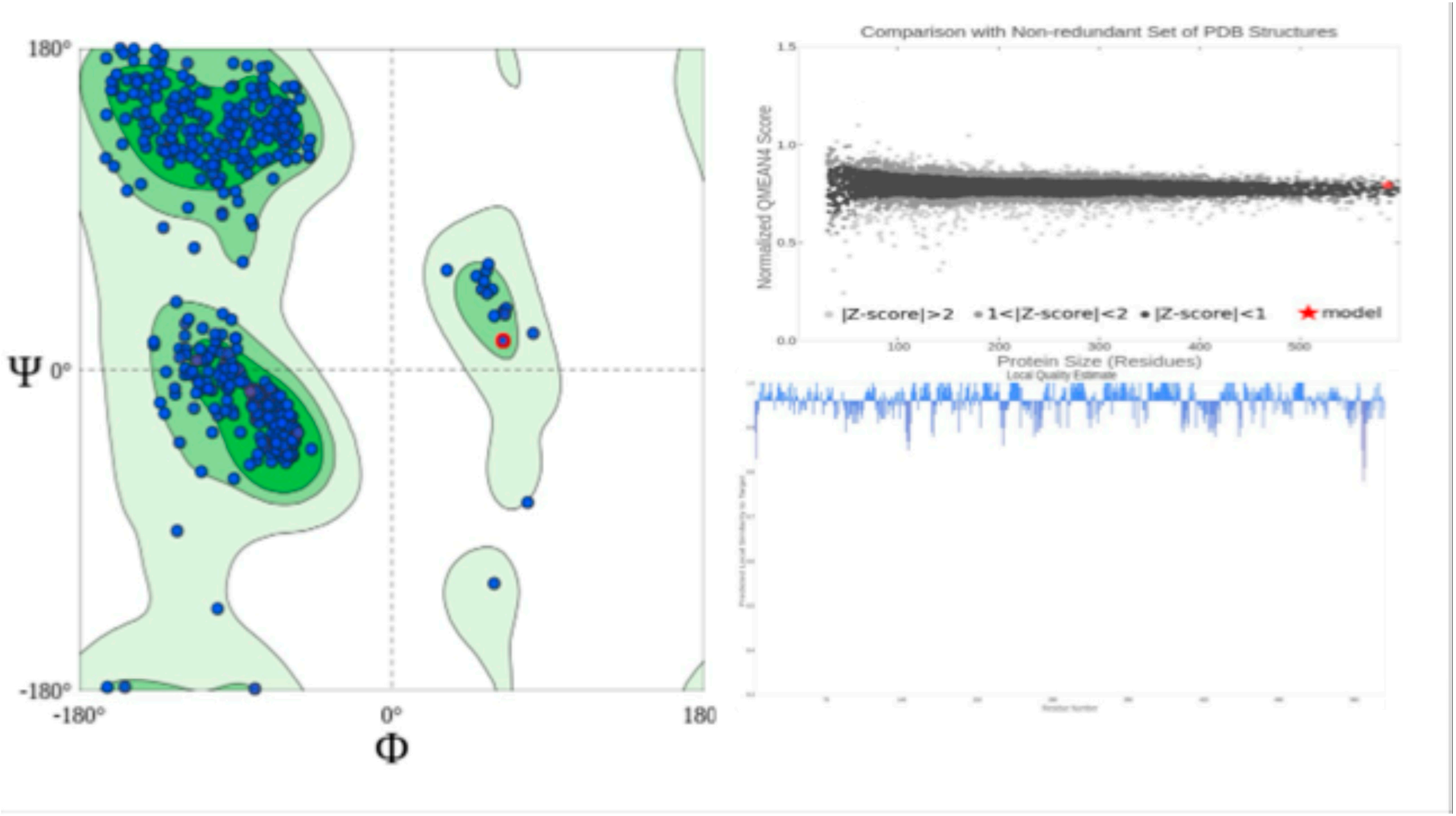

Figure 11S. Ramachandran plot of the homology model of α-glucosidase, comparison with a non-redundant set of PDB structures, and local quality estimate.

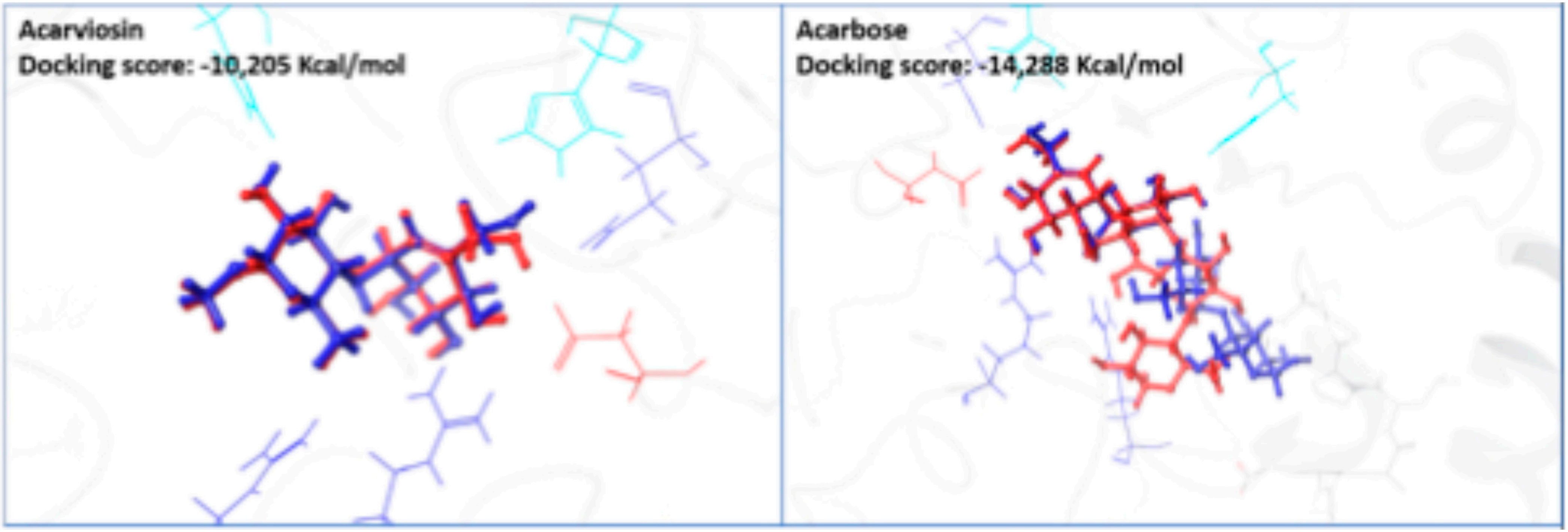

Figure 12S. Left: Overlap of acarviosin with docking score; Right: Overlap of acarbose with docking score.
